# Urazolium diacetate as a new, efficient and reusable Brønsted acid ionic liquid for the synthesis of novel derivatives of thiazolidine-4-ones†

**DOI:** 10.1039/c9ra08649h

**Published:** 2020-01-02

**Authors:** Leila Zare Fekri, Hooshang Hamidian, Masoomeh Abdollahi Chekosarani

**Affiliations:** Department of Chemistry, Payame Noor University PO Box 19395-3697 Tehran Iran chem_zare@yahoo.com chem_zare@pnu.ac.ir

## Abstract

Urazolium diacetate catalyzed synthesis of new derivatives of 1,3-thiazolidine-4-ones (azo dispersive dyes family) *via* multicomponent reaction of various aldehydes, thioglycolic acid and 4-aminoazobenzene under solvent-free reaction was reported. This avenue for the synthesis of new derivatives of thiazolidine-4-one has advantages as: short reaction times, high yields, green aspect of chemistry and environmental friendliness, easy workup, solvent-free conditions and convenient operation.

## Abstract 2

Urazolium diacetate catalyzed synthesis of new derivatives of 1,3-thiazolidine-4-ones (azo dispersive dyes family) *via* multicomponent reaction of various aldehydes, thioglycolic acid and 4-aminoazobenzene under solvent-free reaction was reported.

## Graphical abstract

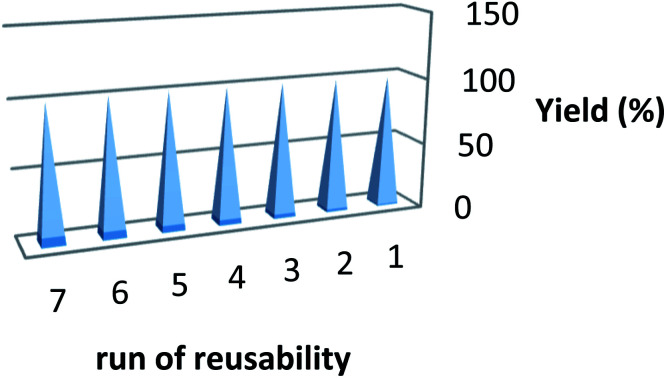

## Introduction

Thiazolidinone-4-ones have attracted a lot of interest because of their broad spectrum of biological activities such as anticancer,^1,2^ antitumor,^3,4^ antimicrobial,^5^ anti-inflammatory,^6^ anti-fungal,^7^ anti-malarial,^8^ herbicidal,^9^ anti-viral,^10^ anti-proliferative,^11^ and anti-*Toxoplasma gondii* activities.^12^ Furthermore, compounds containing a thiazolidine nucleus have properties such as anti-HIV,^13^ anti-convulsant,^14^ cycloxygenase inhibitory,^15^ anti-histaminic,^16^ anti-platelet activating factor,^17^ Ca^2+^ channel blocker,^18^ anti-oxidant,^19,20^ anti-tubercular,^21^ and analgesic^22^ properties.

Several methods for the synthesis of 4-thiazolidinones are widely reported in the literature. The main synthetic routes to synthesize 1,3-thiazolidin-4-ones involve three components reaction between amine, a carbonyl compound and a mercapto-acid.^23–25^ The other protocols for the synthesis of thiazolidine-4-ones include (1) one-step cyclization reaction between ethyl 5-phenylthioureido-3H-imidazole-4-carboxylate and bromoacetic acid to afford (imidazolylimino)thiazolidinones,^26^ (2) the reaction of aryl or alkyl isothiocyanate with a primary amine followed by treating with halo acetic acid to give 2-imino-thiazolidin-4-ones,^27^ (3) coupling reaction between α-chloro amide derivatives with isothiocyanate in the presence of a mild base to afford the iminothiazolidinone derivatives,^28^ (4) the reaction between *N*-aryl-*N*-acyl thioureas and dimethyl acetylenedicarboxylate for the preparation of thiazolidine-5-ylidenes under microwave condition,^29^ (5) multicomponent synthesis of thiazolidinones using ethyl 3-aminopropionate hydrochloride, aldehydes and thioglycolic acid,^29^, (6) the treatment of (4-methyl-2-oxo-2H-chromen-7-yloxy)acetic acid hydrazide, aldehydes and thioglycolic acid in the presence of ZnCl_2_ under refluxing in dioxane,^30^ the multicomponent synthesis of thiazolidinones under microwave reaction using various anilines, aldehydes and thioglycolic acid,^29^ the reaction between *N*-aryl-2-chloroquinolin-3-yl-azomethine and thioglycolic acid in the presence of zeolite under microwave irradiation.^29^

One of the twelve principles of green chemistry is avoiding of the use of auxiliary substances such as solvents and separation agents.^31^ The toxic and hazardous properties of many solvents suppose crucial environmental concerns such as atmospheric emissions and contamination of water effluents. It is recognized that the use of nonconventional solvents as alternatives for environmentally unfriendly traditional solvents can reduce waste solvent production and hence reduce the negative impact on environment.^32^ The most prevalent of these alternative solvents are water, supercritical fluids (like supercritical CO_2_), ionic liquids and solvent-free processes.^33,34^

Among the proposed nonconventional solvents, the use of ionic liquids in organic synthesis is interesting. Also, functional ionic liquids referred “task specific ionic liquids (TSIL)” are developing.^35^ The term of TSIL or functionalized ionic liquids actually indicates the ability of ionic liquids to act as catalyst and media, both. The application of acidic (Brønsted as well as Lewis) task specific ionic liquids (TSILs) as a catalytic system is growing rapidly in the field of catalysis.^35,36^ Combining the useful characteristics of solid acids and mineral acids, reusable TSILs have been synthesized to be applied instead of the traditional harmful mineral liquid acids, such as hydrochloric acid and sulphuric acid in the chemical reactions. However, these aspects have inadequately led many stakeholders to qualify ILs as ‘green solvents’. This qualification was stated without sufficient caution about versatility of ILs. Chemical structures which could induce significant variation in risk profiles, for example, inherently combined hydrophilicity/hydrophobicity profile of ILs and limited chemical stability of some common formulations of interest might favor diffuse or accidental contamination of aquatic environment through interactions of effluents or due to accidental spills. Linked to the increase of applications and the risk of environmental contamination, growing concerns have been raised on their potential environmental and health risks. Recent studies have focused on risks of ILs using various aquatic organisms and some of them have highlighted the interest to address their immunotoxicity.^37–41^

In view of the importance of the thiazolidinone nucleus, there is a lot of interest to accommodate the new generation of this heterocyclic moiety together with introduction a novel task specific ionic liquid “urazolium diacetate”.

## Result and discussion

As a part of our previous interest towards the synthesize new heterocyclic and pharmaceutical compounds^42–49^ and introduction new avenues and catalysts^50–52^ in the organic transformations, here, we report a facile, green, new and efficient task specific ionic liquid urazolium diacetate for the synthesize novel thiazolidine-4-ones through three component reaction of various aldehydes, thioglycolic acid and 4-aminoazobenzene for the first time.

**Scheme 1:**
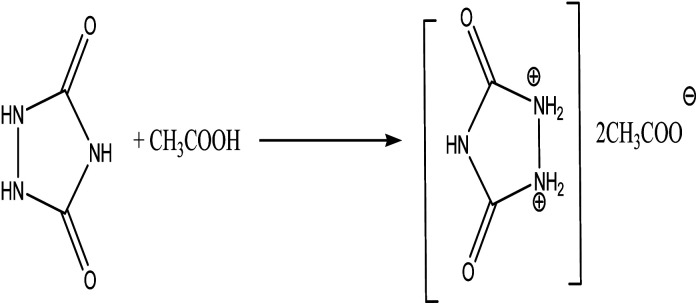
Synthesis of ionic liquid urazolium diacetate.

Initially, TSIL urazolium diacetate was synthesized by the reaction between urazole and excess acetic acid under heating at 80 °C for 4 h (Scheme 1). The yellow-orange-like TSIL was washed with 3 × 10 mL diethylether to departure of unreacted materials. After drying of the TSIL under vacuum, the synthesized urazoliumdiacetate IL was analyzed and characterized by FT-IR and ^1^H NMR. In the FT-IR spectrum, the carbonyl moiety of acetate and urazolium was appeared at 1787 cm^−1^ as a strong and slightly broad peak because of overlap between two corresponding carbonyls and, also the unreacted NH group was appeared at 3270 cm^−1^. In ^1^H NMR spectrum, the hydrogen atoms of methyl in acetate was shown as a singlet at 2.33 ppm and 5 hydrogen atoms of dicationic piece of ionic liquid were appeared as a singlet at 10.65 ppm with integral equal to 4. One hydrogen atom in the dicationic section of IL was exchanged with broadening in the spectra base line.

**Scheme 2:**
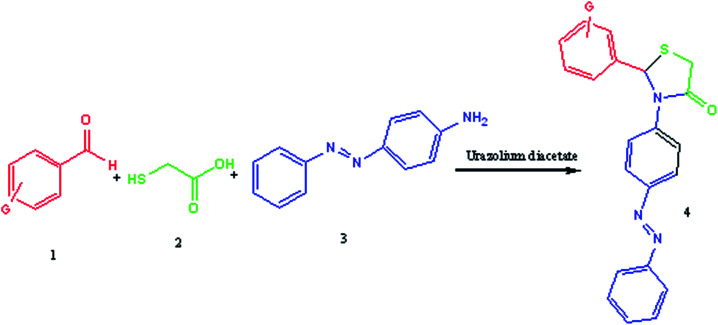
Urazolium diacetate catalyzed synthesis of azo dispersive dyes-thiazolidine.

The activity of the ionic liquid as a catalyst was then investigated by employing it in the multicomponent synthesis of new derivatives of thiazolidine-4-ones (Scheme 2).

To check the efficiency of urazoliumdiacetate in this reaction, 4-nitrobenzaldehyde (1.0 mmol), thioglycolic acid (1.0 mmol), and 4-aminoazobenzene (1.0 mmol) was attempted in different catalysts under stirring and also in various solvents. None of the desired 2-(4-nitrophenyl)-3-(4-(phenyldiazenyl)phenyl)thiazolidin-4-one 4a was obtained after stirring the reaction mixture for 24 h at room temperature in the absence of catalyst (Table 1, entry 1). The reactions were carried out in the presence of various catalysts as HCl, K-10, HY-zeolite, Fe_3_O_4_, [BMIM]Br and [DBU] OAc and show low yield of product 4a after reported reaction times (Table 1, entries 2–7). The yield of the product 4a improved remarkably to 98% in the presence of new synthesized ionic liquid urazoliumdiacetate (Table 1, entry 8). The multicomponent reaction was attempted in various amounts of the catalyst and media (Table 1, entries 8–11). The results revealed that this multicomponent reaction lead to the product 4a in higher yield and shorter reaction time using 0.3 ml ionic liquid per 1 mmol substrate.

**Table 1:** The optimization of catalyst and reaction condition for the synthesis of 4a

Entry	Catalyst	Solvent/condition	Catalyst loading	Reaction time	Reaction yield (%)
1	—	—/reflux	—	24 h	—
2	HCl	EtOH/reflux	10 drops	14 h	36
3	K-10	EtOH/reflux	0.1 g	8 h	42
4	HY-zeolite	EtOH/reflux	0.1 g	8 h	45
5	Fe_3_O_4_	EtOH	0.05 g	6 h	52
6	[BMIM]Br	[BMIM]Br/heat, 80 °C	0.5 mL	2 h	65
7	[DBU]OAc	[DBu]OAc/heat, 80 °C	0.5 mL	2.5 h	61
8	Urazolium diAc	[Urazolium]diAc/heat, 80 °C	0.5 mL	20	98
9	Urazolium diAc	[Urazolium]diAc/heat, 80 °C	0.3 mL	20	98
10	Urazolium diAc	[Urazolium]diAc/heat, 80 °C	0.2 mL	35	94

To expand the scope and generality of the application of urazoliumdiacetate in this reaction, various benzaldehydes were reacted with 4-aminoazobenzene and thioglycolic acid. The results are summarized in Table 2. Electron-withdrawing groups on aldehydes showed increased yields in comparison to electron-releasing groups. It is because of the increasing electrophilic properties of carbon atom of carbonyl group of aldehyde bearing electron withdrawing moiety for nucleophilic addition reaction. As shown in Table 2, to control the efficiency of this method some pyrazole carbaldehydes were synthesized and undergo the multicomponent reaction with anilines and thioglycolic acid. The anilines with steric hindrance in substituents carried out the reaction in higher reaction time. Anilines with electron releasing moiety shorten the reaction time.

**Table 2:** The scope of multicomponent synthesis of 4a using urazoliumdiacetate

Entry	Product	Time (min)	Yield^a^^,^^b^ (%)	Mp (°C)
1	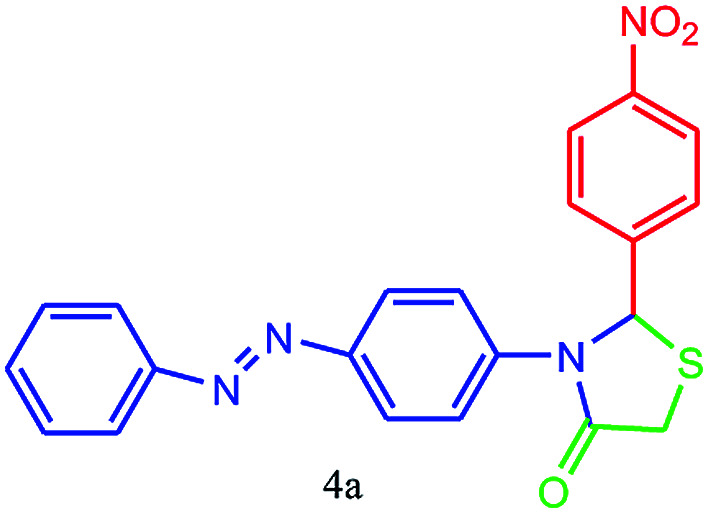	20	98	242–244
2	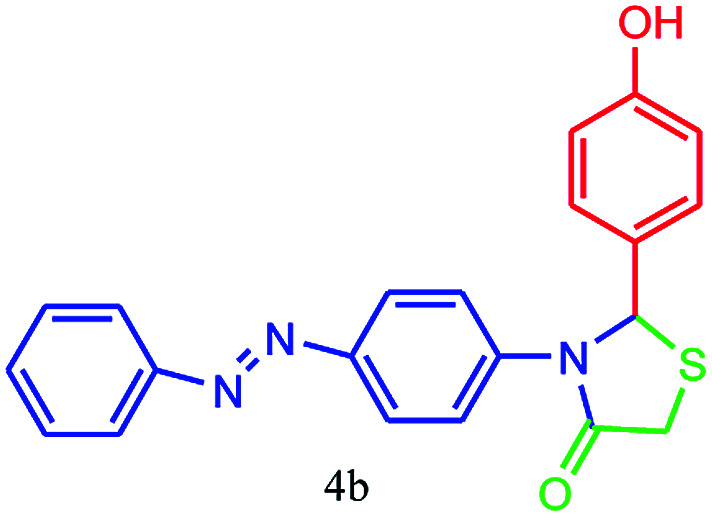	30	89	221–223
3	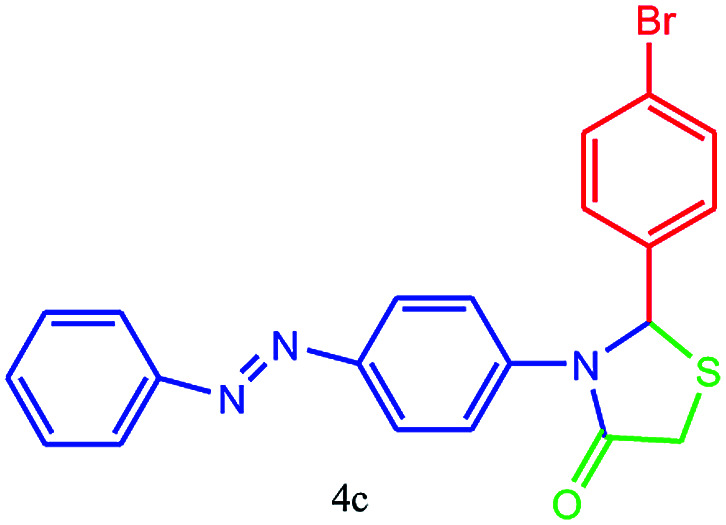	15	98	251–253
4	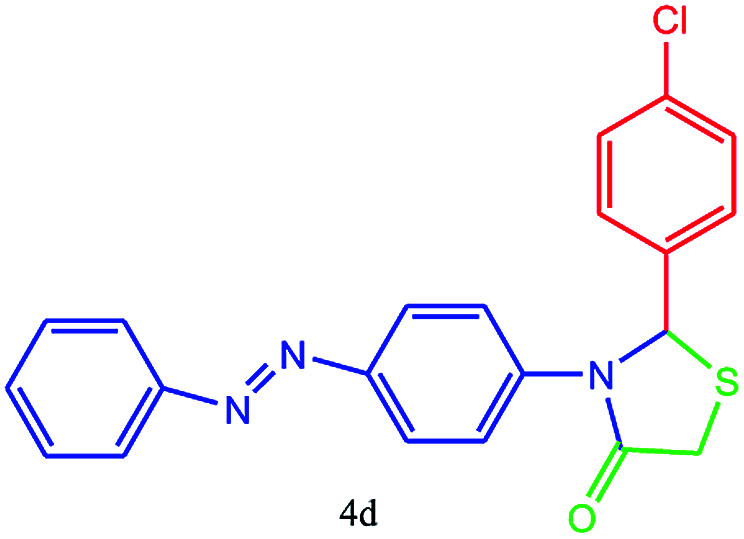	15	96	242–244
5	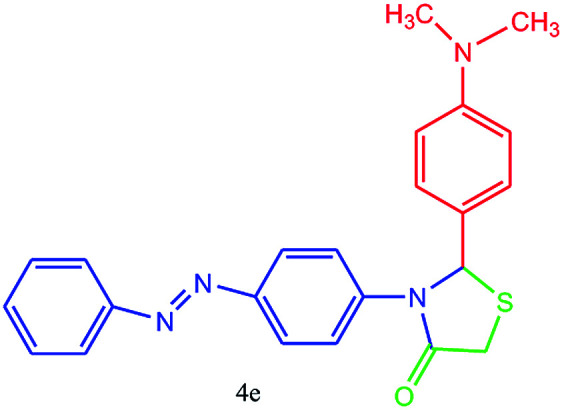	25	94	255–257
6	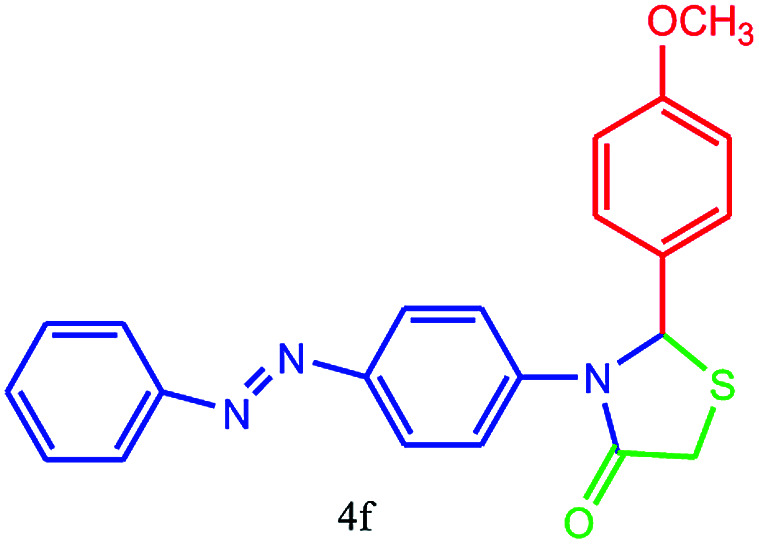	35	89	240–242
7	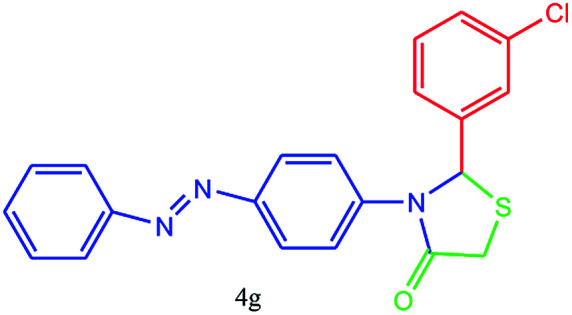	25	88	257–259
8	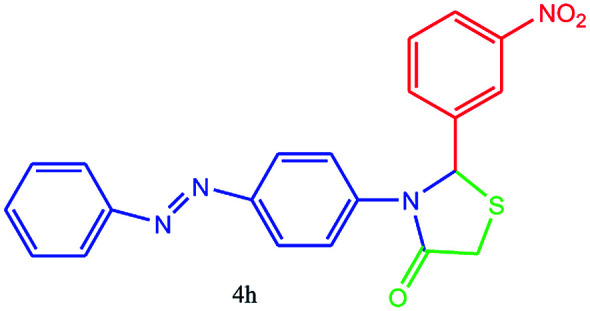	20	96	205–207
9	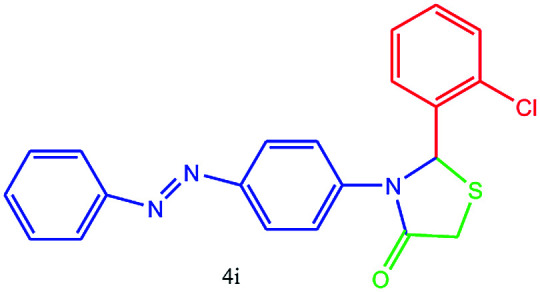	35	89	231–233
10	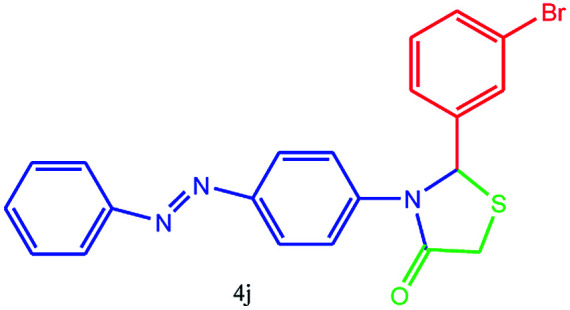	20	97	139–141
11	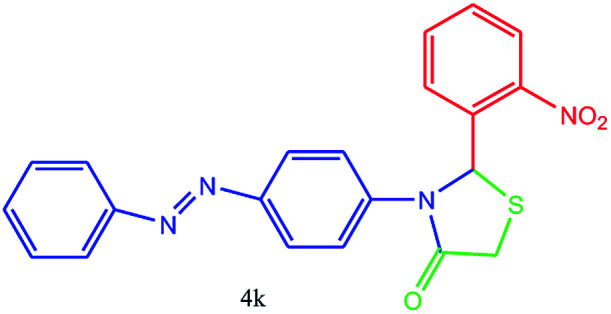	25	94	156–158
12	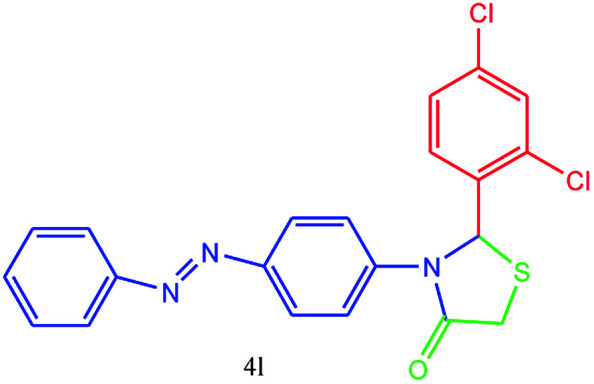	20	93	246–248
13	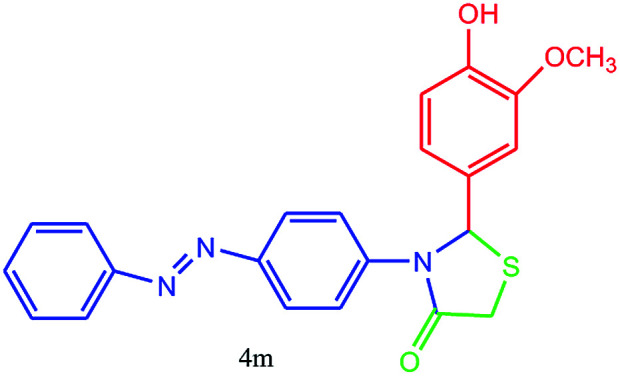	25	89	>300
14	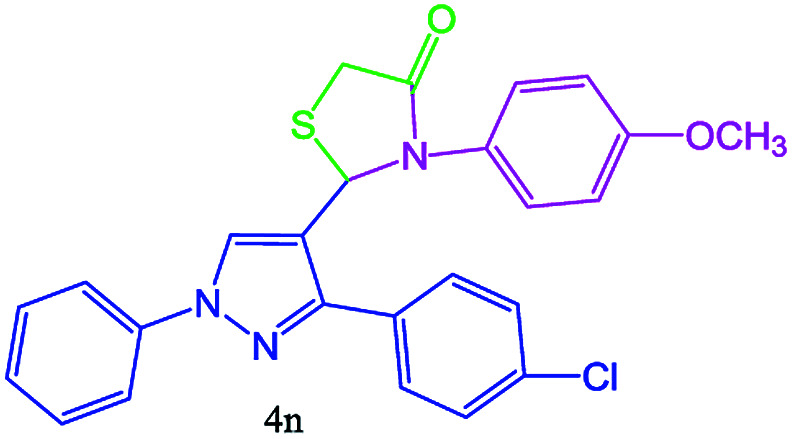	15	94	Oil
15	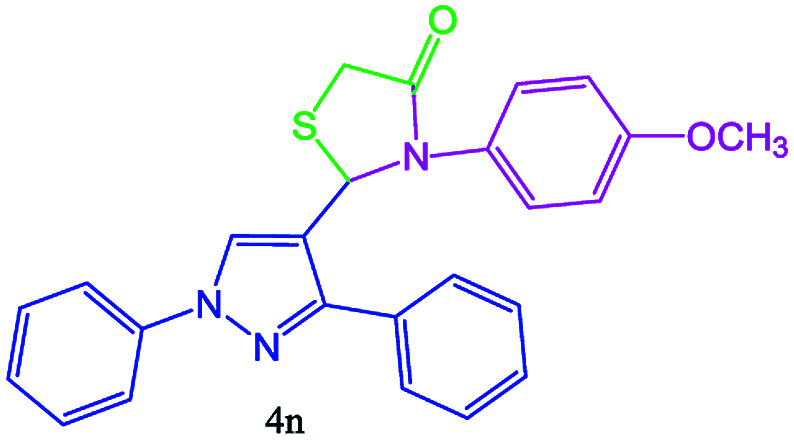	15	96	Oil
16	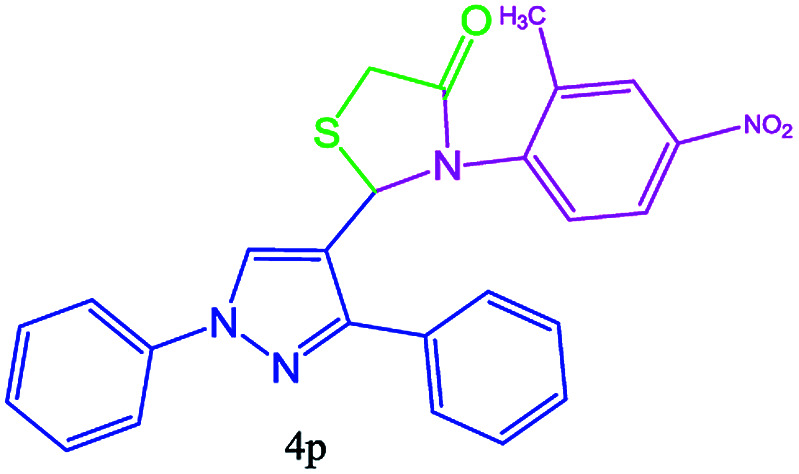	20	94	Oil
17	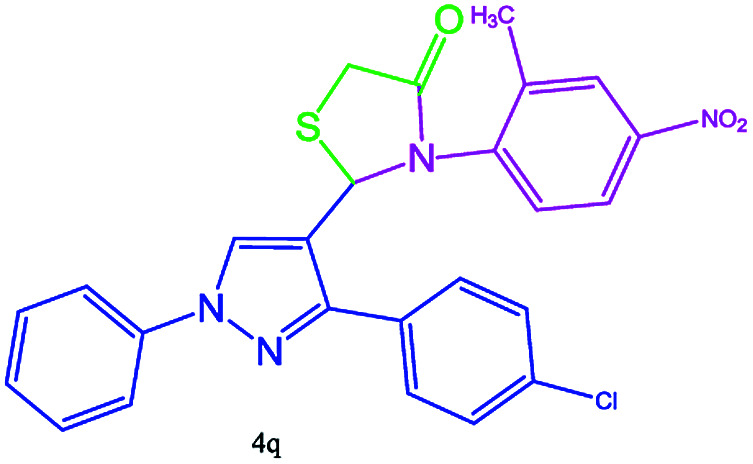	20	92	Oil

a Reaction conditions: reactions were performed with aldehyde (1 mmol), 4-aminoazobenzene (1 mmol) and thioglycolic acid (1 mmol) in urazolium diacetate catalyst (0.3 mL) under solvent-free condition at r. t. for required reaction time as identified by TLC.

b All of synthesized compounds are unknown and were characterized completely by various analysis.

All of new synthesized thiazolidin-4-ones were characterized by FT-IR, ^1^H NMR, ^13^C NMR and elemental analysis. Because of poor solubility in some cases (4a, 4e and 4j), unfortunately, taking ^13^C NMR was impossible, for this reason, we were interested in taking mass spectra instead of ^13^C NMR.

**Scheme 3:**
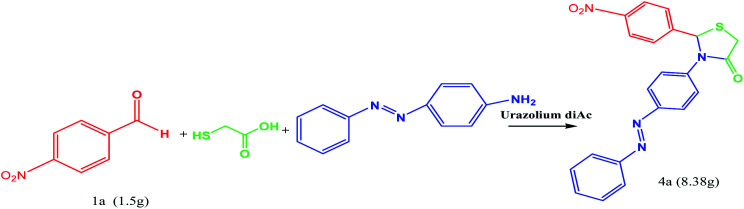
The gram scale analysis for the synthesis of product 4a.

In order to demonstrate the potential application of this route, the reaction was carried out on a gram scale. As shown in Scheme 3, when 1.5 g of 1a was used under the standard conditions, the product 4a was obtained in 98% yield with the reaction time prolonged to 25 min, indicating that this route could be scaled up to a preparing scale.

**Scheme 4:**
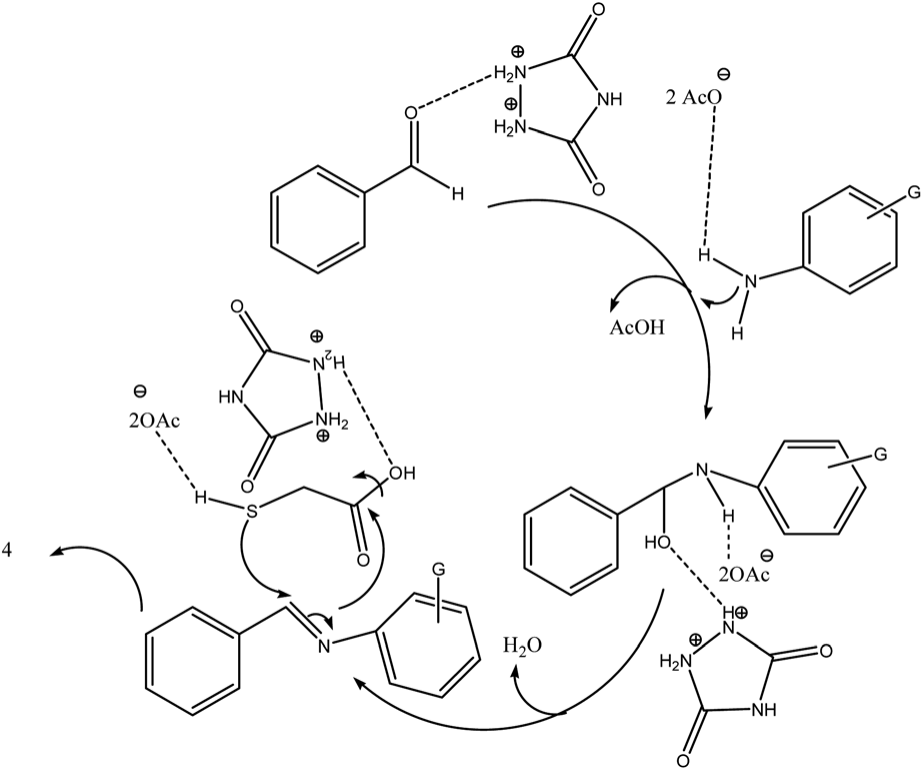
Proposed mechanism of thiazolidine-4-ones using urazoliumdiacetate.

As proposed in Scheme 4, initially, the carbonyl moiety of aldehyde and amino group was activated by urazoliumdiacetate by dipolarization to facilitate the nucleophilic attack of amine to carbonyl group. Then, the imine moiety was produced *via* elimination of H_2_O. Nucleophilic attack of activated sulphur to imine followed by intramolecular nucleophilic attack of secondary amine to carboxylic acid moiety lead to product 4. The mechanistic pathway was supported by literature.^30,53^

**Fig. 1:**
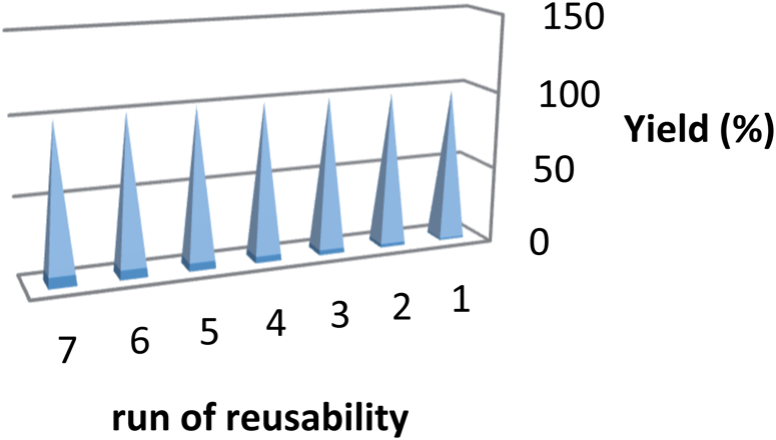
The reusability of catalyst.

After reaction, the ionic liquid is easily separated from the reaction medium by washing with distilled water (IL is soluble in water). The washed ionic liquid is distilled under vacuum to recover solvent for reuse in subsequent reactions. After seven successive runs, recycled ionic liquid showed suitable efficiency with regard to reaction time and yield (Fig. 1).

## Conclusion

In conclusion, we synthesize and apply urazolium diacetate as a novel Brønsted dicationic acid for the preparation of new azo dispersive dyes and pyrazolyl compounds with thiazolidine-4-one moiety. The notable benefits of this synthesis are: catalyst is inexpensive, non-toxic, easy handling, green and reusable. Simple work-up procedure, short reaction time and high yields of product, together with avoiding toxic and hazardous solvent are the other advantages. To the best of our knowledge, the ionic liquid urazolium diacetate and all of synthesized azo dispersive dyes and pyrazolyl compounds with thiazolidine-4-one moiety are completely new and this is the first report for the synthesis of thiazolidine-4-ones using ionic liquid urazolium diacetate.

## Experimental section

### Materials and methods

Chemicals were purchased from Merck and Fluka. All solvents used were dried and distilled according to standard procedures. Melting points were measured on an Electrothermal 9100 apparatus. IR spectra were determined on a Shimadzu FT-IR 8600 spectrophotometer. ^1^H and ^13^C NMR spectra were determined on a Bruker DRX Avance instrument at 500 or 300 and 125 or 75 MHz. HR-MS data were taken by Quadropole 5975C made by Agilent technologies.

### The procedure for the synthesis of ionic liquid urazoliumdiacetate

A mixture of 1 mmol of urazole and excess amount of acetic acid were heated at 80 °C for 4 hours to obtain a yellow-orange like oil. The corresponding oil was washed and decanted with diethylether (3 × 10 mL) to departure of the unreacted materials. The residue was dried under vacuum to reach pure ionic liquid. The ionic liquid was synthesized for the first time and characterized by FT-IR and ^1^H NMR. FT-IR (KBr, cm^−1^): 3270, 3020, 1787, 1467, and 1235. ^1^H NMR (DMSO-d_6_, 500 MHz): *δ*_H_; 2.33 (s, 6H), 10.65 (s, 4H) ppm.

### General procedure for the synthesis of 1,3-thiazolidine-4-ones

A mixture of arylaldehyde (1 mmol), 4-aminoazobenzene (1 mmol), thioglycolic acid and urazolium diacetate (0.3 mmol) was heated at 80 °C for the required reaction times as indicated in Table 2. After completion of the reaction, as indicated by TLC, the reaction mixture was dissolved in 20 mL of H_2_O. The product was separated by filtration and recrystallized from EtOH and dried to afford crystalline compounds of 4a–m. Then, the ionic liquid was recovered for subsequent use. All of synthesized compounds were characterized by their physical constant, IR, NMR, mass spectroscopy and elemental analysis.

### Analytical data for selected compounds

#### 2-(4-Nitrophenyl)-3-(4-(phenyldiazenyl)phenyl)thiazolidin-4-one (4a)

Mp 242–244 °C, FT-IR (KBr, cm^−1^): 1630 (C=O stretching), 1590 (aromatic C=C stretching or N=N stretching), 1387 (NO_2_ symmetric stretching, C–N or C–S stretching) cm^−1^. ^1^H NMR (DMSO-d_6_, 500 MHz): *δ*_H_; 4.42 (s, 2H), 6.09 (s, 1H), 6.67 (d, *J* = 8.8 Hz, 2H), 7.41 (t, *J* = 7.3 Hz, 1H), 7.50 (t, *J* = 8.0 Hz, 2H), 7.56–7.65 (m, 2H), 7.65 (d, *J* = 8.8 Hz, 2H), 7.73 (d, *J* = 8.5 Hz, 2H), 7.87–7.92 (m, 2H) ppm. HR-MS (*m*/*z* 404). Anal. calcd for C_21_H_16_N_4_O_3_S: C, 62.36; H, 3.99; N, 13.85. Found: C, 62.39; H, 3.98; N, 13.89.

#### 2-(4-Hydroxyphenyl)-3-(4-(phenyldiazenyl)phenyl)thiazolidin-4-one (4b)

Mp 221–223 °C, FT-IR (KBr, cm^−1^): 1649 (C=O stretching), 1594 (aromatic C=C or N=N stretching), 1450 (aromatic C=C stretching), 1400, 1370 (C–N, C–S or C–O stretching) cm^−1^. ^1^H NMR (DMSO-d_6_, 500 MHz): *δ*_H_; 4.31 (s, 2H), 6.10 (t, *J* = 8.8 Hz, 3H), 6.67 (d, *J* = 8.8 Hz, 2H), 7.28 (d, *J* = 8.5 Hz, 2H), 7.41 (t, *J* = 6.1 Hz, 1H), 7.50 (t, *J* = 7.3 Hz, 2H), 7.66 (d, *J* = 8.8 Hz, 2H), 7.73 (d, *J* = 8.4 Hz, 2H), 9.13 (s, 1H, OH) ppm. ^13^C NMR (DMSO-d_6_, 125 MHz): *δ*_C_; 62.5 (two peaks), 113.49, 118.78, 121.09, 121.78, 125.25, 127.22, 129.12, 129.28, 129.48, 132.56, 152.52, 152.96, 187.66 ppm. HR-MS (*m*/*z* 375). Anal. calcd for C_21_H_17_N_3_O_2_S: C, 67.18; H, 4.56; N, 11.19. Found: C, 67.21; H, 4.56; N, 11.17.

#### 2-(4-Bromophenyl)-3-(4-(phenyldiazenyl)phenyl)thiazolidin-4-one (4c)

Mp 251–253 °C, FT-IR (KBr, cm^−1^): 1630 (C=O stretching), 1598 (aromatic C=C stretching or N=N stretching), 1391 (C=N or C–S stretching), 1070 (C–Br stretching) cm^−1^. ^1^H NMR (DMSO-d_6_, 500 MHz): *δ*_H_; 4.45 (s, 2H), 6.09 (s, 1H), 6.67 (d, *J* = 8.8 Hz, 1H), 7.27 (d, *J* = 8.5 Hz, 1H), 7.41 (t, *J* = 7.3 Hz, 1H), 7.50 (t, *J* = 8.3 Hz, 4H), 7.54–7.60 (m, 1H), 7.65 (d, *J* = 8.7 Hz, 2H), 7.73 (d, *J* = 9.2 Hz, 2H), 7.87 (d, *J* = 7.4 Hz, 1H) ppm. ^13^C NMR (DMSO-d_6_, 75 MHz): *δ*_C_; 62.58 (two peaks), 122.93, 122.97, 123.50, 124.35, 125.63, 129.05, 129.94, 131.22, 131.74, 132.47, 149.30, 152.49, 173.64 ppm. HR-MS (*m*/*z* 437). Anal. calcd for C_21_H_16_BrN_3_OS: C, 57.54; H, 3.68; N, 9.59. Found: C, 57.55; H, 3.71; N, 9.57.

#### 2-(4-Chlorophenyl)-3-(4-(phenyldiazenyl)phenyl)thiazolidin-4-one (4d)

Mp 242–244 °C, FT-IR (KBr, cm^−1^): 1645 (C=O stretching), 1600 (aromatic C=C, N=N streching), 1421 (aromatic C=C stretching), 1340 (C–N or C–S stretching), 1146 (C–Cl stretching) cm^−1^. ^1^H NMR (DMSO-d_6_, 500 MHz): *δ*_H_; 4.47 (s, 2H), 6.09 (s, 1H), 6.67 (d, *J* = 8.9 Hz, 3H), 7.42 (d, *J* = 7.0 Hz, 1H), 7.50 (t, *J* = 7.9 Hz, 3H), 7.66 (d, *J* = 8.8 Hz, 3H), 7.73 (d, *J* = 7.3 Hz, 3H) ppm. ^13^C NMR (DMSO-d_6_, 75 MHz): *δ*_C_; 59.51, 60.23, 113.89, 116.08, 122.17, 123.67, 125.63, 128.42, 129.89, 141.23, 142.03, 143.27, 149.63, 153.32, 170.31 ppm. HR-MS (*m*/*z* 393). Anal. calcd for C_21_H_16_ClN_3_OS: C, 64.03; H, 4.09; N, 10.67. Found: C, 64.06; H, 4.07; N, 10.68.

#### 2-(4-(Dimethylamino)phenyl)-3-(4-(phenyldiazenyl)phenyl)thiazolidin-4-one (4e)

Mp 255–257 °C, FT-IR (KBr, cm^−1^): 1645 (C=O stretching), 1598 (aromatic C=C or N=N streching), 1450 (aromatic C=C stretching), 1375 (C–N or C–S stretching) cm^−1^. ^1^H NMR (DMSO-d_6_, 500 MHz): *δ*_H_; 3.00 (s, 6H, 2 CH3–N), 4.49 (s, 2H), 6.10 (s, 1H), 6.67 (d, *J* = 8.2 Hz, 2H), 6.78 (d, *J* = 8.5 Hz, 1H), 7.40 (d, *J* = 8.7 Hz, 2H), 7.50 (t, *J* = 7.9 Hz, 3H), 7.67 (t, *J* = 10.2 Hz, 3H), 7.74 (dd, *J* = 7.2 Hz, *J* = 1.3 Hz, 2H) ppm. HR-MS (*m*/*z* 402). Anal. calcd for C_23_H_22_N_4_OS: C, 68.63; H, 5.51; N, 13.92. Found: C, 68.66; H, 5.54; N, 13.94.

#### 2-(4-Methoxyphenyl)-3-(4-(phenyldiazenyl)phenyl)thiazolidin-4-one (4f)

Mp 240–242 °C, FT-IR (KBr, cm^−1^): 1610 (C=O stretching), 1586 (aromatic C=C or N=N stretching), 1500, 1404 (C–S or C–O stretching), 1300 (C–N stretching) cm^−1^. ^1^H NMR (DMSO-d_6_, 500 MHz): *δ*_H_; 3.73 (s, 3H, OCH_3_), 4.40 (s, 2H), 6.10 (s, 1H), 6.85 (d, *J* = 8.4 Hz, 2H), 7.00 (d, *J* = 6.0 Hz, 1H), 7.09–7.13 (m, 2H), 7.29 (d, *J* = 8.4 Hz, 2H), 7.42 (d, *J* = 8.3 Hz, 1H), 7.54–7.67 (m, 2H), 7.74 (d, *J* = 8.1 Hz, 1H), 7.88 (t, *J* = 9.4 Hz, 1H), 7.94 (t, *J* = 9.6 Hz, 1H) ppm. ^13^C NMR (DMSO-d6, 125 MHz): *δ*_C_; 50.89, 55.40, 55.51, 122.48, 123.03, 123.59, 123.89, 127.93, 129.48, 129.67, 130.23, 130.34, 130.89, 131.85, 147.89, 191.37 ppm. HR-MS (*m*/*z* 389). Anal. calcd for C_22_H_19_N_3_O_2_S: C, 67.84; H, 4.92; N, 10.79. Found: C, 67.86; H, 4.94; N, 10.75.

#### 2-(3-Chlorophenyl)-3-(4-(phenyldiazenyl)phenyl)thiazolidin-4-one (4g)

Mp 231–233 °C, FT-IR (KBr, cm^−1^): *δ*_H_; 1631 (C=O stretching), 1594 (aromatic C=C stretching or N=N streching), 1450 (aromatic C=C stretching), 1330 (C–N or C–S stretching), 1150 (C–Cl stretching). ^1^H NMR (DMSO-d_6_, 500 MHz): *δ*_H_; 4.51 (s, 2H), 6.13 (s, 1H), 6.89 (dd, *J* = 3.5, 14.5 Hz, 3H), 7.31–7.45 (m, 2H), 7.52 (t, *J* = 11.5 Hz, 2H), 7.68 (dd, *J* = 3.0, 17.5 Hz, 2H), 7.75 (d, *J* = 8.5 Hz, 2H), 7.79–7.90 (m, 2H) ppm. ^13^C NMR (DMSO-d_6_, 75 MHz): *δ*_C_; 57.21, 59.63, 113.90, 115.63, 122.17, 122.88, 125.62, 128.01, 129.29, 129.61, 129.67, 129.89, 132.55, 143.26, 152.83, 153.32, 171.03 ppm. HR-MS (*m*/*z* 393). Anal. calcd for C_21_H_16_ClN_3_OS: C, 64.03; H, 4.09; N, 10.67. Found: C, 64.06; H, 4.07; N, 10.65.

#### 2-(3-Nitrophenyl)-3-(4-(phenyldiazenyl)phenyl)thiazolidin-4-one (4h)

Mp 205–207 °C, FT-IR (KBr, cm^−1^): 1641 (C=O stretching), 1600 (aromatic C=C stretching), 1580 (N=N stretching or NO_2_ asymmetric stretching), 1483 (C=C aromatic stretching), 1336 (C–N or C–S stretching or NO_2_ symmetric stretching), 1150 cm^−1^. ^1^H NMR (DMSO-d_6_, 500 MHz): *δ*_H_; 4.65 (s, 2H), 6.14 (s, 1H), 6.70 (d, *J* = 8.0 Hz, 3H), 7.33–7.45 (m, 1H), 7.52 (t, *J* = 11.0 Hz, 2H), 7.58 (t, *J* = 13.0 Hz, 1H), 7.68 (d, *J* = 9.0 Hz, 2H), 7.76 (d, *J* = 13.5 Hz, 3H), 7.87–7.92 (m, 1H) ppm. ^13^C NMR (DMSO-d_6_, 75 MHz): *δ*_C_; 55.83, 62.10, 113.92, 121.98, 122.17 (two peaks), 123.08, 125.63, 129.65, 133.43, 136.76, 143.29, 145.63, 148.24, 148.83, 153.33, 176.53 ppm. HR-MS (*m*/*z* 393). Anal. calcd for C_21_H_16_N_4_O_3_S: C, 62.36; H, 3.99; N, 13.85. Found: C, 62.39; H, 3.97; N, 13.86.

#### 2-(2-Chlorophenyl)-3-(4-(phenyldiazenyl)phenyl)thiazolidin-4-one (4i)

Mp 231–233 °C, FT-IR (KBr, cm^−1^): 1641 (C=O stretching), 1581 (aromatic C=C stretching or N=N stretching), 1446 (aromatic C=C stretching), 1235 (C–N or C–S stretching), 1173 (C–Cl stretching) cm^−1^. ^1^H NMR (DMSO-d_6_, 300 MHz): *δ*_H_; 4.58 (s, 2H), 6.12 (s, 1H), 6.70 (d, *J* = 8.7 Hz, 1H), 7.24–7.28 (m, 1H), 7.29–7.343 (m, 1H), 7.347–7.394 (m, 1H), 7.39–7.430 (m, 1H), 7.438–7.450 (m, 1H), 7.455–7.503 (m, 1H), 7.508–7.556 (m, 2H), 7.559–7.60 (m, 2H), 7.61–7.67 (m, 1H), 7.71 (d, *J* = 14.4 Hz, 1H) ppm. ^13^C NMR (DMSO-d_6_, 75 MHz): *δ*_C_; 60.69 (two peaks), 113.89, 122.18, 122.67, 122.91, 125.64, 127.48, 128.70, 129.06, 129.17, 129.90, 131.40, 140.03, 142.48, 153.69, 173.46 ppm. HR-MS (*m*/*z* 404). Anal. calcd for C_21_H_16_N_4_O_3_S: C, 62.36; H, 3.99; N, 13.85. Found: C, 62.38; H, 3.97; N, 13.88.

#### 2-(3-Bromophenyl)-3-(4-(phenyldiazenyl)phenyl)thiazolidin-4-one (4j)

Mp 139–141 °C, FT-IR (KBr, cm^−1^): 1625 (C=O stretching), 1590 (aromatic C=C or N=N stretching), 1383 (C–N or C–S or C–Br stretching) cm^−1^. ^1^H NMR (DMSO-d_6_, 500 MHz): *δ*_H_; 4.49 (s, 2H), 6.10 (s, 1H), 6.67 (d, *J* = 8.8 Hz, 1H), 7.26–7.31 (m, 2H), 7.39–7.42 (m, 1H), 7.50 (t, *J* = 7.4 Hz, 3H), 7.59–7.66 (m, 1H), 7.73 (d, *J* = 8.5 Hz, 1H), 7.76 (d, *J* = 5.1 Hz, 2H), 7.91 (d, *J* = 8.2 Hz, 1H), 7.98 (d, *J* = 6.8 Hz, 1H) ppm. HR-MS (*m*/*z* 439). Anal. calcd for C_21_H_16_N_3_SOBr: C, 57.54; H, 3.68; N, 9.59. Found: C, 57.57; H, 3.67; N, 9.57.

#### 2-(2-Nitrophenyl)-3-(4-(phenyldiazenyl)phenyl)thiazolidin-4-one (4k)

Mp 156–158 °C, FT-IR (KBr, cm^−1^): 1632 (C=O stretching), 1600 (aromatic C=C or N=N stretching), 1550 (NO_2_ asymmetric stretching), 1518 (aromatic C=C stretching), 1391 (NO2 symmetric stretching) cm^−1^. ^1^H NMR (DMSO-d_6_, 500 MHz): *δ*_H_; 3.53 (s, 2H), 6.10 (s, 1H), 6.67 (s, 2H), 7.14 (t, *J* = 8.5 Hz, 1H), 7.35 (t, *J* = 8.4 Hz, 1H), 7.40 (t, *J* = 7.3 Hz, 1H), 7.50 (t, *J* = 8.0 Hz, 2H), 7.57–7.59 (m, 1H), 7.65 (d, *J* = 8.7 Hz, 2H), 7.73 (d, *J* = 9.3 Hz, 2H), 7.88 (d, *J* = 7.5 Hz, 1H) ppm. ^13^C NMR (DMSO-d_6_, 125 MHz): *δ*_C_; 54.59, 55.10, 113.45 (two peaks), 121.75, 122.50, 123.99, 125.21, 129.24 (two peaks), 129.45, 141.09, 142.84, 147.16, 152.50, 152.93, 168.41 ppm. HR-MS (*m*/*z* 393). Anal. calcd for C_21_H_16_N_4_O_3_S: C, 62.36; H, 3.99; N, 13.85. Found: C, 62.35; H, 3.95; N, 13.89.

#### 2-(2,4-Dichlorophenyl)-3-(4-(phenyldiazenyl)phenyl)thiazolidin-4-one (4l)

Mp 246–248 °C, FT-IR (KBr, cm^−1^): 1645 (C=O stretching), 1581 (aromatic C=C stretching or N=N stretching), 1446 (aromatic C=C stretching), 1350 (C–N or C–S stretching), 1141, 1091 (C–Cl stretching). ^1^H NMR (DMSO-d_6_, 500 MHz): *δ*_H_; 4.54 (s, 2H), 6.13 (s, 1H), 7.39–7.46 (m, 2H), 7.50–7.55 (m, 1H), 7.56–7.65 (m, 2H), 7.66–7.73 (m, 1H), 7.74–7.78 (m, 2H), 7.83 (d, *J* = 6.5 Hz, 1H), 7.88–7.94 (m, 2H), 7.99 (d, *J* = 14.5 Hz, 1H) ppm. ^13^C NMR (DMSO-d_6_, 75 MHz): *δ*_C_; 60.24, 61.33, 122.75, 122.92, 123.01, 123.49, 124.36, 125.91, 127.66, 128.60, 128.67, 129.36, 129.85, 132.27, 139.23, 152.48, 179.64 ppm. HR-MS (*m*/*z* 427). Anal. calcd for C_21_H_15_Cl_2_N_3_OS: C, 58.89; H, 3.53; N, 9.81. Found: C, 58.85; H, 3.56; N, 9.82.

#### 2-(4-Hydroxy-3-methoxyphenyl)-3-(4-(phenyldiazenyl)phenyl)thiazolidin-4-one (4m)

Mp > 300 °C, FT-IR (KBr, cm^−1^): 3206 (O–H stretching), 1632 (C=O stretching), 1600 (aromatic C=C stretching or N=N stretching), 1450 (aromatic C=C stretching), 1400 (C–N or C–S stretching), 1235 (C–O stretching). ^1^H NMR (DMSO-d_6_, 300 MHz): *δ*_H_; 3.69 (s, 3H, OCH_3_), 4.41 (s, 2H), 6.15 (s, 2H), 7.42 (d, *J* = 5.1 Hz, 1H), 7.49–7.56 (m, 2H), 7.60 (d, *J* = 4.5 Hz, 1H), 7.68 (d, *J* = 2.7 Hz, 2H), 7.75 (d, *J* = 7.2 Hz, 2H), 7.88–7.95 (m, 2H) ppm. ^13^C NMR (DMSO-d_6_, 75 MHz): *δ*_C_; 55.12, 60.13, 67.82 (OCH_3_), 113.91, 117.43, 119.94, 122.17, 122.92, 123.03, 125.08, 129.67, 129.88, 143.25, 151.63, 152.42, 152.89, 153.36, 176.53 ppm. HR-MS (*m*/*z* 405). Anal. calcd for C_22_H_19_N_3_O_3_S: C, 65.17; H, 4.72; N, 10.36. Found: C, 65.18; H, 4.76; N, 10.34.

#### 2-(3-(4-Chlorophenyl)-1-phenyl-1H-pyrazol-4-yl)-3-(4-methoxyphenyl) thiazolidin-4-one (4n)

Oil, FT-IR (KBr, cm^−1^): 3126 (C–H aromatic), 2981 (C–H aliphatic), 1731 (C=O stretch), 1600, 1541, 1502, 1342, 1156. ^1^H NMR (DMSO-d_6_, 500 MHz): *δ*_H_; 3.28 (d, *J* = 15.3 Hz, 1H), 3.5 (d, *J* = 15.3 Hz, 1H), 3.52 (s, 3H, CH_3_O), 5.48 (s, 1H), 7.32 (t, *J* = 7.4 Hz, 1H), 7.43–7.49 (m, 4H), 7.73–7.80 (m, 4H), 8.22 (s, 1H) ppm. ^13^C NMR (DMSO-d_6_, 75 MHz): *δ*_C_; 32.9, 40.4, 60.5, 118.0, 125.5, 127.4, 127.8, 128.4, 128.7, 129.5, 129.8, 133.6, 138.8, 143.1, 168.3 (C=O) ppm.

#### 3-(4-Methoxyphenyl)-2-(1,3-diphenyl-1H-pyrazol-4-yl)thiazolidin-4-one (4o)

Oil, FT-IR (KBr, cm^−1^): 3058 (C–H aromatic), 2977 (C–H aliphatic), 1737 (C=O stretch), 1596 (C=C, aromatic), 1541, 1500, 1288 (C–O). ^1^H NMR (DMSO-d_6_, 500 MHz): *δ*_H_; 3.29 (d, *J* = 15.2 Hz, 1H), 3.49 (d, *J* = 15.2 Hz, 1H), 3.58 (s, 3H, CH3O), 5.49 (s, 1H), 7.30 (td, *J* = 7.9 Hz, 0.7 Hz, 2H), 7.37–7.41 (m, 2H), 7.45–7.48 (m, 6H), 7.76 (dd, *J* = 8.4, 0.6 Hz, 2H), 7.8 (d, *J* = 8.4 Hz, 2H), 8.09 (s, 1H). ^13^C NMR (DMSO-d_6_, 75 MHz): *δ*_C_; 32.9, 43.5, 60.5, 118.0, 125.7, 127.1, 127.3, 127.4, 127.5, 127.6, 128.4, 131.3, 138.6, 150.0, 168.8 (C=O) ppm.

#### 3-(2-Methyl-4-nitrophenyl)-2-(1,3-diphenyl-1H-pyrazol-4-yl)thiazolidin-4-one (4p)

Oil, FT-IR (KBr, cm^−1^): 3126 (C–H aromatic), 2981 (C–H aliphatic), 1730 (C=O stretch), 1596 (C=C aromatic), 1541 (NO_2_), 1500, 1450, 1363. ^1^H NMR (DMSO-d_6_, 500 MHz): *δ*_H_; 3.30 (d, *J* = 15.15 Hz, 1H), 3.50 (d, *J* = 15.15 Hz, 1H), 3.58 (s, 3H, CH_3_), 5.50 (s, 1H), 7.31 (t, *J* = 7.4 Hz, 1H), 7.40–7.42 (m, 1H), 7.45–7.49 (m, 7H), 7.75–7.78 (m, 2H), 7.80–7.82 (m, 2H), 8.23 (s, 1H). ^13^C NMR (DMSO-d_6_, 75 MHz): *δ*_C_; 30.9, 40.4, 43.5, 60.5, 60.6, 118.0, 125.7, 127.1, 127.3, 127.4, 127.5, 127.6, 128.4, 131.3, 138.6, 150.0, 168.8 (C=O) ppm.

#### 2-(3-(4-Chlorophenyl)-1-phenyl-1H-pyrazol-4-yl)-3-(2-methyl-4-nitrophenyl)thiazolidin-4-one (4q)

Oil, FT-IR (KBr, cm^−1^): 3128 (C–H aromatic), 2981 (C–H aliphatic), 1731 (C=O stretch), 1598 (C=C aromatic), 1539 (NO_2_), 1500, 1454, 1400 cm^−1^. ^1^H NMR (DMSO-d_6_, 300 MHz): *δ*_H_; 3.24 (d, *J* = 15.2 Hz 1H), 3.49 (d, *J* = 15.2 Hz 1H), 3.55 (s, 1H, CH_3_), 5.45 (s, 1H),7.26–7.30 (m, 2H),7.40–7.45 (m, 6H), 7.71–7.73 (m, 2H), 7.75–7.78 (m, 2H), 8.20 (s, 1H) ppm. ^13^C NMR (DMSO-d_6_, 75 MHz): *δ*_C_; 32.7, 40.3, 43.2, 60.4, 60.5, 117.8, 117.9, 125.7, 127.3, 127.6, 128.3, 128.6, 129.7, 133.1, 138.4, 148.7, 168.1 (C=O) ppm.

## Conflicts of interest

There are no conflicts to declare.

## Supplementary Material

RA-010-C9RA08649H-s001
