# Supplementary material for: Urazolium diacetate as a new, efficient and reusable Brønsted acid ionic liquid for the synthesis of novel derivatives of thiazolidine-4-ones
Source: RSC Adv. 2020 Jan 2;10(1):556–64. doi: 10.1039/c9ra08649h (PMC9047527; doi:10.1039/c9ra08649h)
Supplement: RA-010-C9RA08649H-s001 [file RA-010-C9RA08649H-s001.pdf]

Electronic Supplementary Material (ESI) for RSC Advances.
This journal is © The Royal Society of Chemistry 2019

Starting material	product	condition	Ref.
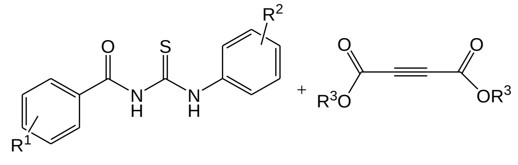	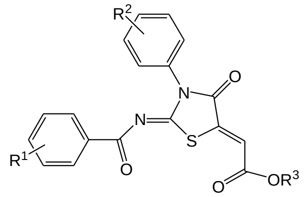	MW	[51]
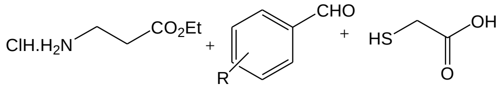	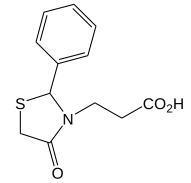	DIPEA, reflux in toluene	[51]
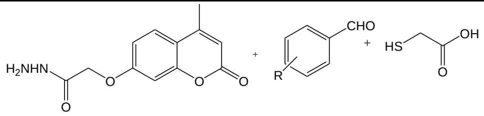	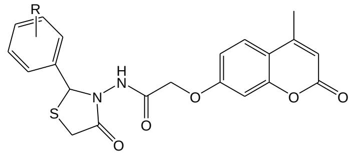	ZnCl2, dioxane, reflux	[52]
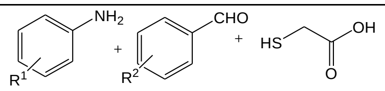	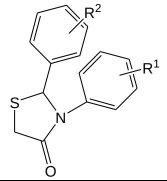	MW	[51]
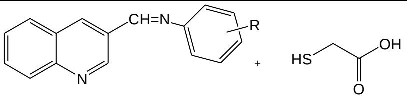	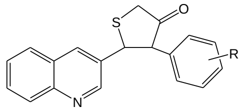	MW, zeolite	[51]

٤-٢- تهیه ی ترکیب ٢-(٤-نیترو فنیل)-٣-(٤-فنیل دیازنیل) فنیل) تیازولیدین-٤-اون (٤a)

Parameter	Value
1 Data File Name	D:/ NMR/ 1398/ 98-04/ 98-04-09/ Masomeh-Abdollahi/ BAYO-H.fid/ fid
2 Title	BAYO-H
3 Comment	
4 Origin	Varian
5 Owner	
6 Site	
7 Instrument	inova
8 Author	
9 Solvent	dmso
10 Temperature	25.0
11 Pulse Sequence	s2pul
12 Experiment	1D
13 Probe	ID
14 Number of Scans	32
15 Receiver Gain	30
16 Relaxation Delay	1.0000
17 Pulse Width	5.0000
18 Presaturation Frequency	
19 Acquisition Time	1.6396
20 Acquisition Date	2019-06-30T15:49:31
21 Modification Date	2019-06-30T15:51:18
22 Class	
23 Spectrometer Frequency	499.65
24 Spectral Width	9992.5
25 Lowest Frequency	-999.1
26 Nucleus	1H
27 Acquired Size	16384
28 Spectral Size	65536

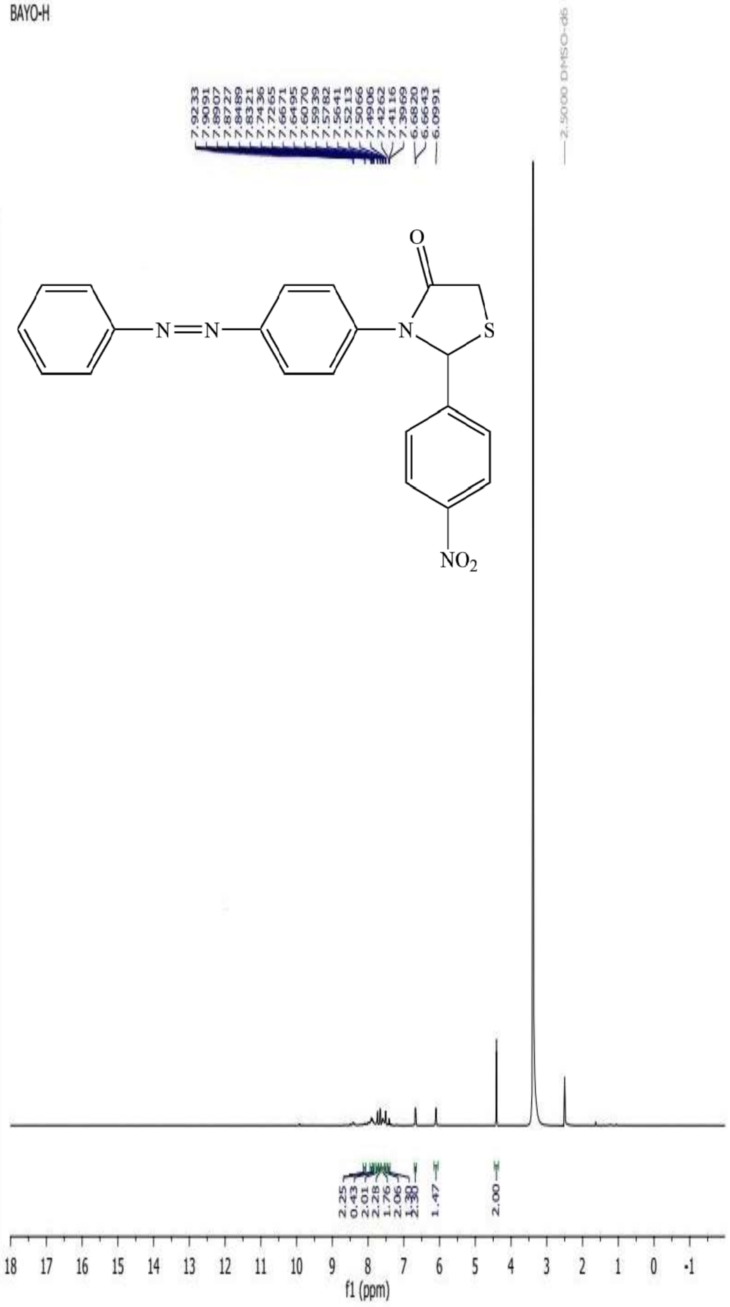

٤-٣- تهیه ی ترکیب ٢-(٤-نیترو فنیل)-٣-(٤-فنیل دیازنیل) فنیل) تیازولیدین-٤-اون (٤a)

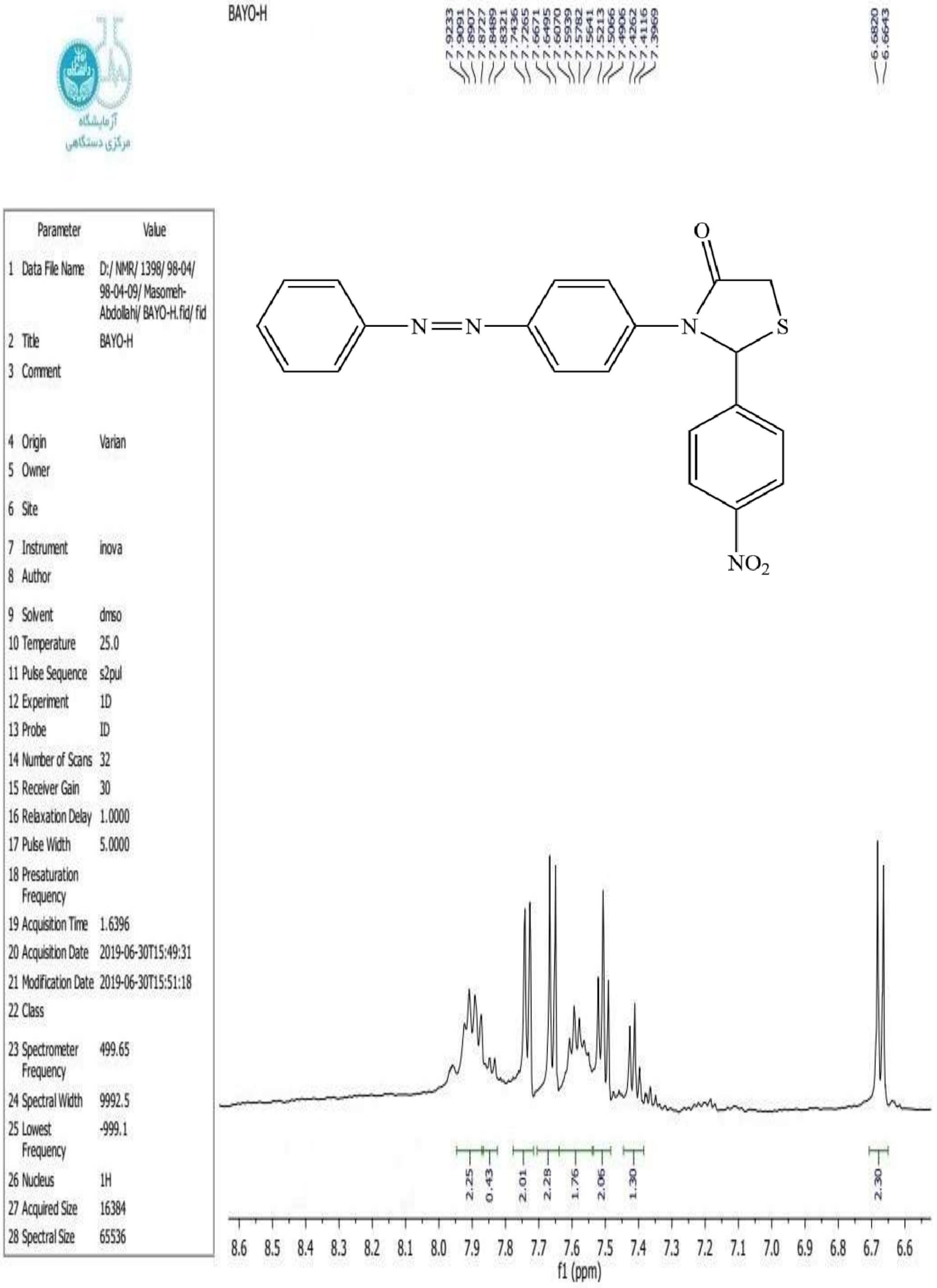

	Parameter	Value
1	Data File Name	D:/ NMR/ 1398/ 98-04/ 98-04-09/ Masomeh-Abdollahi/ BAYO-H.fid/ fid
2	Title	BAYO-H
3	Comment	
4	Origin	Varian
5	Owner	
6	Site	
7	Instrument	inova
8	Author	
9	Solvent	dmso
10	Temperature	25.0
11	Pulse Sequence	s2pul
12	Experiment	1D
13	Probe	ID
14	Number of Scans	32
15	Receiver Gain	30
16	Relaxation Delay	1.0000
17	Pulse Width	5.0000
18	Presaturation Frequency	
19	Acquisition Time	1.6396
20	Acquisition Date	2019-06-30T15:49:31
21	Modification Date	2019-06-30T15:51:18
22	Class	
23	Spectrometer Frequency	499.65
24	Spectral Width	9992.5
25	Lowest Frequency	-999.1
26	Nucleus	1H
27	Acquired Size	16384
28	Spectral Size	65536

٤-٤- تهیه ی ترکیب ۲-(٤-نیترو فنیل)-۳-(٤-فنیل دیازنیل) فنیل) تیازولیدین-٤-اون (٤a)

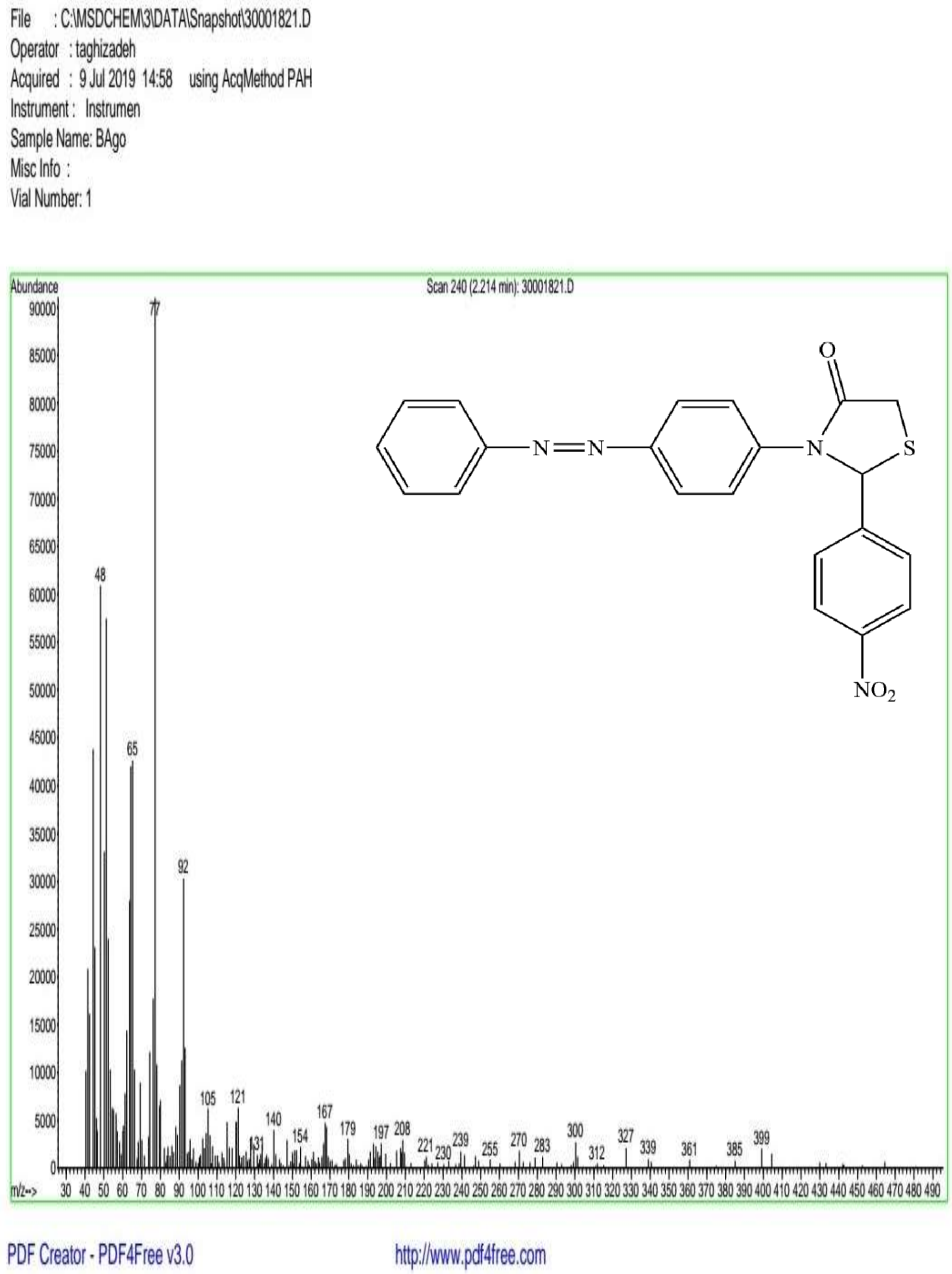

٤-٥- تهیه ی ترکیب ۲- (٤-هیدروکسی فنیل)- ۳ -(٤- (فنیل دیازنیل) فنیل) تیازولیدین- ٤-اون (٤b)

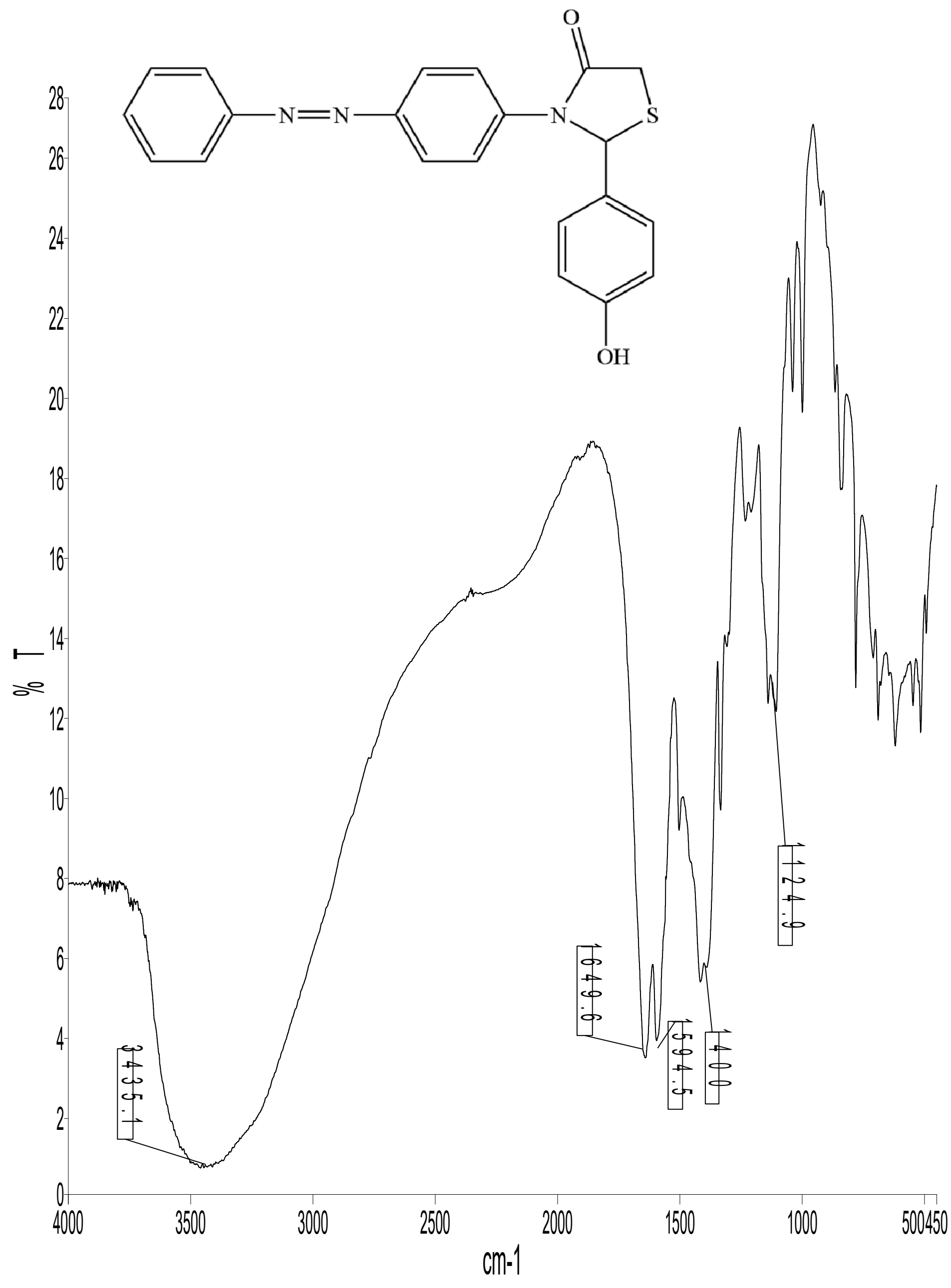

٤-٦- تهیه ی ترکیب ۲- (٤-هیدروکسی فنیل)- ۳ -(٤- (فنیل دیازنیل) فنیل) تیازولیدین- ٤-اون (4b)

Parameter	Value
1 Data File Name	D:/ NMR/ 1398/ 98-02/ 98-02-31/ 98-02-31/ masoome-abdolahi/ DY6-H.fid/ fid
2 Title	DY6-H
3 Comment	
4 Origin	Varian
5 Owner	
6 Site	
7 Instrument	inova
8 Author	
9 Solvent	dmso
10 Temperature	25.0
11 Pulse Sequence	s2pul
12 Experiment	1D
13 Probe	ID
14 Number of Scans	32
15 Receiver Gain	30
16 Relaxation Delay	1.0000
17 Pulse Width	5.0000
18 Presaturation Frequency	
19 Acquisition Time	2.0494
20 Acquisition Date	2019-05-21T13:35:19
21 Modification Date	2019-05-21T13:35:38
22 Class	
23 Spectrometer Frequency	499.67
24 Spectral Width	7994.4
25 Lowest Frequency	-999.2
26 Nucleus	1H
27 Acquired Size	16384
28 Spectral Size	65536

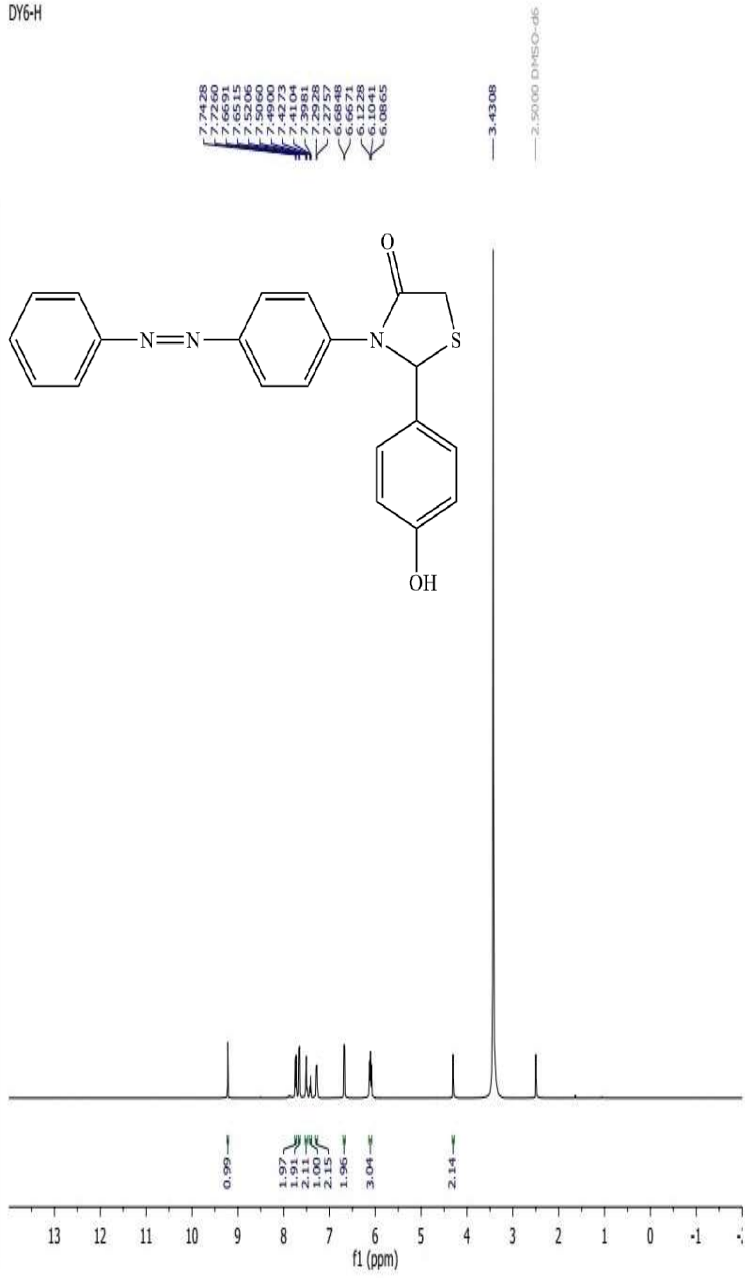

٤-٧- تهیه ی ترکیب ٢- (٤-هیدروکسی فنیل)- ٣ -(٤- (فنیل دیازنیل) فنیل) تیازولیدین- ٤-اون (4b)

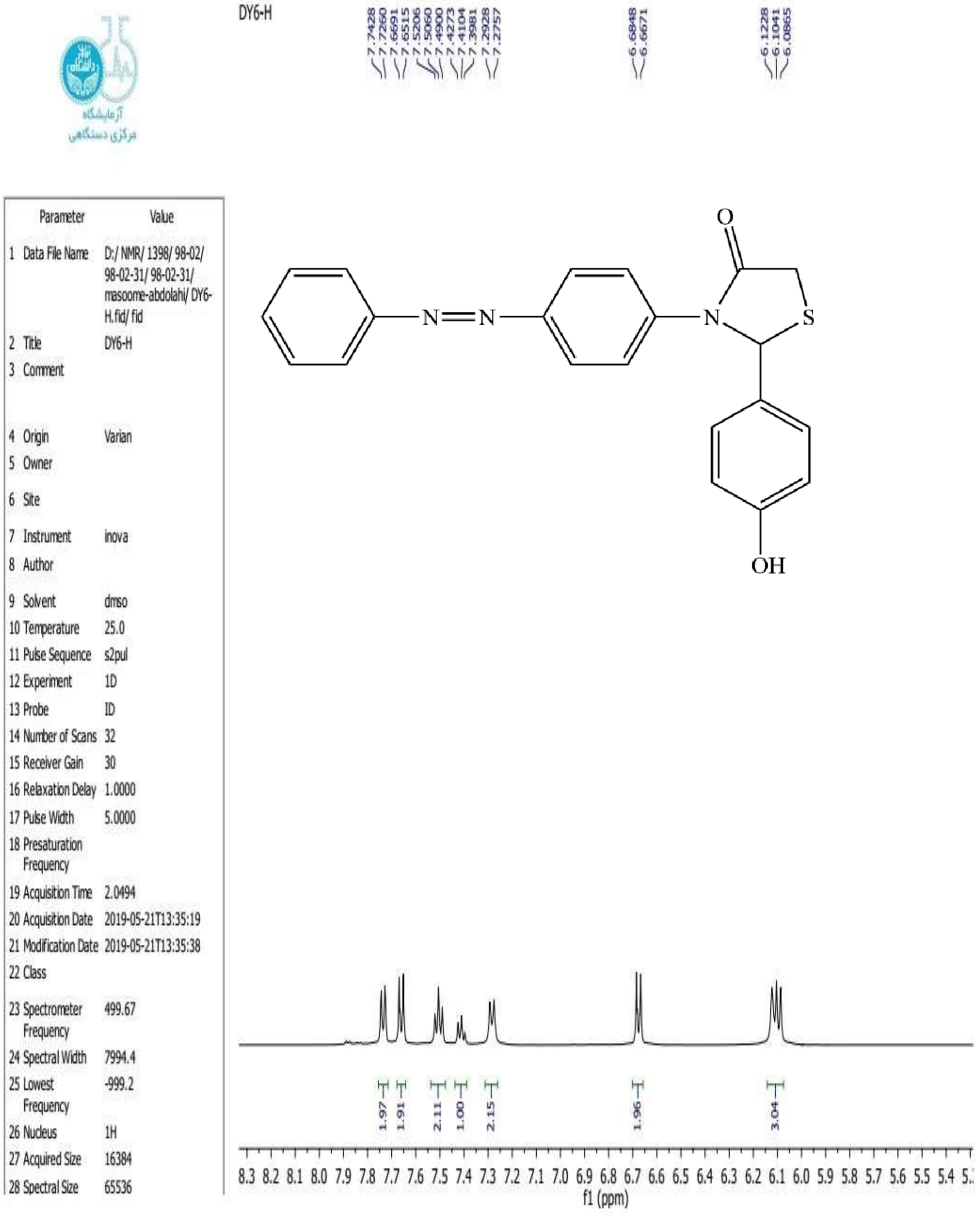

٤-٨- تهیه ی ترکیب ۲- (٤-هیدروکسی فنیل)- ۳ -(٤- (فنیل دیازنیل) فنیل) تیازولیدین- ٤-اون (4b)

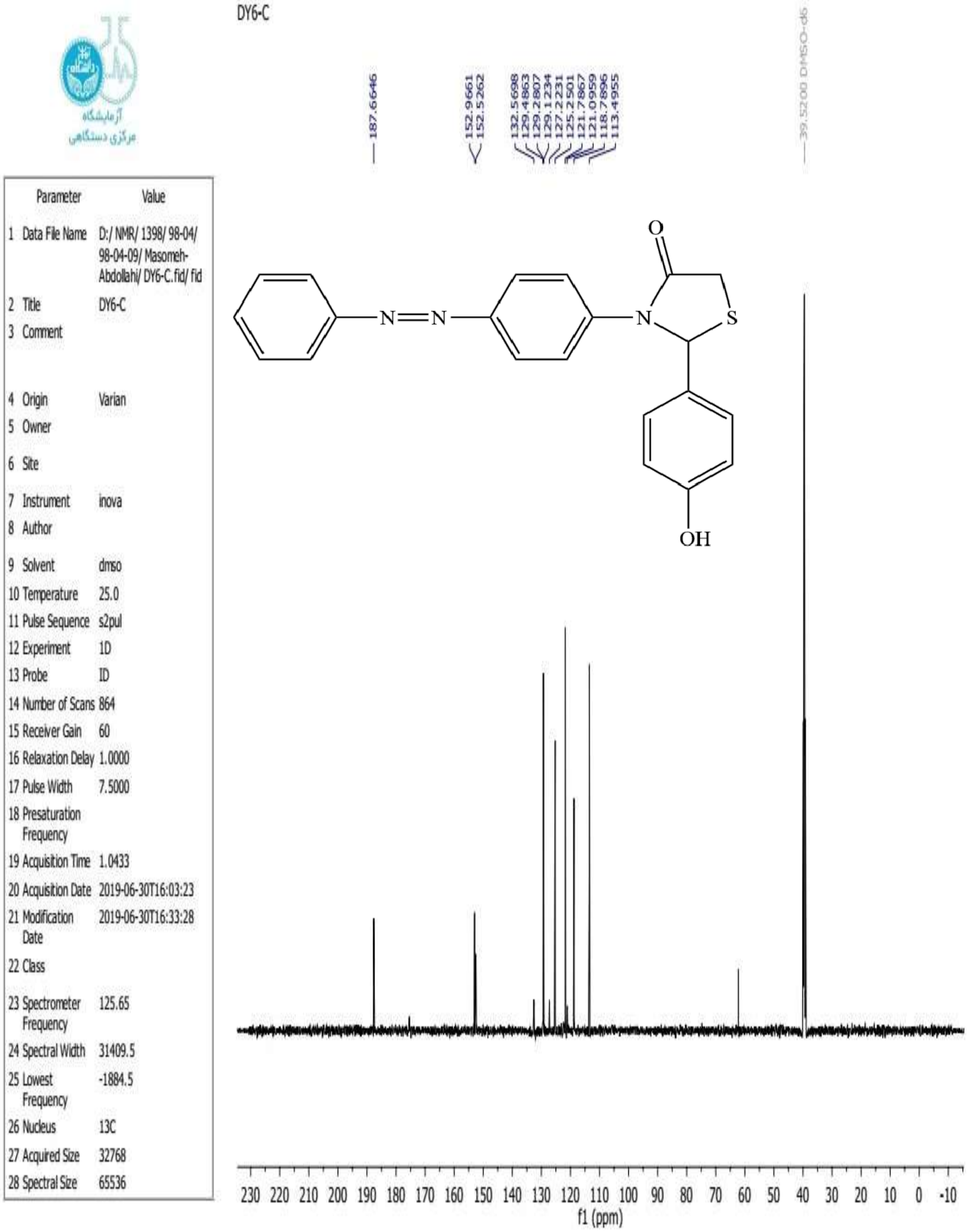

٤-٩- تهیه ی ترکیب ۲-(٤-برمو فنیل)-۳-(٤-(٤-فنیل دیازنیل)فنیل)تیازولیدین-٤-اون (٤c)

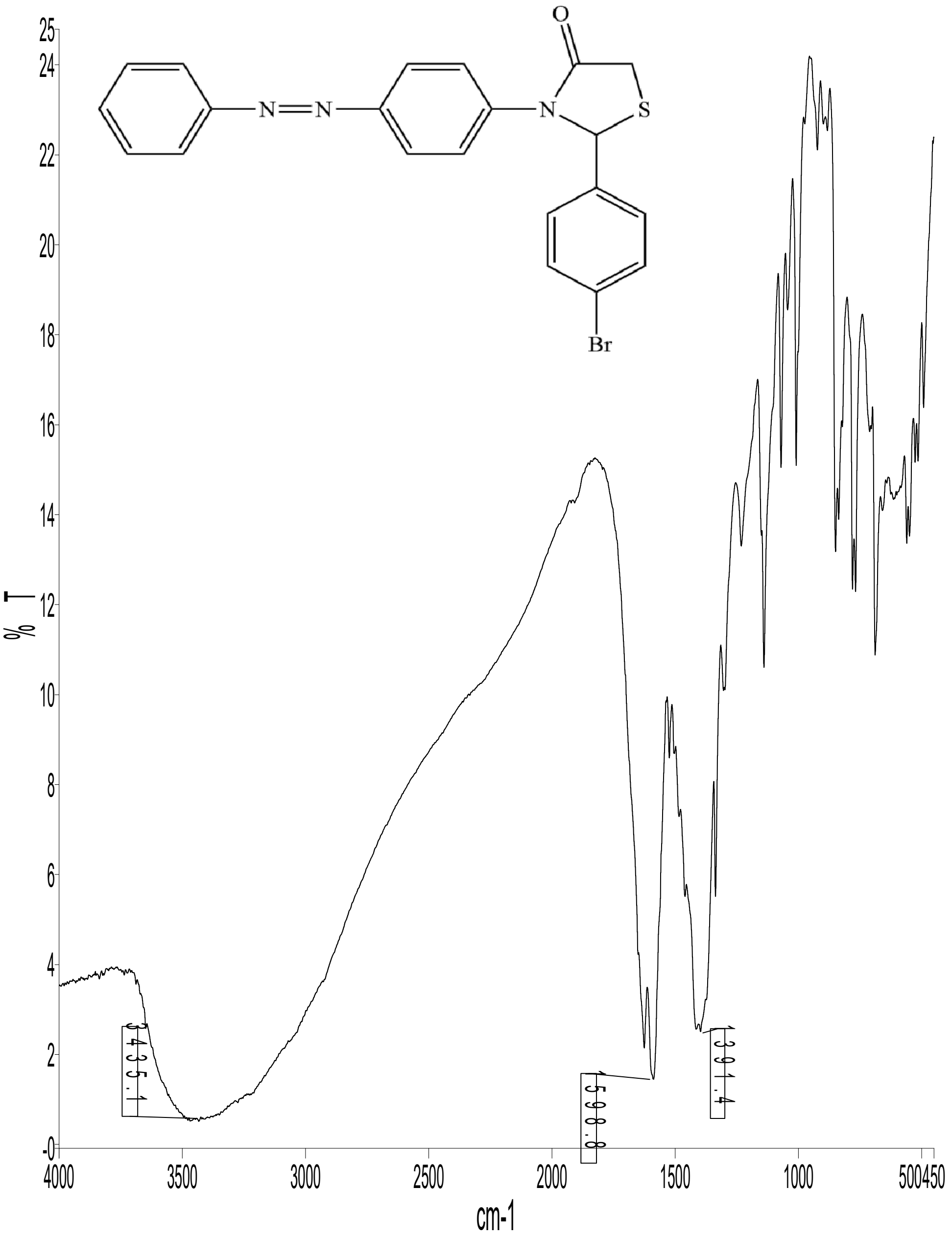

٤-١٠- تهیه ی ترکیب ۲-(٤-برمو فنیل)-۳-(٤-فنیل دیازنیل)فنیل)تیازولیدین-٤-اون (٤c)

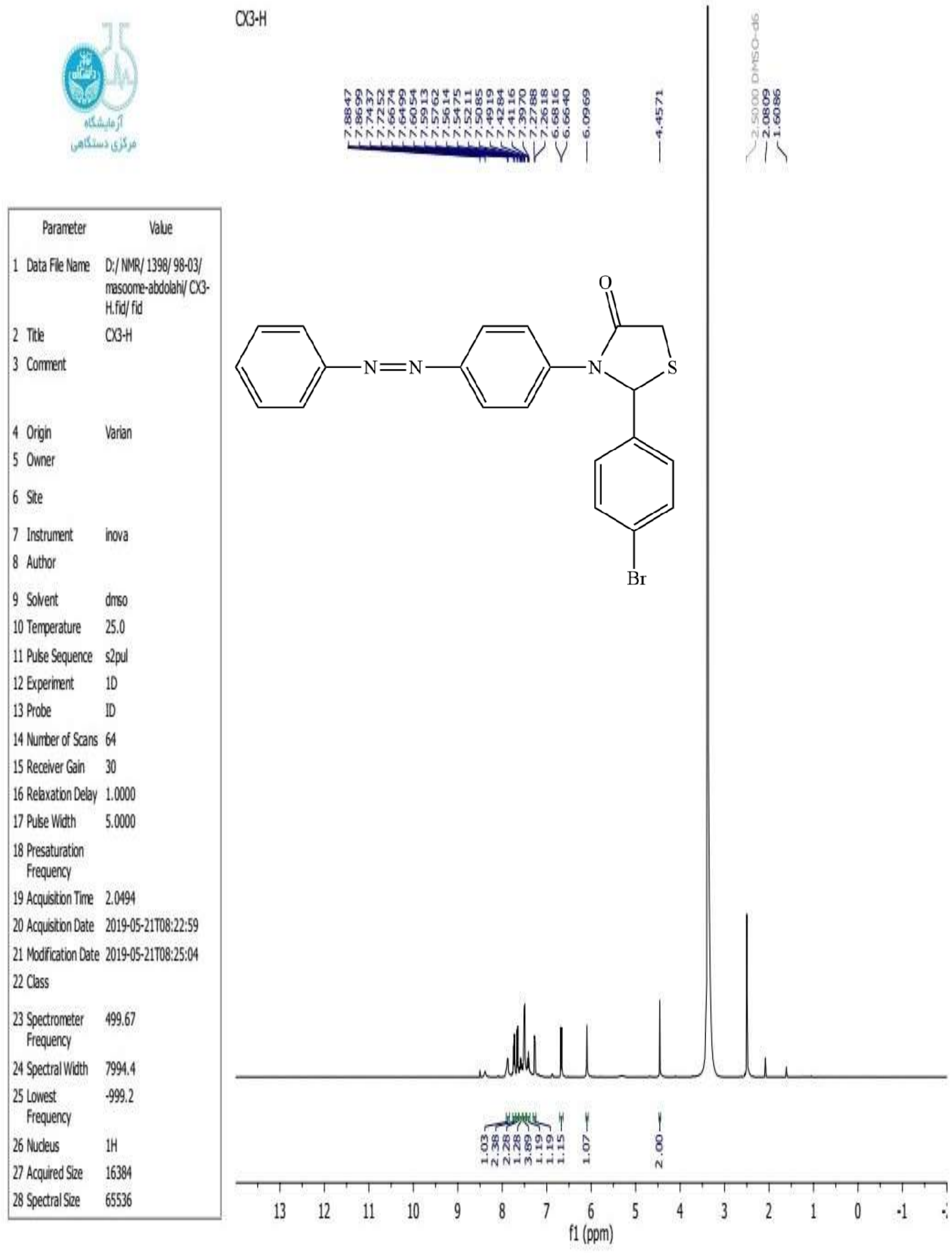

Parameter	Value
1 Data File Name	D:/ NMR/ 1398/ 98-03/ masoome-abdolahi/ CX3-H.fid/ fid
2 Title	CX3-H
3 Comment	
4 Origin	Varian
5 Owner	
6 Site	
7 Instrument	inova
8 Author	
9 Solvent	dmso
10 Temperature	25.0
11 Pulse Sequence	s2pul
12 Experiment	1D
13 Probe	ID
14 Number of Scans	64
15 Receiver Gain	30
16 Relaxation Delay	1.0000
17 Pulse Width	5.0000
18 Presaturation Frequency	
19 Acquisition Time	2.0494
20 Acquisition Date	2019-05-21T08:22:59
21 Modification Date	2019-05-21T08:25:04
22 Class	
23 Spectrometer Frequency	499.67
24 Spectral Width	7994.4
25 Lowest Frequency	-999.2
26 Nucleus	1H
27 Acquired Size	16384
28 Spectral Size	65536

٤-١١- تهیه ی ترکیب ٢-(٤-برمو فنیل)-٣-(٤-فنیل دیازنیل)فنیل)تیازولیدین-٤-اون (٤c)

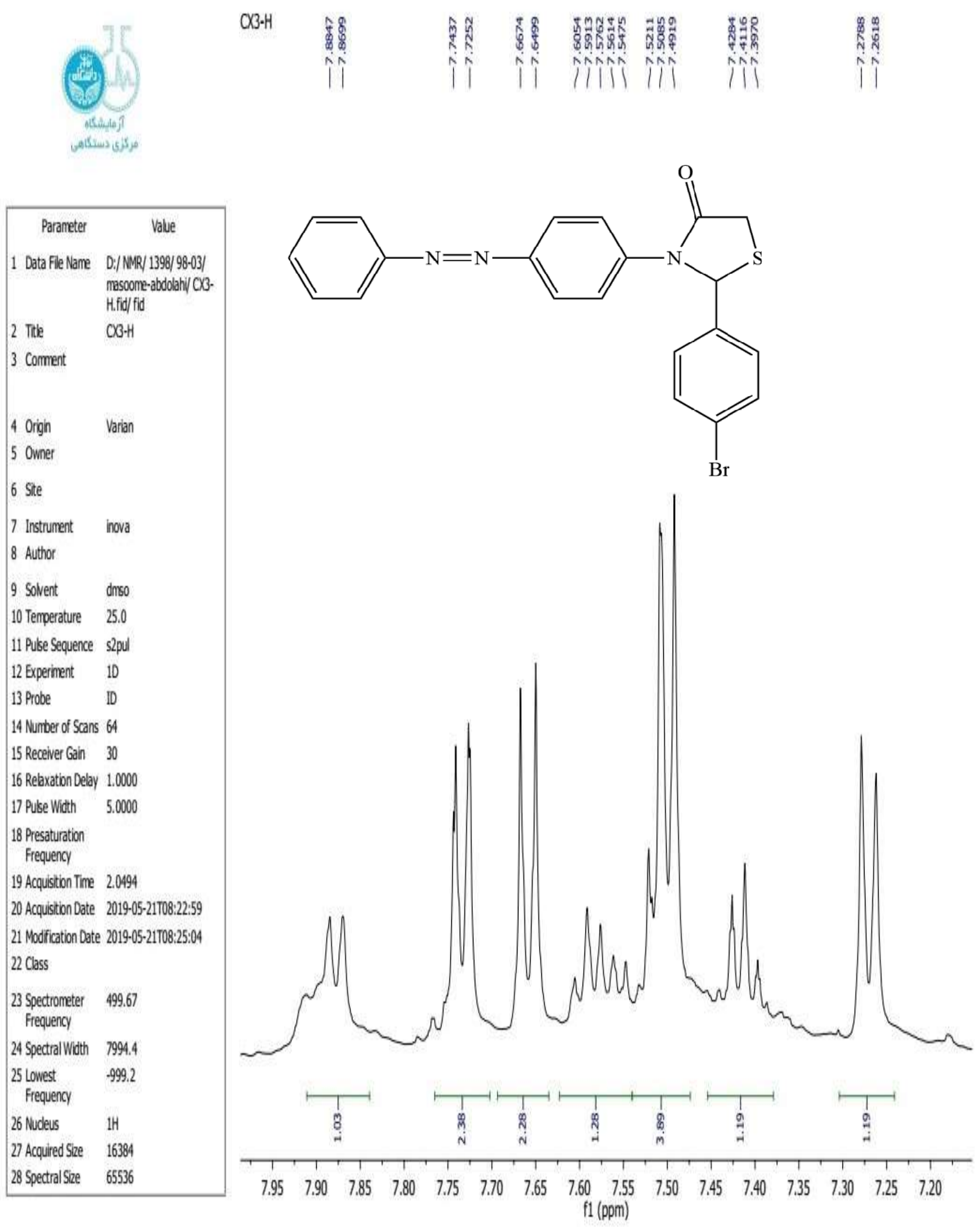

٤-١٢- تهیه ی ترکیب ٢-(٤-برمو فنیل)-٣-(٤-فنیل دیازنیل)فنیل)تیازولیدین-٤-اون (٤c)

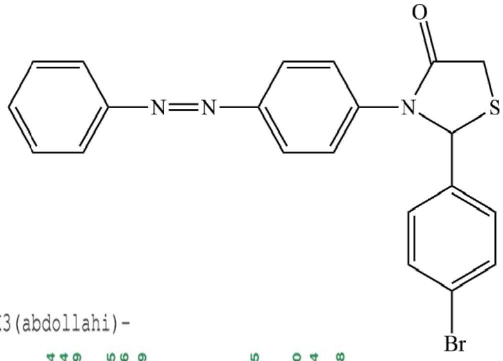

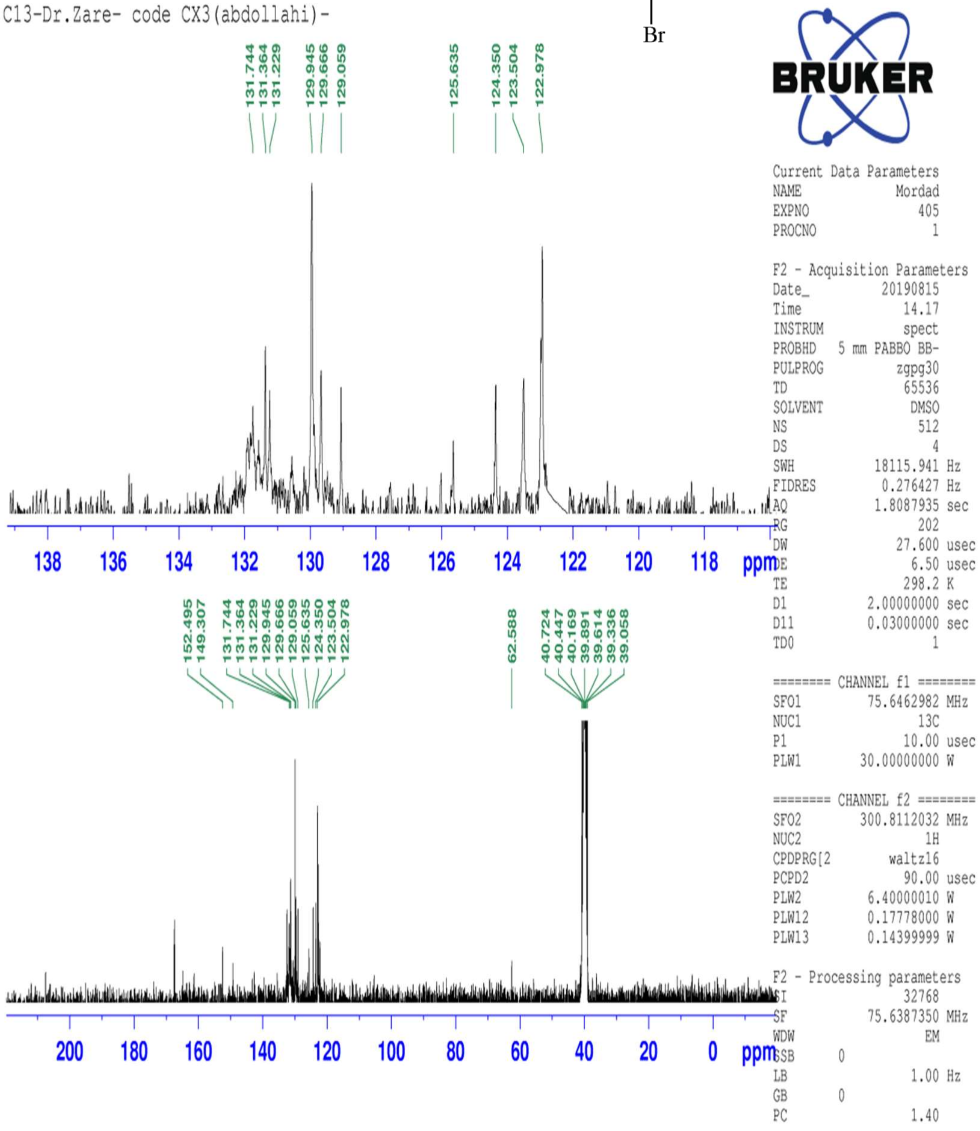

٤-١٣- تهیه ی ترکیب ٢-(٤-کلرو فنیل)-٣-(٤-فنیل دیازنیل)فنیل)تیازولیدین-٤-اون (٤d)

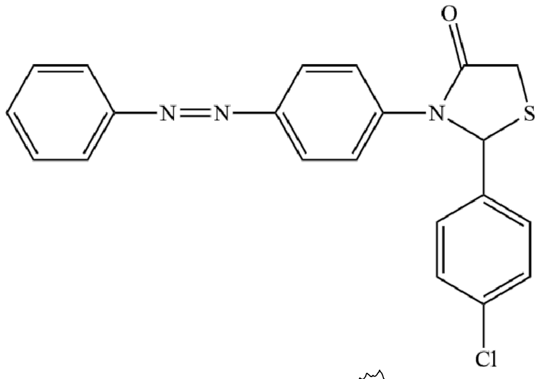

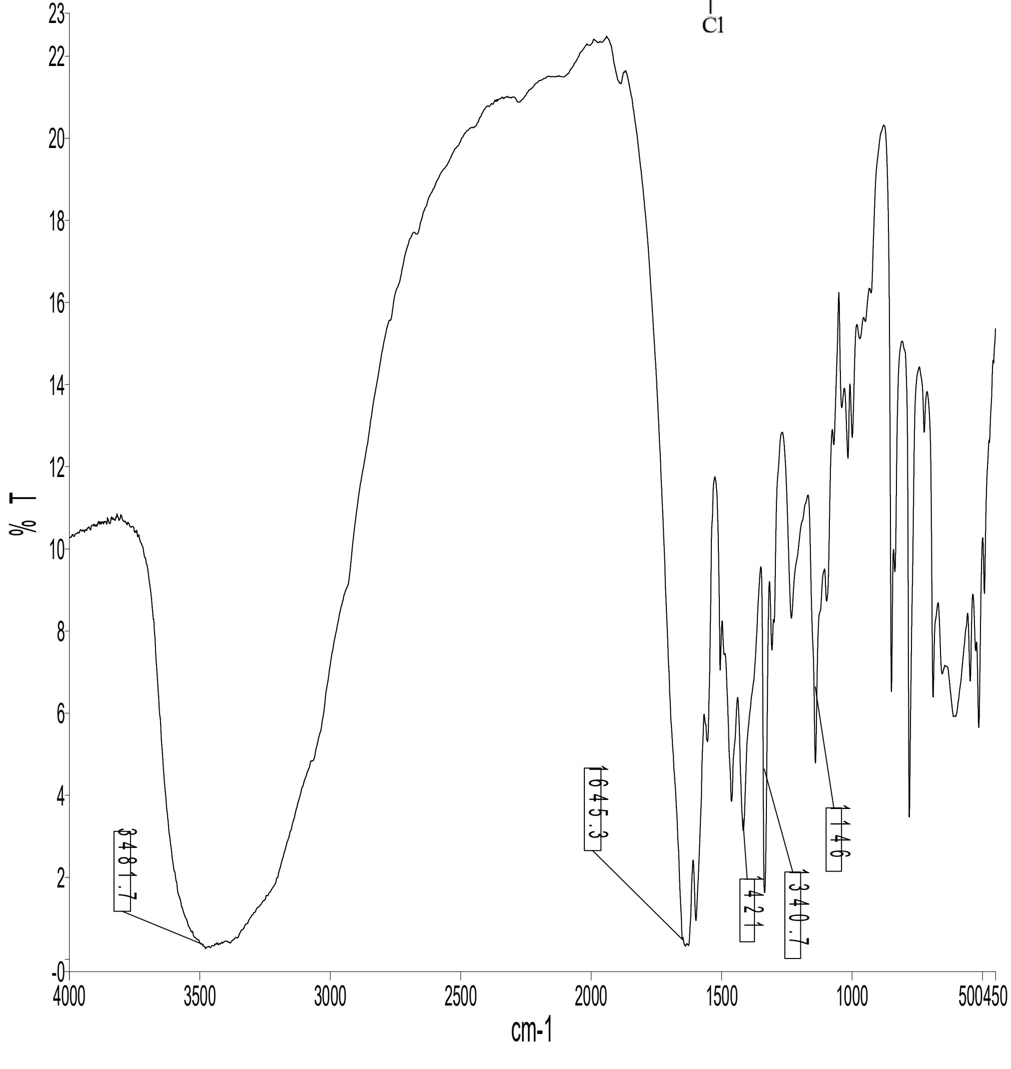

٤-١٤- تهیه ی ترکیب ٢-(٤-کلرو فنیل)-٣-(٤-فنیل دیازنیل)فنیل)تیازولیدین-٤-اون (٤d)

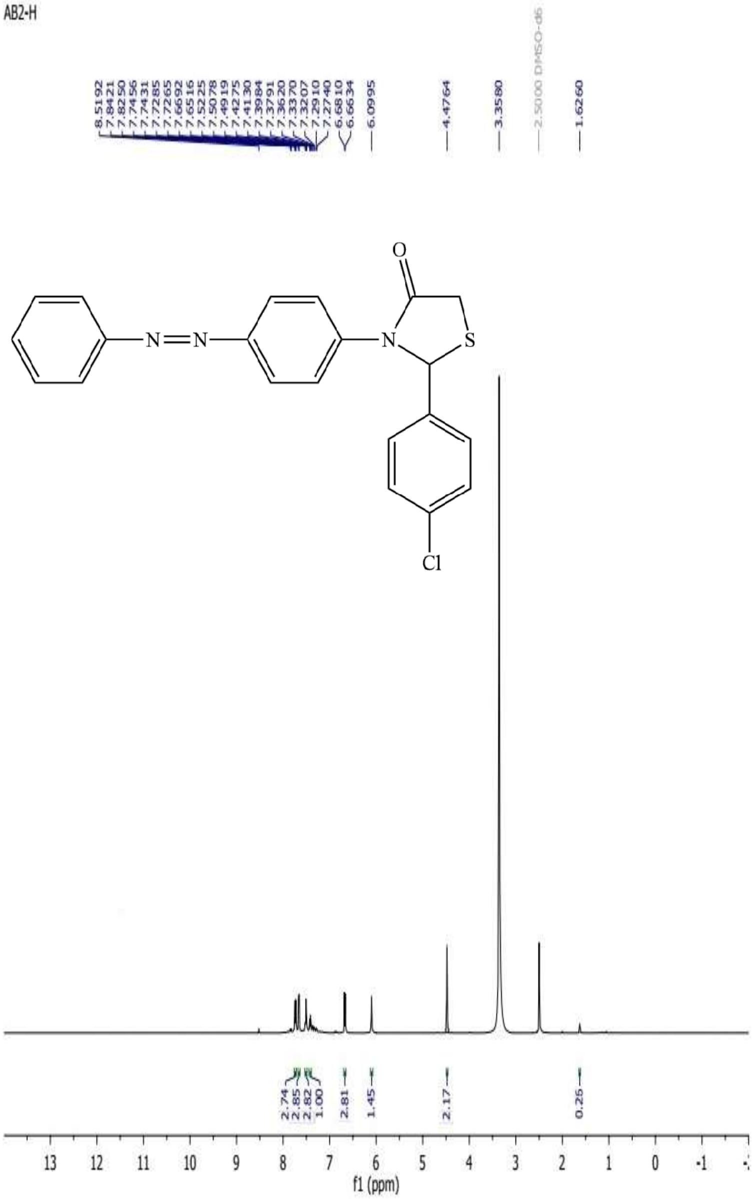

Parameter	Value
1 Data File Name	D:/ NMR/ 1398/ 98-02/ 98-02-31/ 98-02-31/ masoome-abdolahi/ AB2-H.fid/ fid
2 Title	AB2-H
3 Comment	
4 Origin	Varian
5 Owner	
6 Site	
7 Instrument	inova
8 Author	
9 Solvent	dmso
10 Temperature	25.0
11 Pulse Sequence	s2pul
12 Experiment	1D
13 Probe	ID
14 Number of Scans	64
15 Receiver Gain	30
16 Relaxation Delay	1.0000
17 Pulse Width	5.0000
18 Presaturation Frequency	
19 Acquisition Time	2.0494
20 Acquisition Date	2019-05-21T13:31:26
21 Modification Date	2019-05-21T13:31:56
22 Class	
23 Spectrometer Frequency	499.67
24 Spectral Width	7994.4
25 Lowest Frequency	-999.2
26 Nucleus	1H
27 Acquired Size	16384
28 Spectral Size	65536

٤-١٥- تهیه ی ترکیب ٢-(٤-کلرو فنیل)-٣-(٤-فنیل دیازنیل)فنیل)تیازولیدین-٤-اون (٤d)

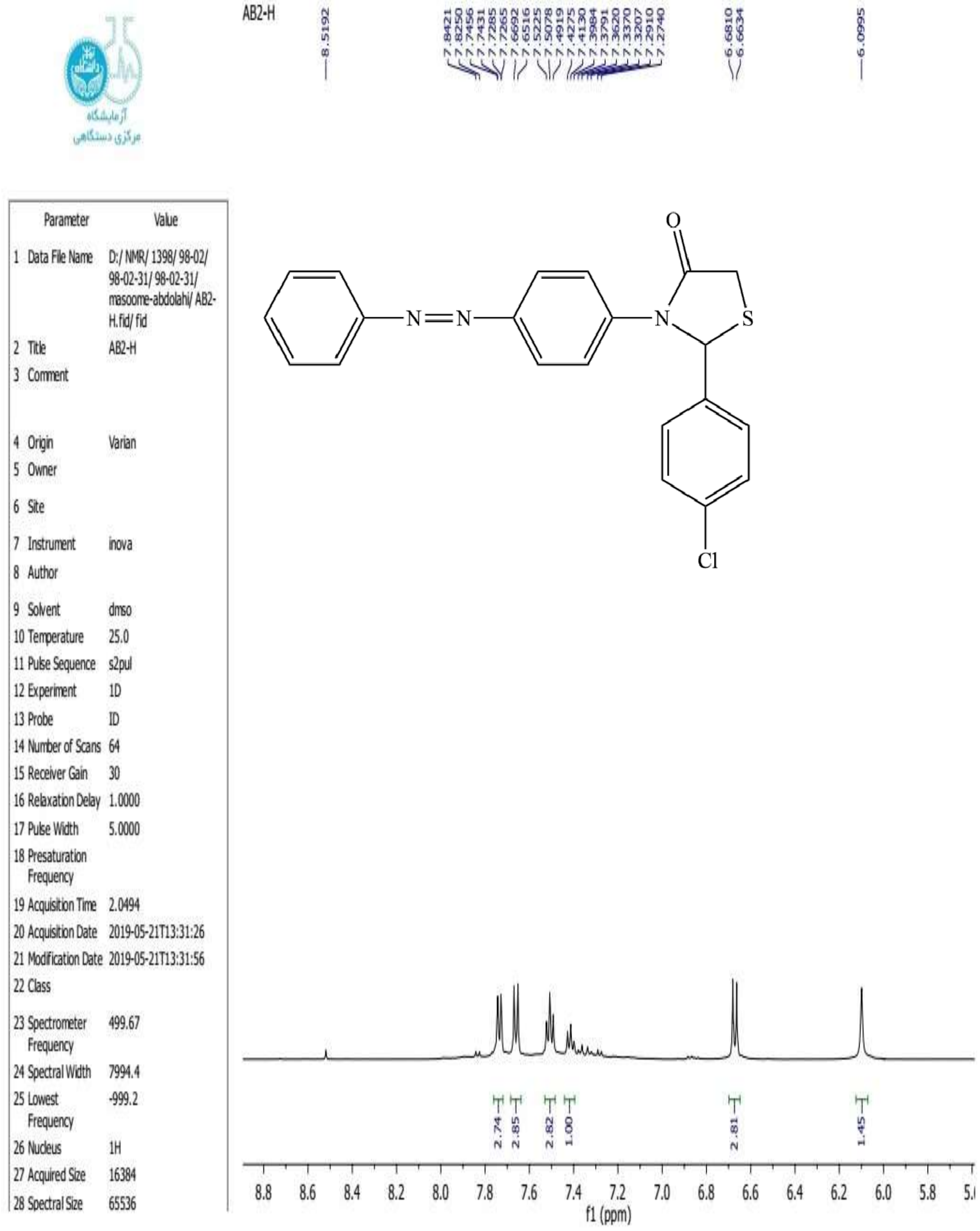

٤-١٦- تهیه ی ترکیب ۲-(٤-کلرو فنیل)-۳-(٤-فنیل دیازنیل)فنیل)تیازولیدین-٤-اون (٤d)

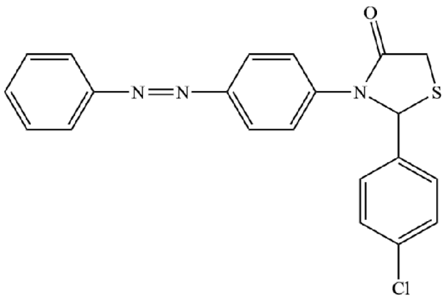

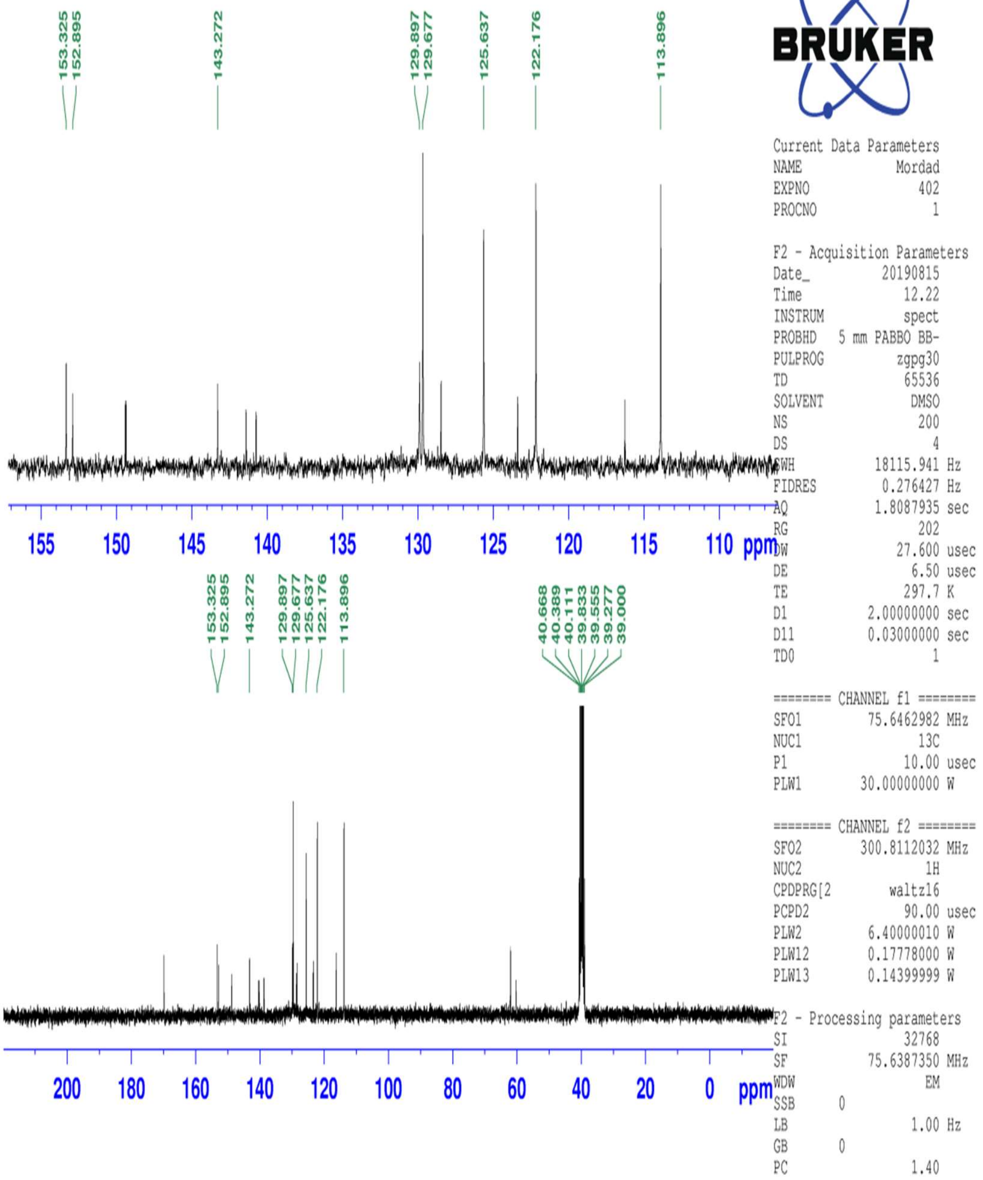

٤-١٧- تهیه ی ترکیب ۲-(٤-دی متیل آمینو)فنیل)-۳-(٤-فنیل دیازنیل)فنیل) تیازولیدین-٤-اون (٤e)

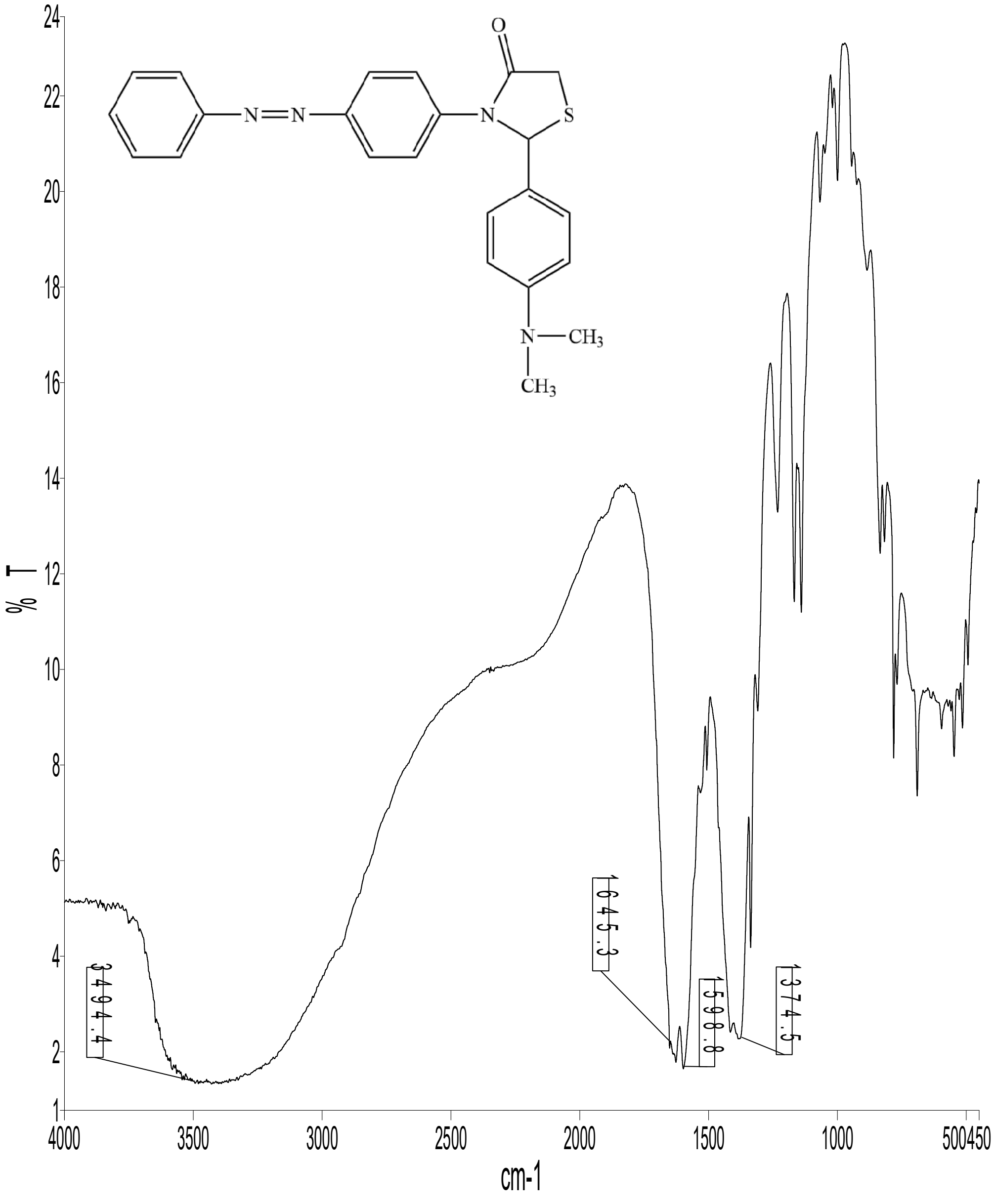

٤-١٨- تهیه ی ترکیب ٢-(٤-دی متیل آمینو)فنیل)-٣-(٤-فنیل دیازنیل)فنیل) تیازولیدین-٤-اون (٤e)

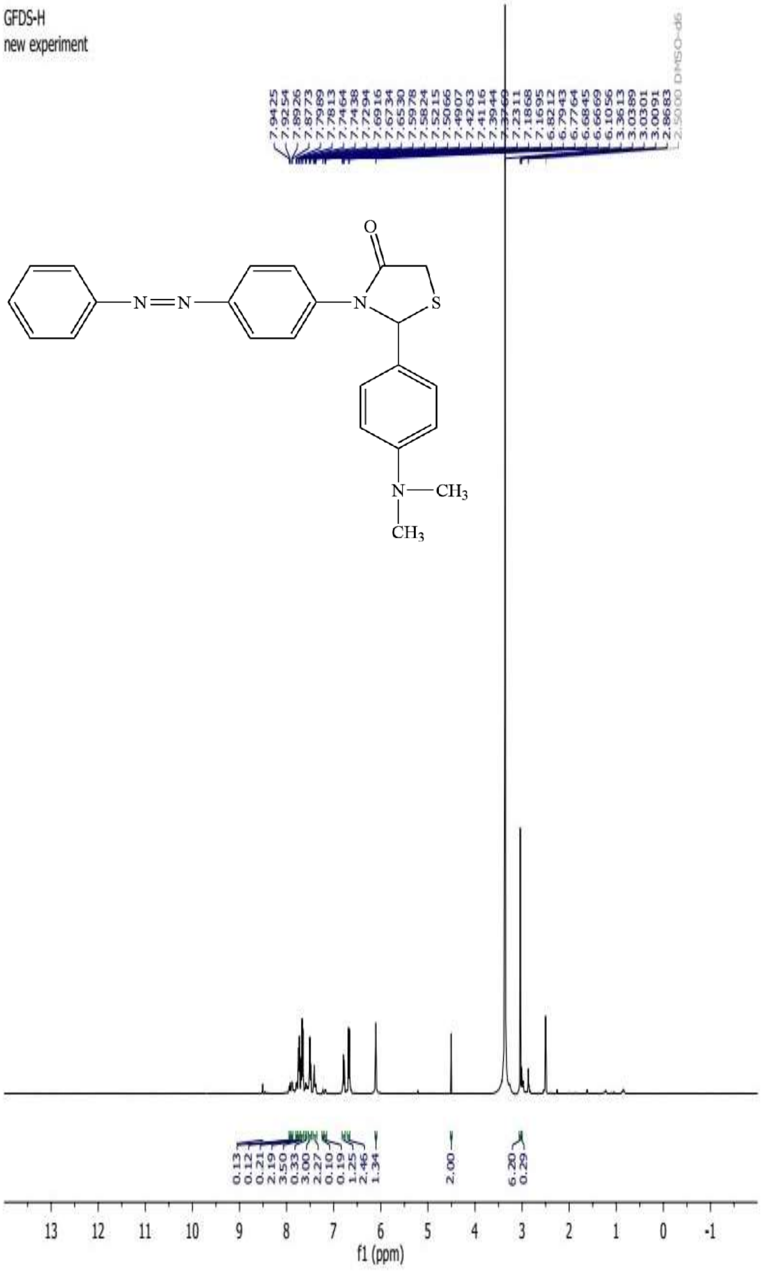

Parameter	Value
1 Data File Name	D:/ NMR/ 1398/ 98-03/ 98-03-25/ Masomeh-'..dollahi/ GFDS-H.fid/ fid
2 Title	GFDS-H
3 Comment	new experiment
4 Origin	Varian
5 Owner	
6 Site	
7 Instrument	inova
8 Author	
9 Solvent	dmso
10 Temperature	25.0
11 Pulse Sequence	s2pul
12 Experiment	1D
13 Probe	ID
14 Number of Scans	8
15 Receiver Gain	30
16 Relaxation Delay	1.0000
17 Pulse Width	5.0000
18 Presaturation Frequency	
19 Acquisition Time	2.0494
20 Acquisition Date	2019-06-15T14:26:14
21 Modification Date	2019-06-15T14:27:38
22 Class	
23 Spectrometer Frequency	499.66
24 Spectral Width	7994.4
25 Lowest Frequency	-999.2
26 Nucleus	1H
27 Acquired Size	16384
28 Spectral Size	65536

٤-١٩- تهیه ی ترکیب ۲-(٤-دی متیل آمینو)فنیل)-۳-(٤-فنیل دیازنیل)فنیل) تیازولیدین-٤-اون (٤e)

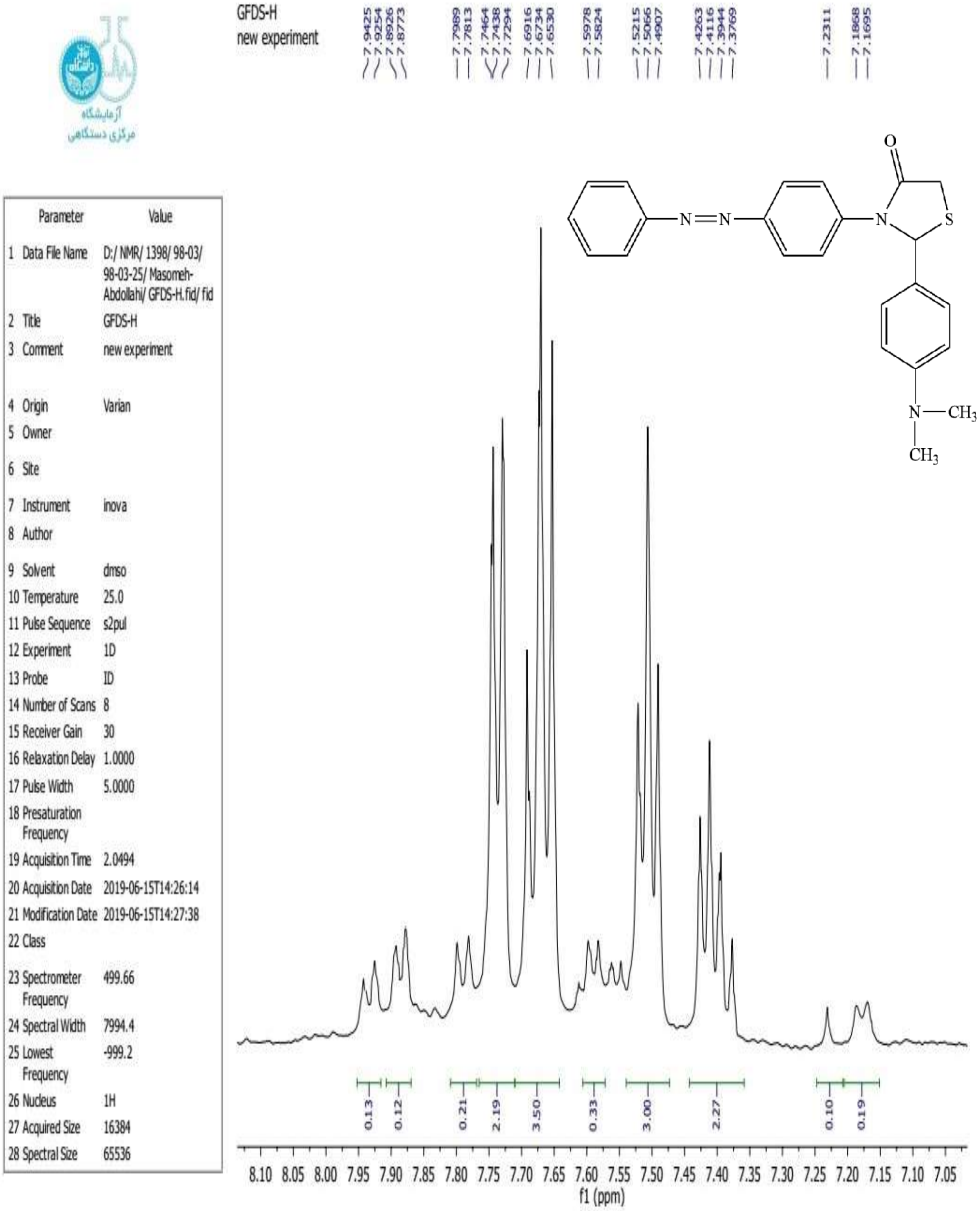

٤-٢٠- تهیه ی ترکیب ۲-(٤-دی متیل آمینو)فنیل)-۳-(٤-فنیل دیازنیل)فنیل) تیازولیدین-٤-اون (٤e)

File : C:\MSDCHEM\3\DATA\Snapshot\30001822.D
Operator : taghizadeh
Acquired : 9 Jul 2019 15:09 using AcqMethod PAH
Instrument : Instrumen
Sample Name: GFDS
Misc Info :
Vial Number: 1

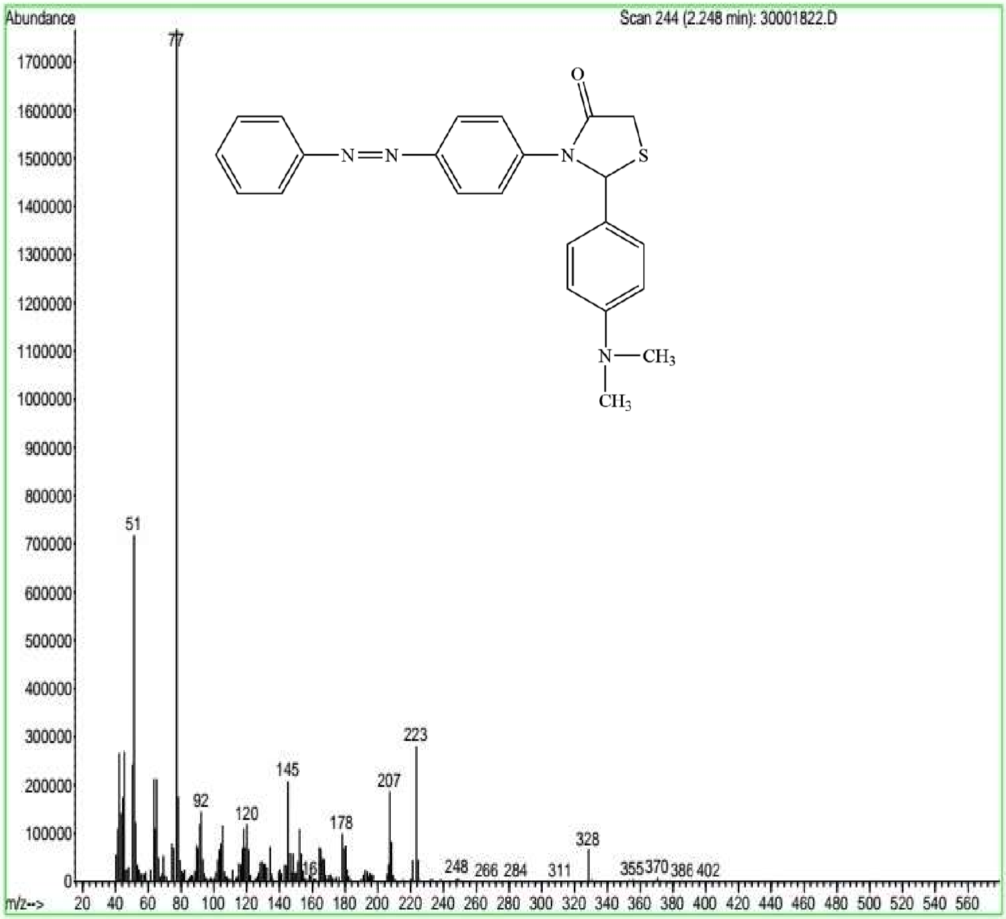

PDF Creator - PDF4Free v3.0 http://www.pdf4free.com

٤-٢١- تهیه ی ترکیب ۲-(۳-کلرو فنیل)-۳-(٤-فنیل دیازنیل)فنیل)تیازولیدین-٤-اون (4f)

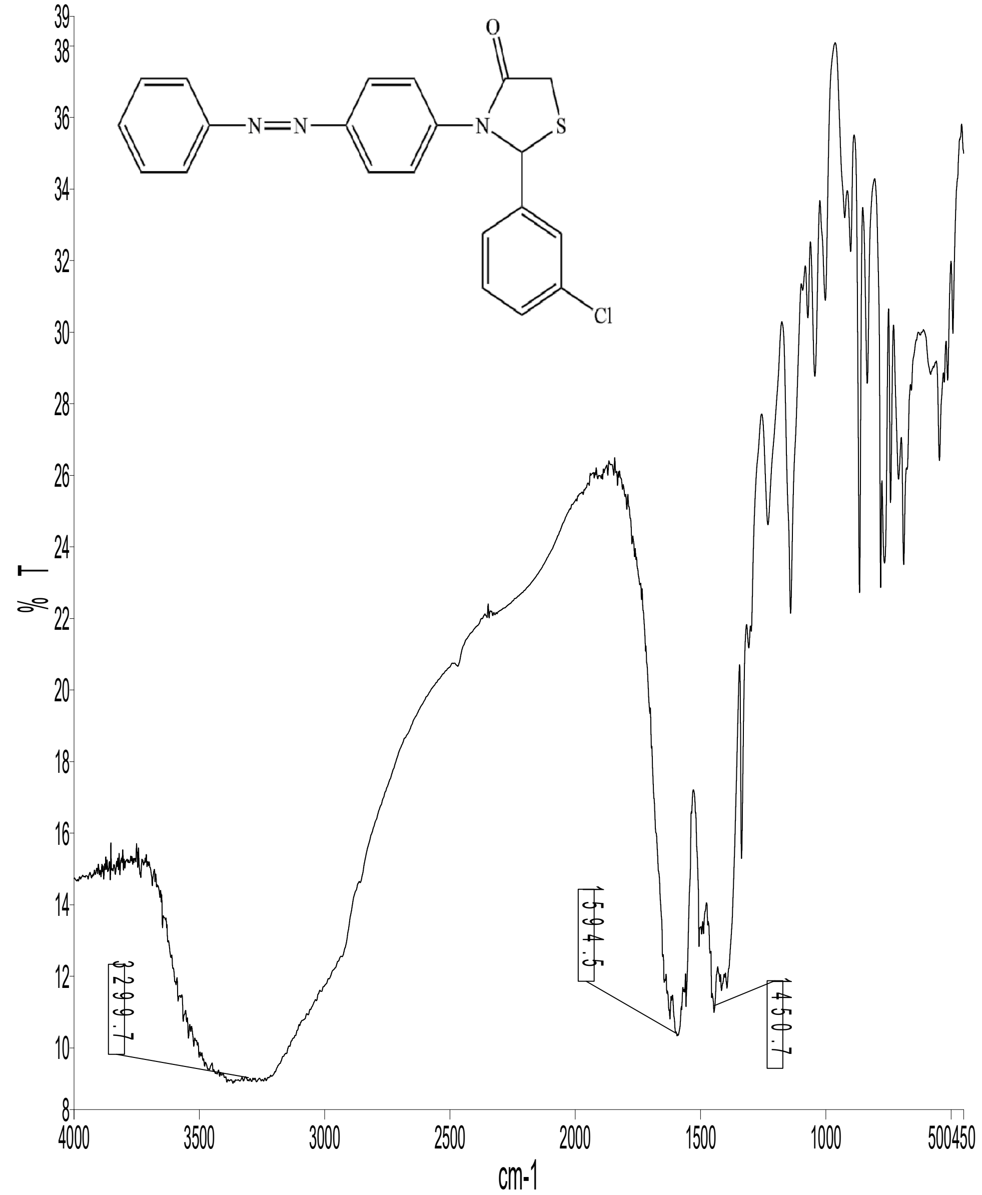

٤-٢٢- تهیه ی ترکیب ٢-(٣-کلرو فنیل)-٣-(٤-فنیل دیازنیل)فنیل)تیازولیدین-٤-اون (٤f)

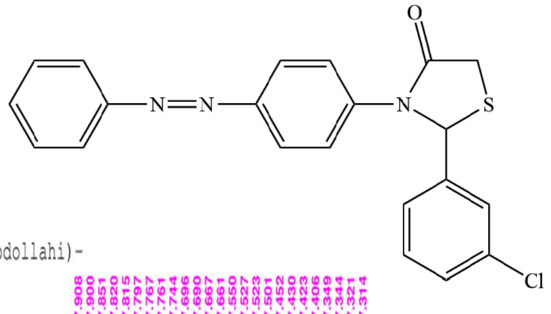

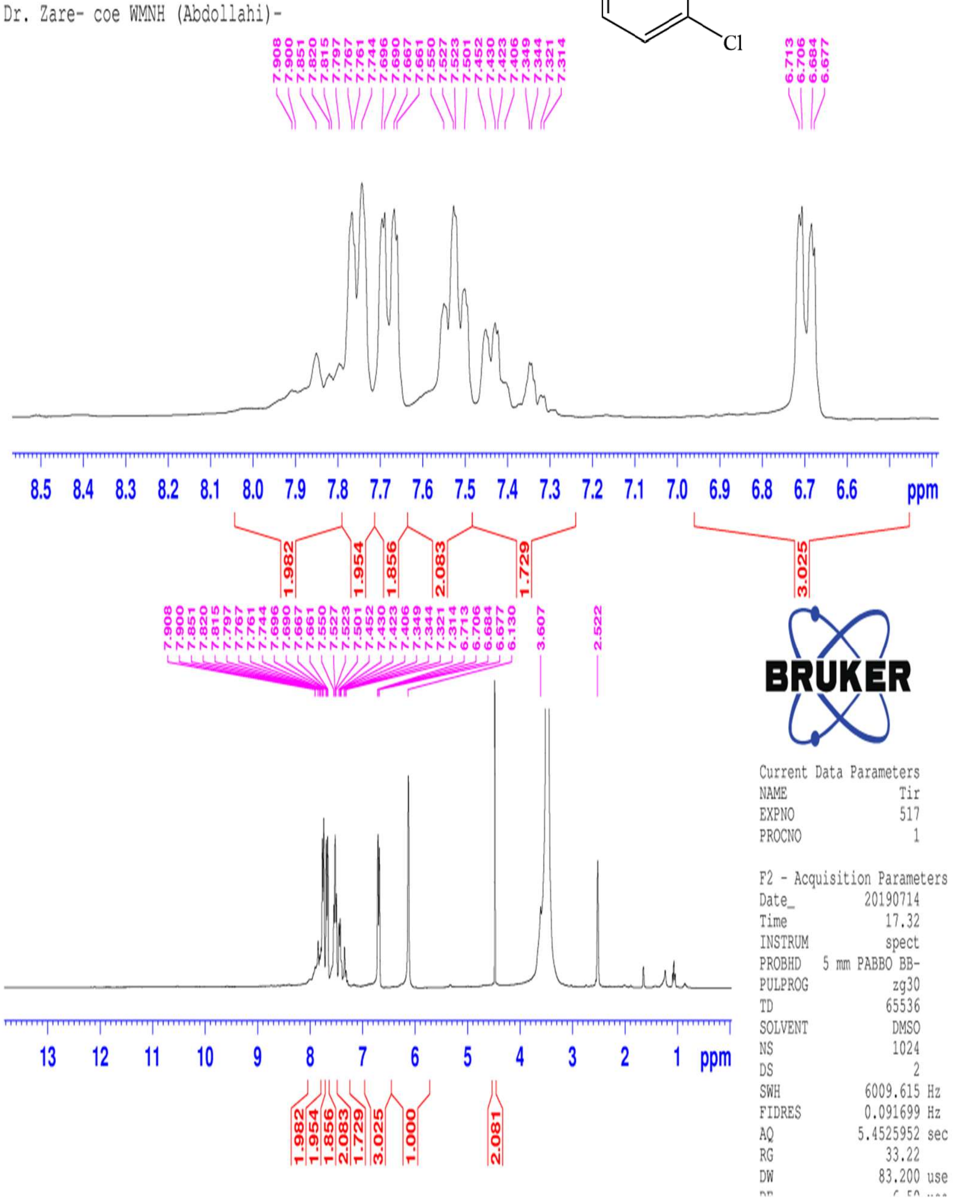

٤-٢٣- تهیه ی ترکیب ۲-(۳-کلرو فنیل)-۳-(٤-فنیل دیازنیل)فنیل)تیازولیدین-٤-اون (٤f)

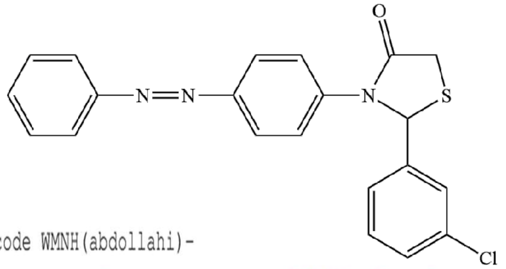

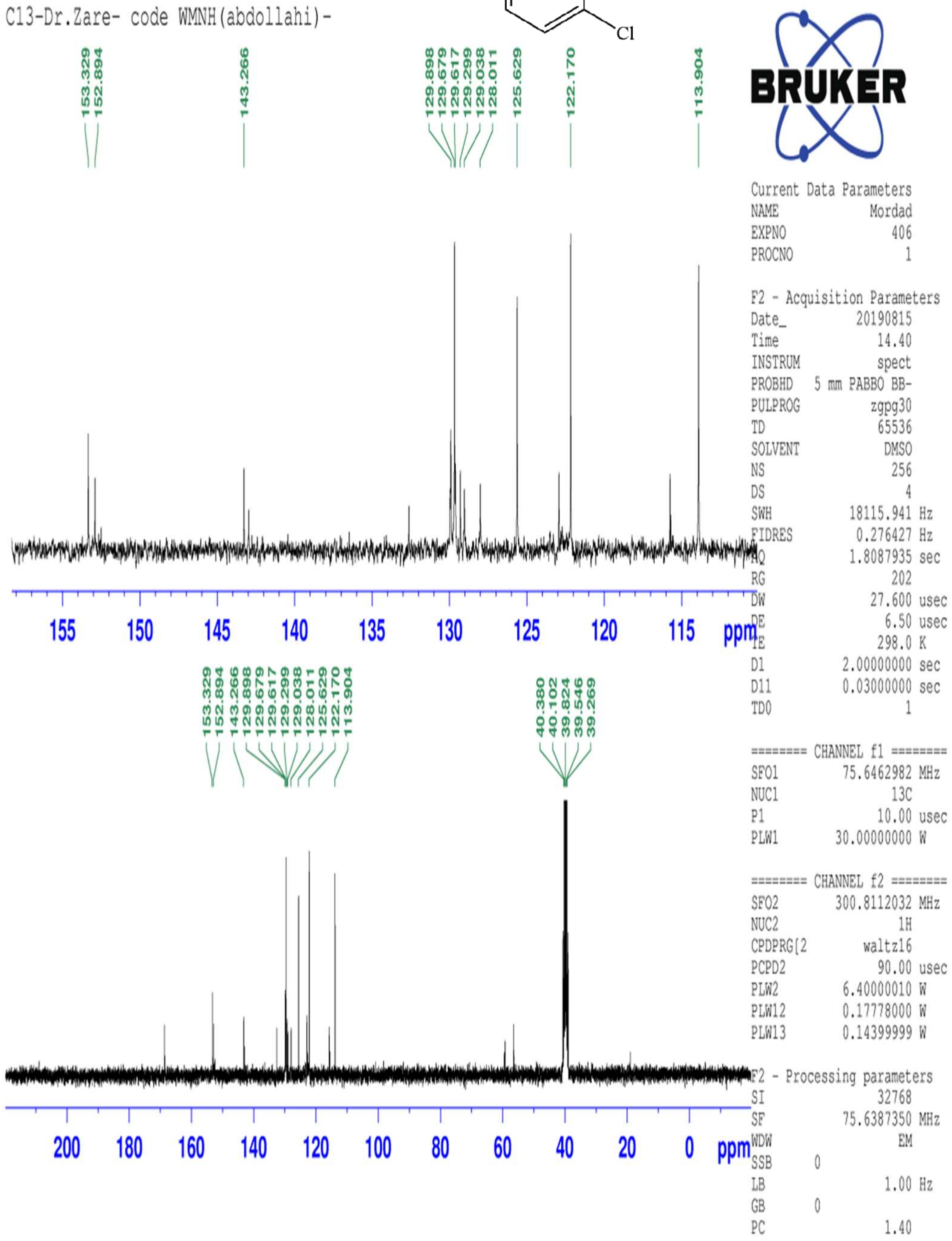

٤-٢٤- تهیه ی ترکیب ٢-(٤-متوکسی فنیل)-٣-(٤-فنیل دیازنیل)فنیل)تیازولیدین-٤-اون (٤g)

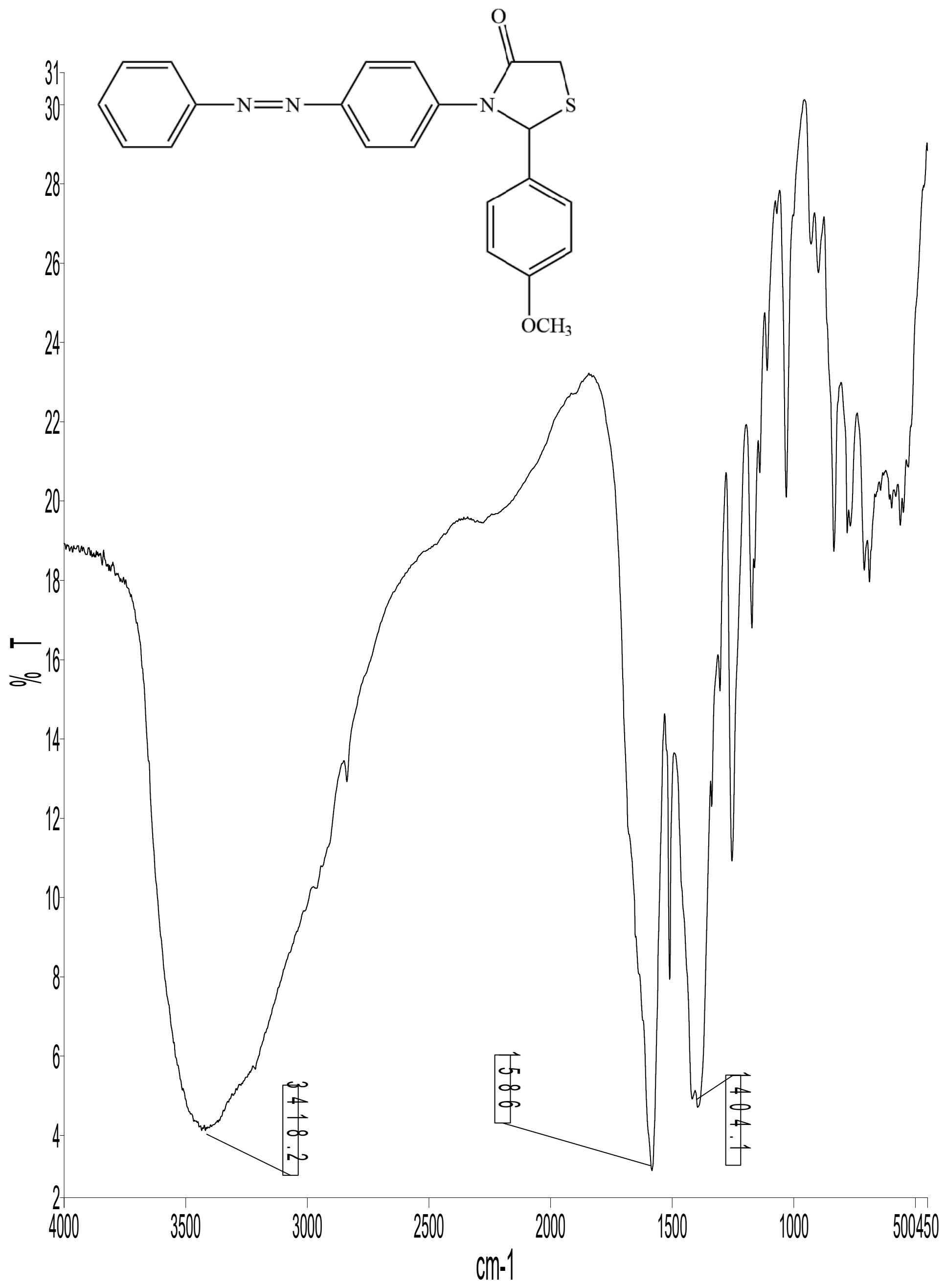

٤-٢٥- تهیه ی ترکیب ۲-(٤-متوکسی فنیل)-۳-(٤-فنیل دیازنیل)فنیل)تیازولیدین-٤-اون (٤g)

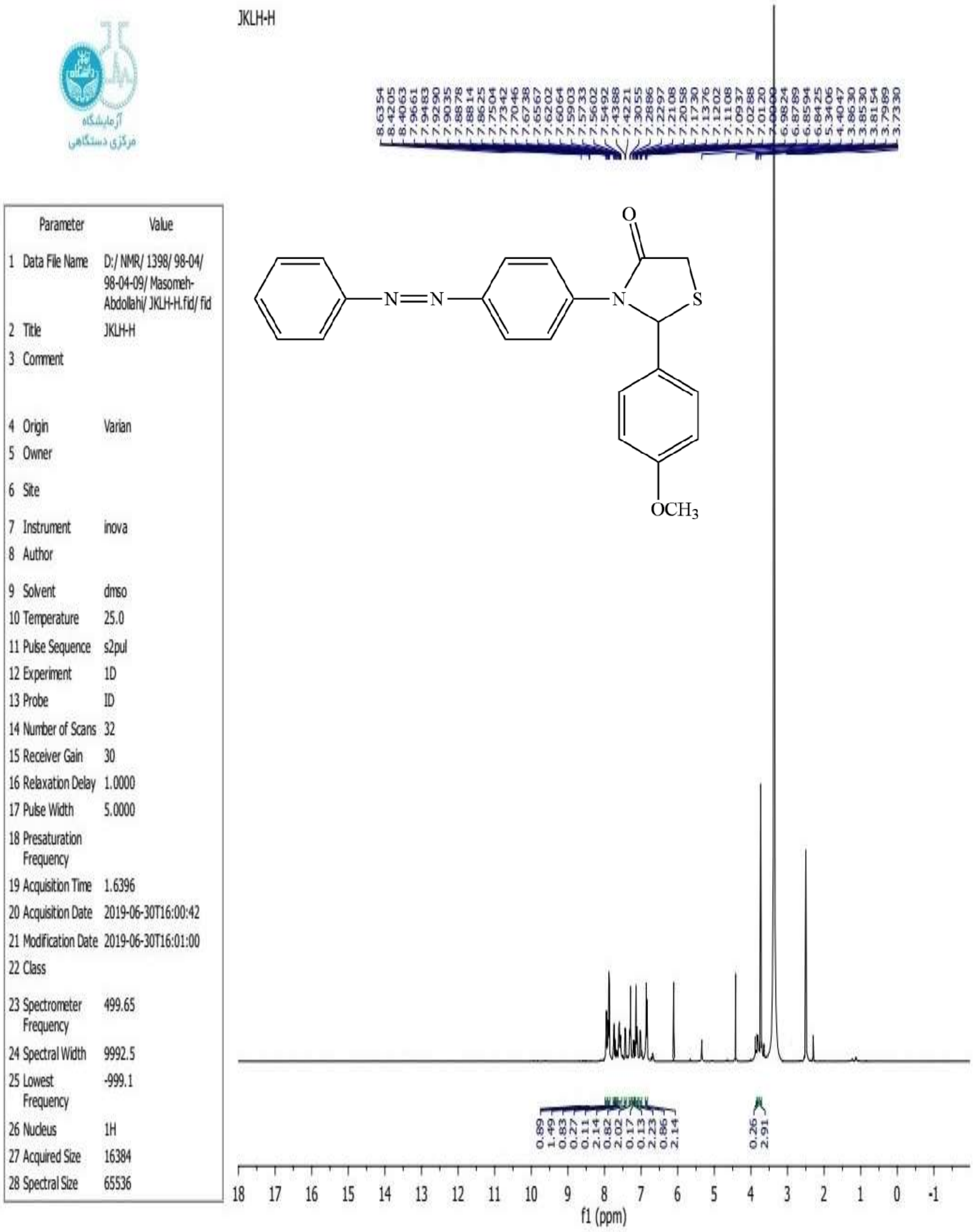

٤-٢٦- تهیه ی ترکیب ٢-(٤-متوکسی فنیل)-٣-(٤-فنیل دیازنیل)فنیل)تیازولیدین-٤-اون (٤g)

Parameter	Value
1 Data File Name	D:/ NMR/ 1398/ 98-04/ 98-04-09/ Masomeh-Abdollahi/ JKLH-H.fid/ fid
2 Title	JKLH-H
3 Comment	
4 Origin	Varian
5 Owner	
6 Site	
7 Instrument	inova
8 Author	
9 Solvent	dmso
10 Temperature	25.0
11 Pulse Sequence	s2pul
12 Experiment	1D
13 Probe	ID
14 Number of Scans	32
15 Receiver Gain	30
16 Relaxation Delay	1.0000
17 Pulse Width	5.0000
18 Presaturation Frequency	
19 Acquisition Time	1.6396
20 Acquisition Date	2019-06-30T16:00:42
21 Modification Date	2019-06-30T16:01:00
22 Class	
23 Spectrometer Frequency	499.65
24 Spectral Width	9992.5
25 Lowest Frequency	-999.1
26 Nucleus	1H
27 Acquired Size	16384
28 Spectral Size	65536

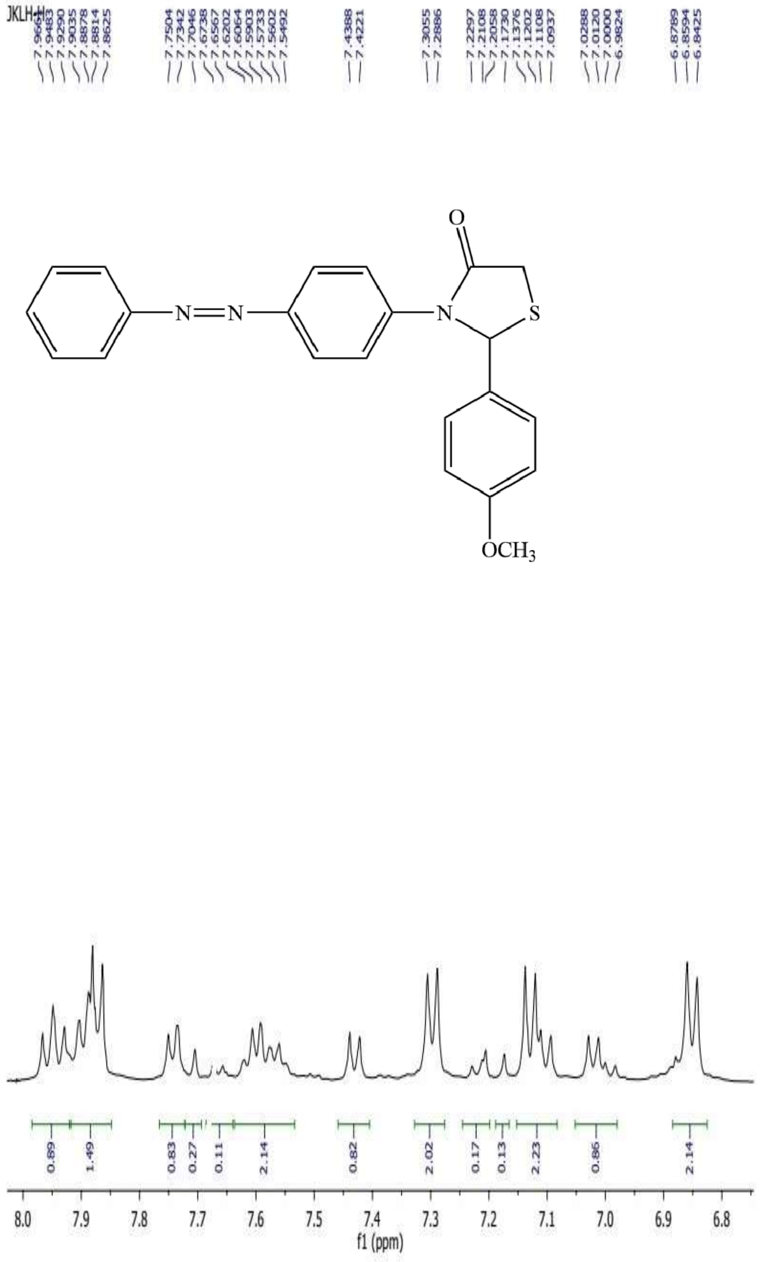

٤-۲۷- تهیه ی ترکیب ۲-(٤-متوکسی فنیل)-۳-(٤-فنیل دیازنیل)فنیل)تیازولیدین-٤-اون (٤g)

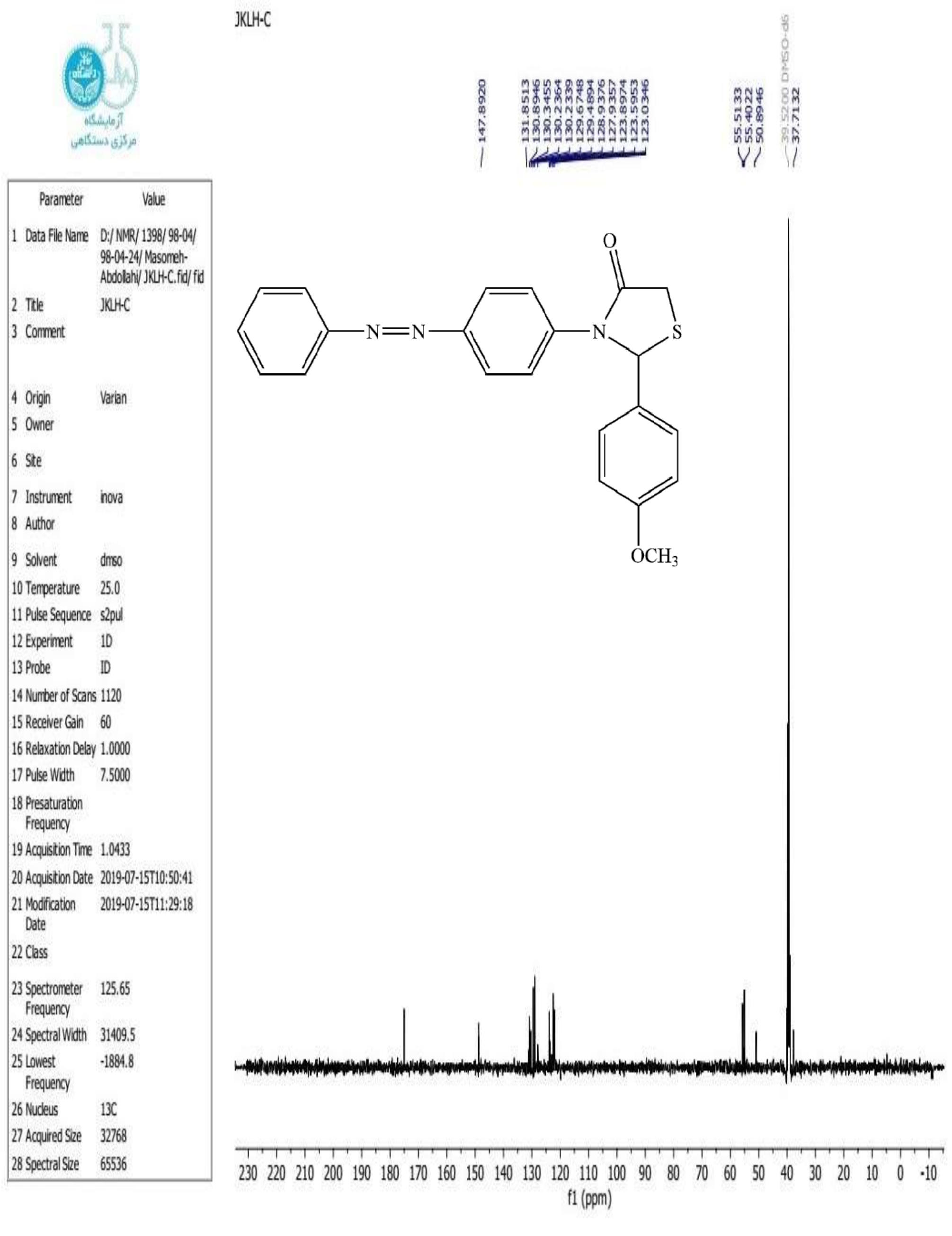

٤-٢٨- تهیه ی ترکیب ٢-(٢-کلرو فنیل)-٣-(٤-فنیل دیازنیل)فنیل)تیازولیدین-٤-اون (٤h)

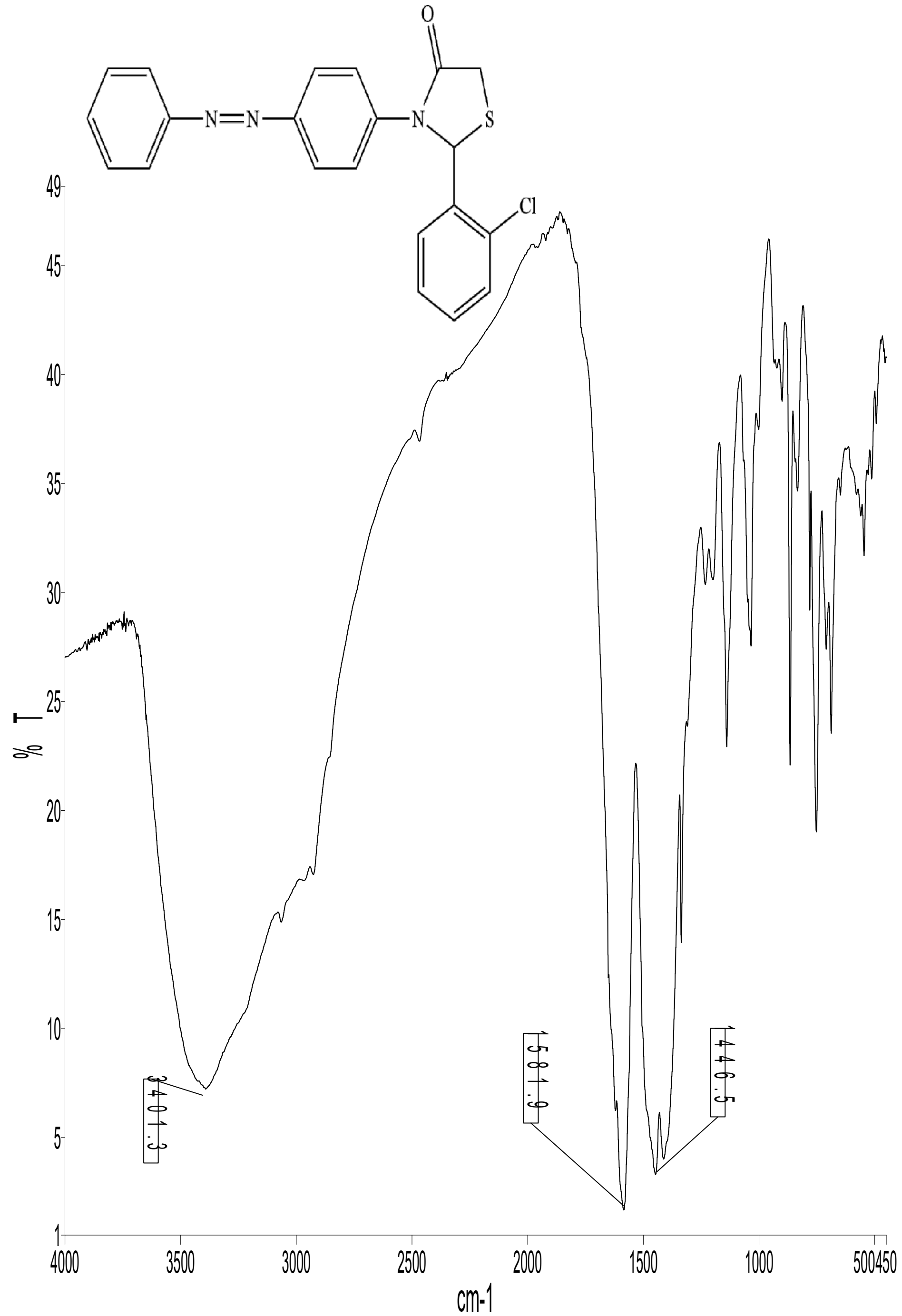

٤-۲۹- تهیه ی ترکیب ۲-(۲-کلرو فنیل)-۳-(٤-فنیل دیازنیل)فنیل)تیازولیدین-٤-اون (٤h)

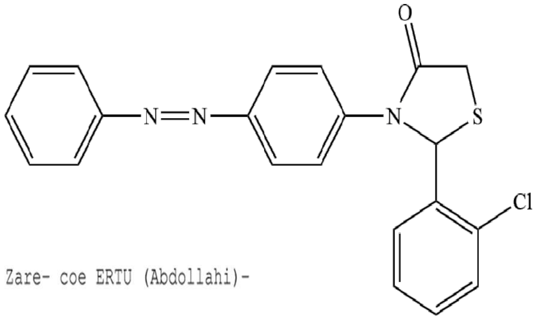

Dr. Zare- coe ERTU (Abdollahi)-

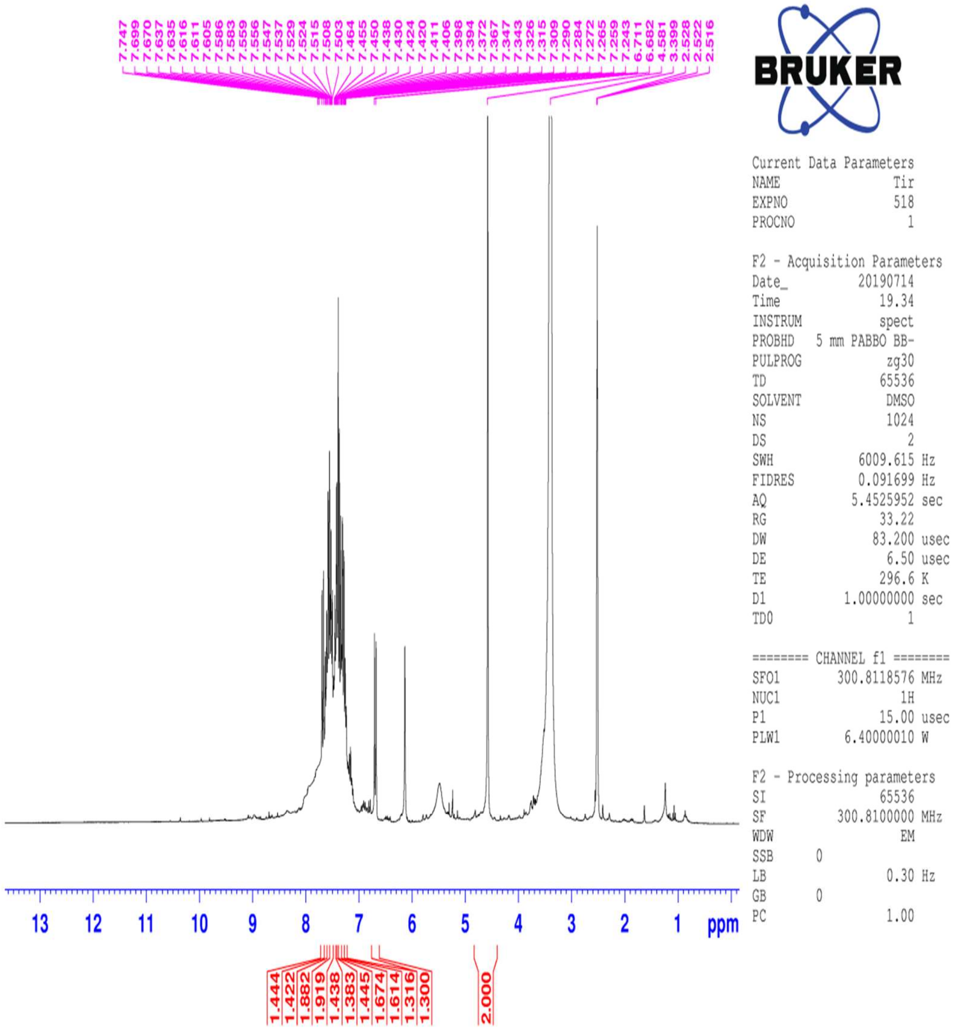

٤-٣٠- تهیه ی ترکیب ٢-(٢-کلرو فنیل)-٣-(٤-فنیل دیازنیل)فنیل)تیازولیدین-٤-اون (٤h)

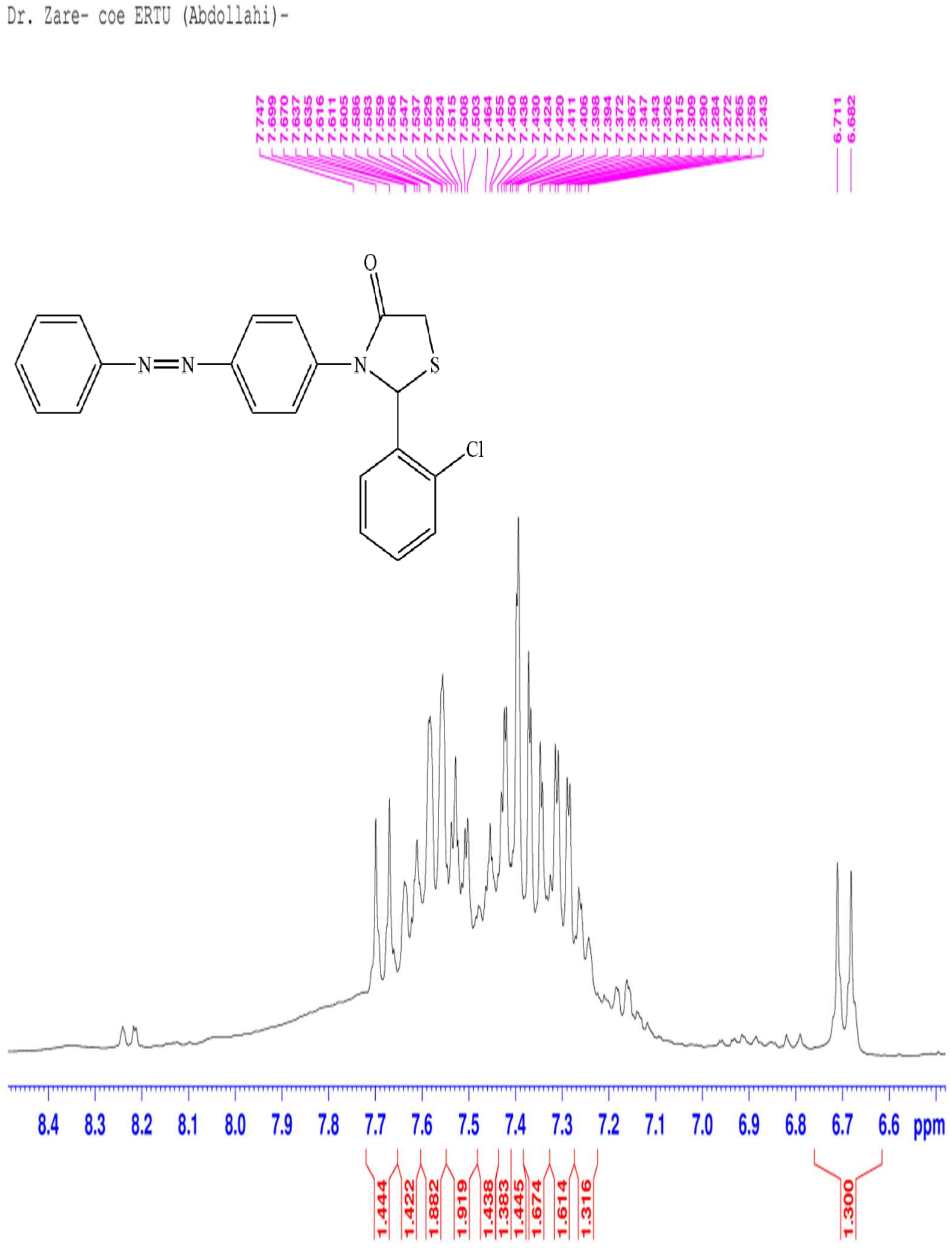

٤-٣١- تهیه ی ترکیب ٢-(٢-کلرو فنیل)-٣-(٤-فنیل دیازنیل)فنیل)تیازولیدین-٤-اون (٤h)

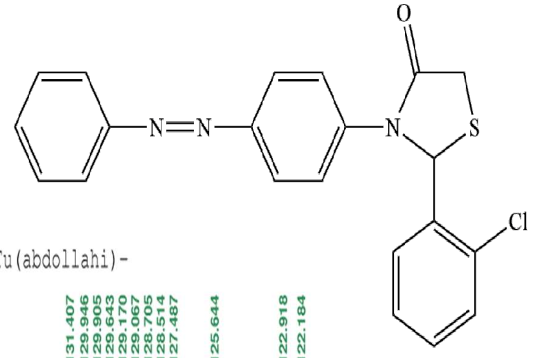

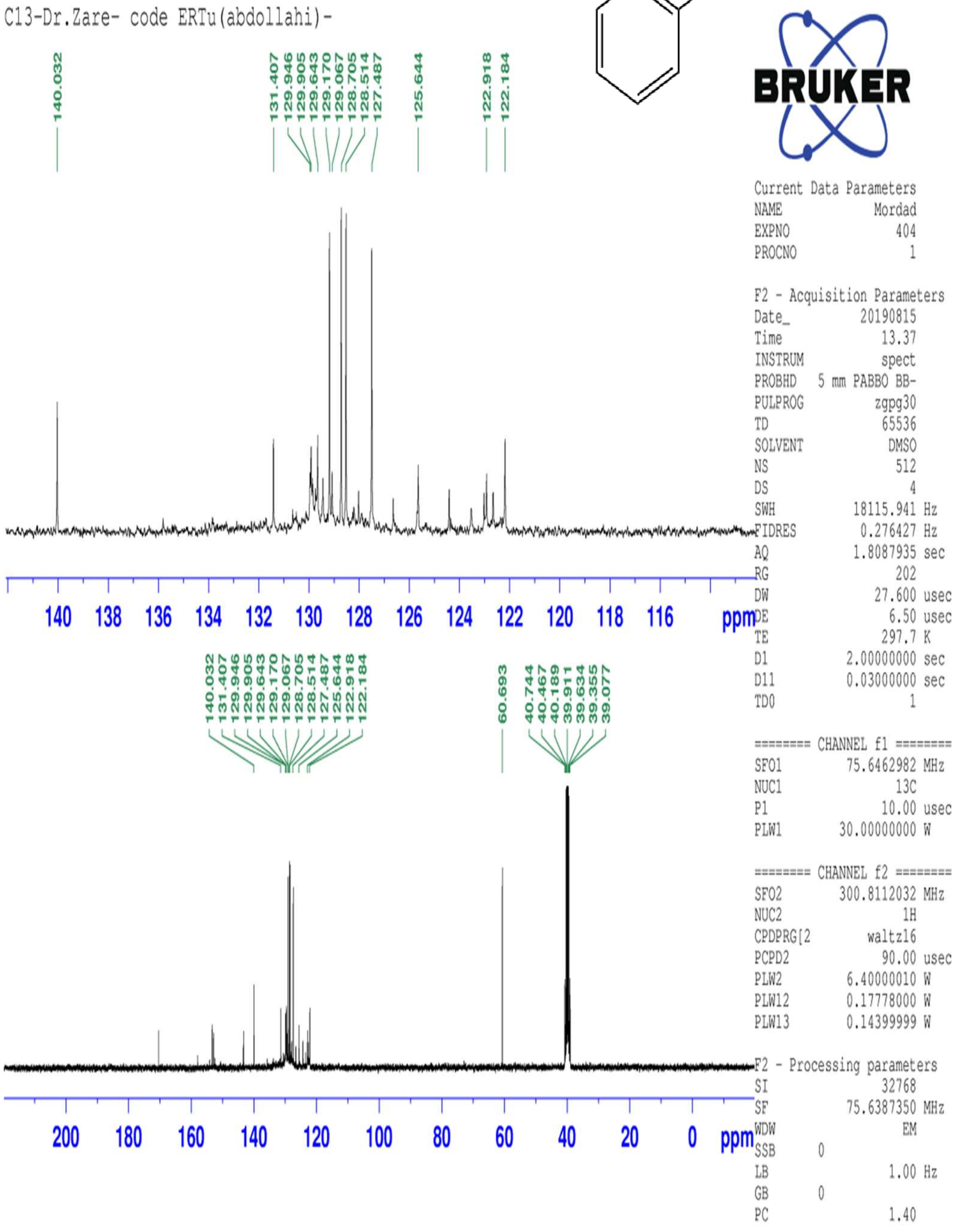

٤-٣٢- تهیه ی ترکیب ٢-(٣-نیترو فنیل)-٣-(٤-فنیل دیازنیل)فنیل)تیازولیدین-٤-اون (٤i)

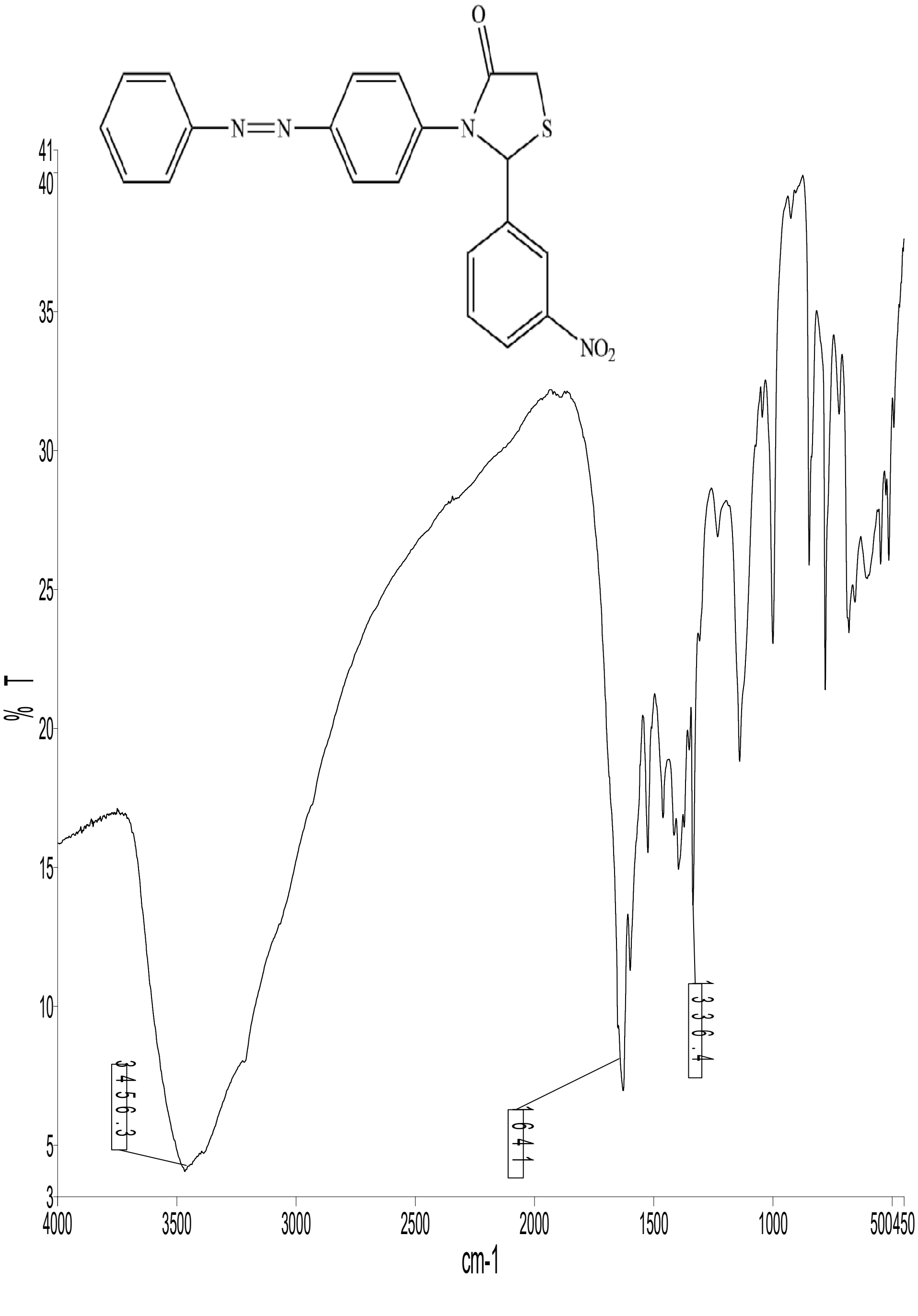

٤-۳۳- تهیه ی ترکیب ۲-(۳-نیترو فنیل)-۳-(٤-فنیل دیازنیل)فنیل)تیازولیدین-٤-اون (٤i)

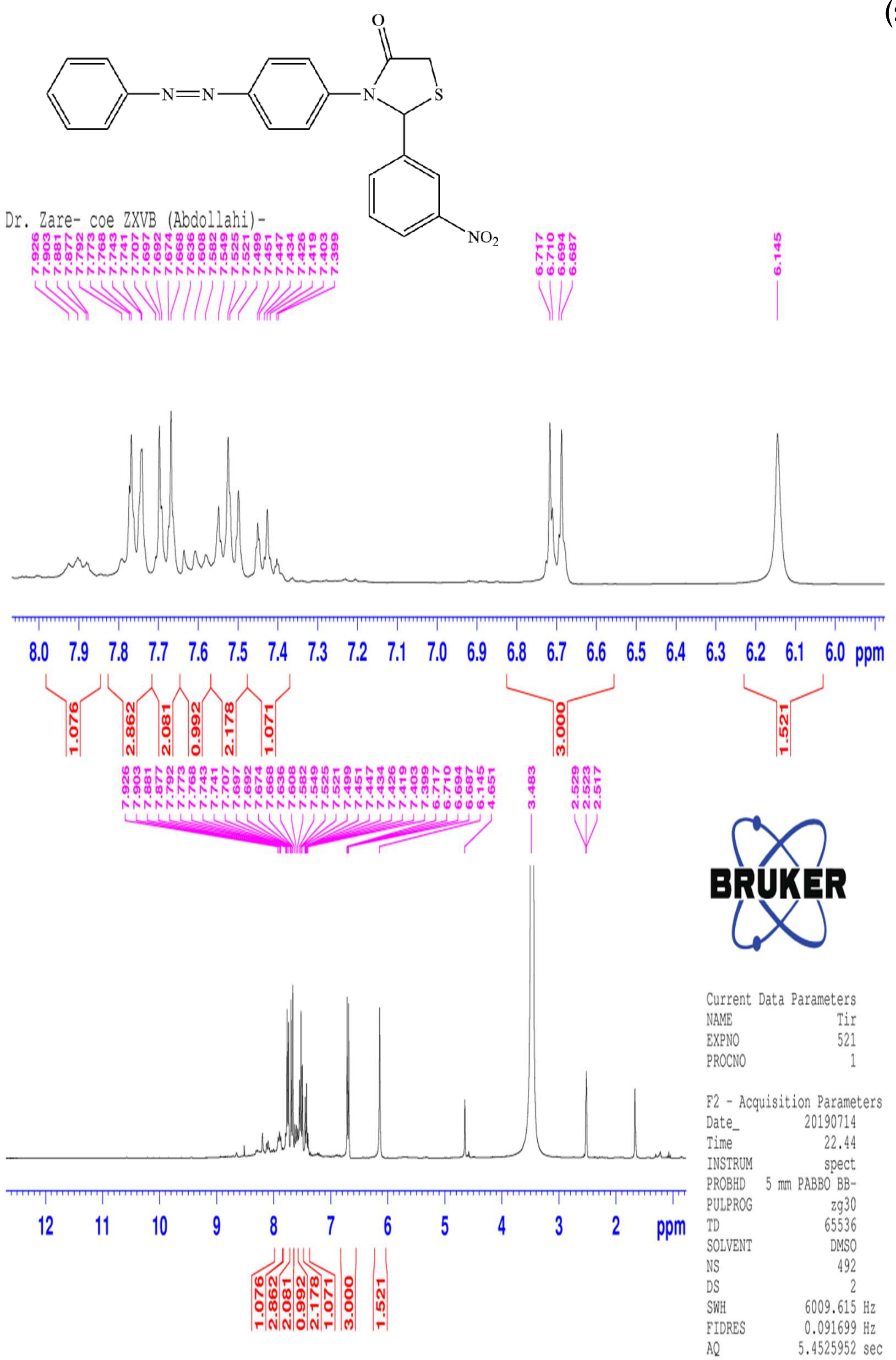

٤-٣٤- تهیه ی ترکیب ۲-(۳-نیترو فنیل)-۳-(٤-فنیل دیازنیل)فنیل)تیازولیدین-٤-اون (٤i)

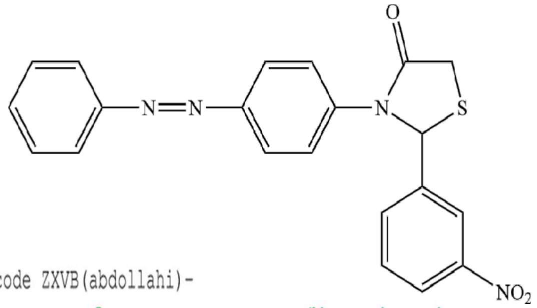

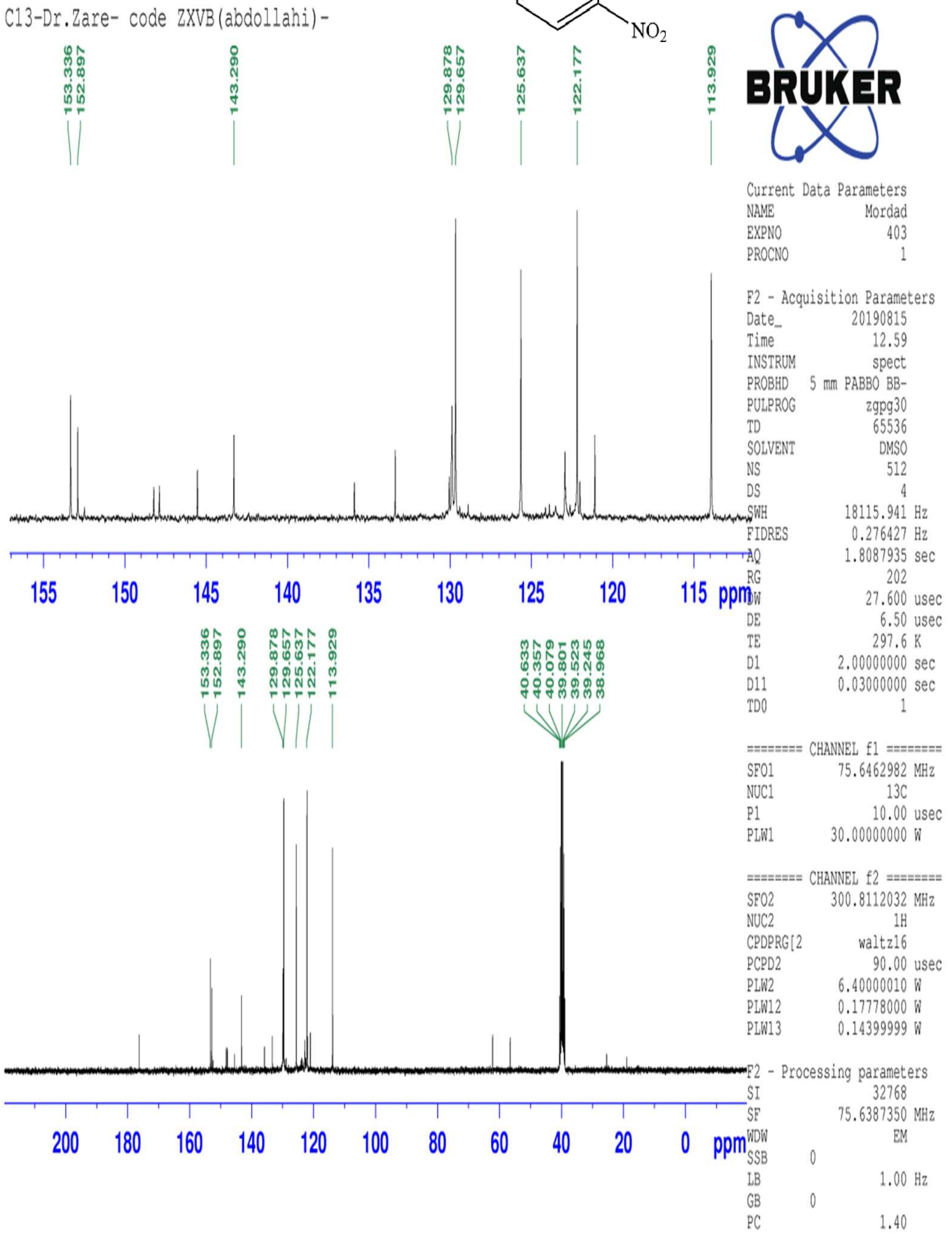

٤-٣٥- تهیه ی ترکیب ٢-(٣-برمو فنیل)-٣-(٤-فنیل دیازنیل)فنیل)تیازولیدین-٤-اون (٤j)

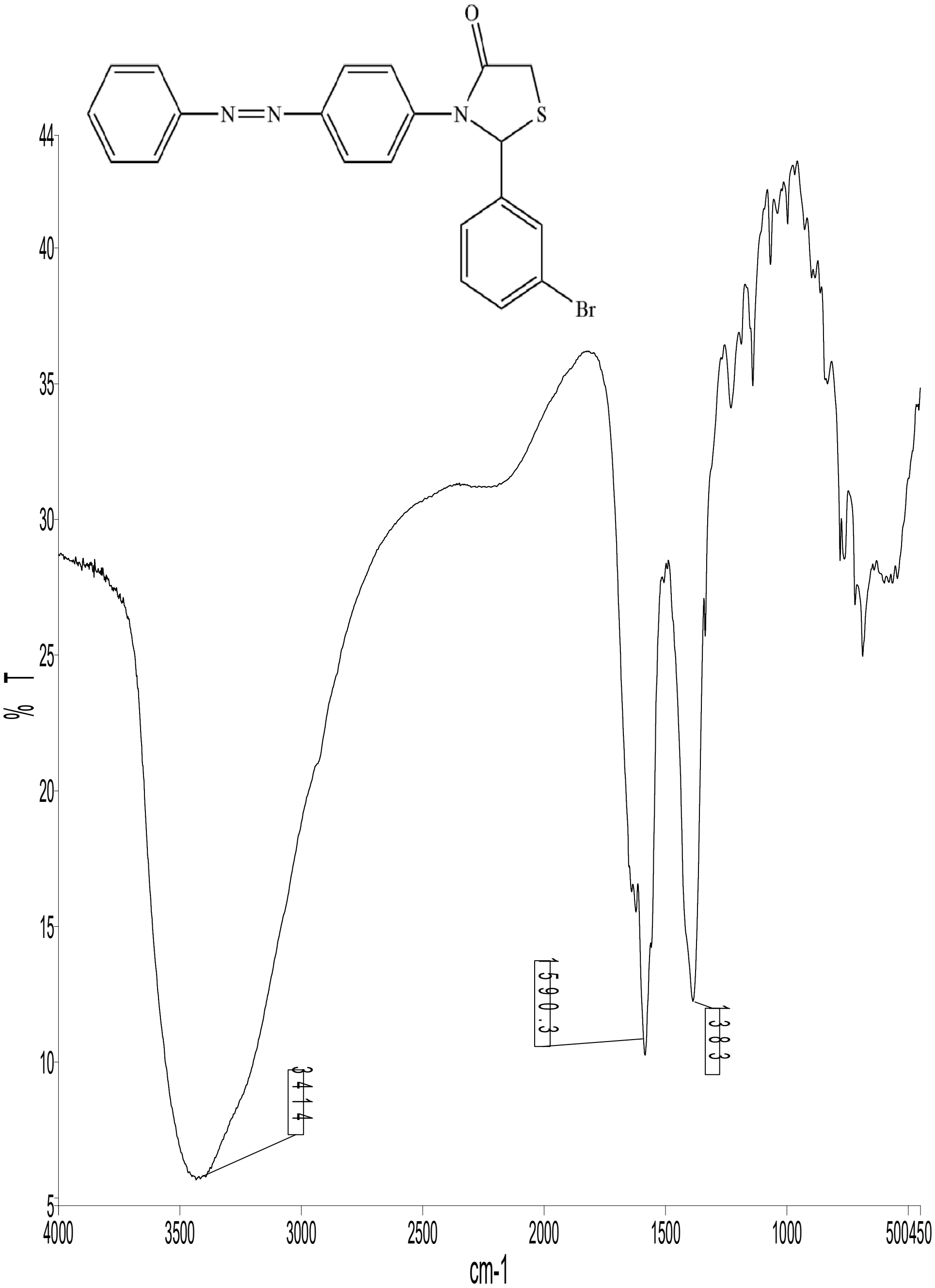

٤-٣٦- تهیه ی ترکیب ۲-(۳-برمو فنیل)-۳-(٤-فنیل دیازنیل)فنیل)تیازولیدین-٤-اون (٤j)

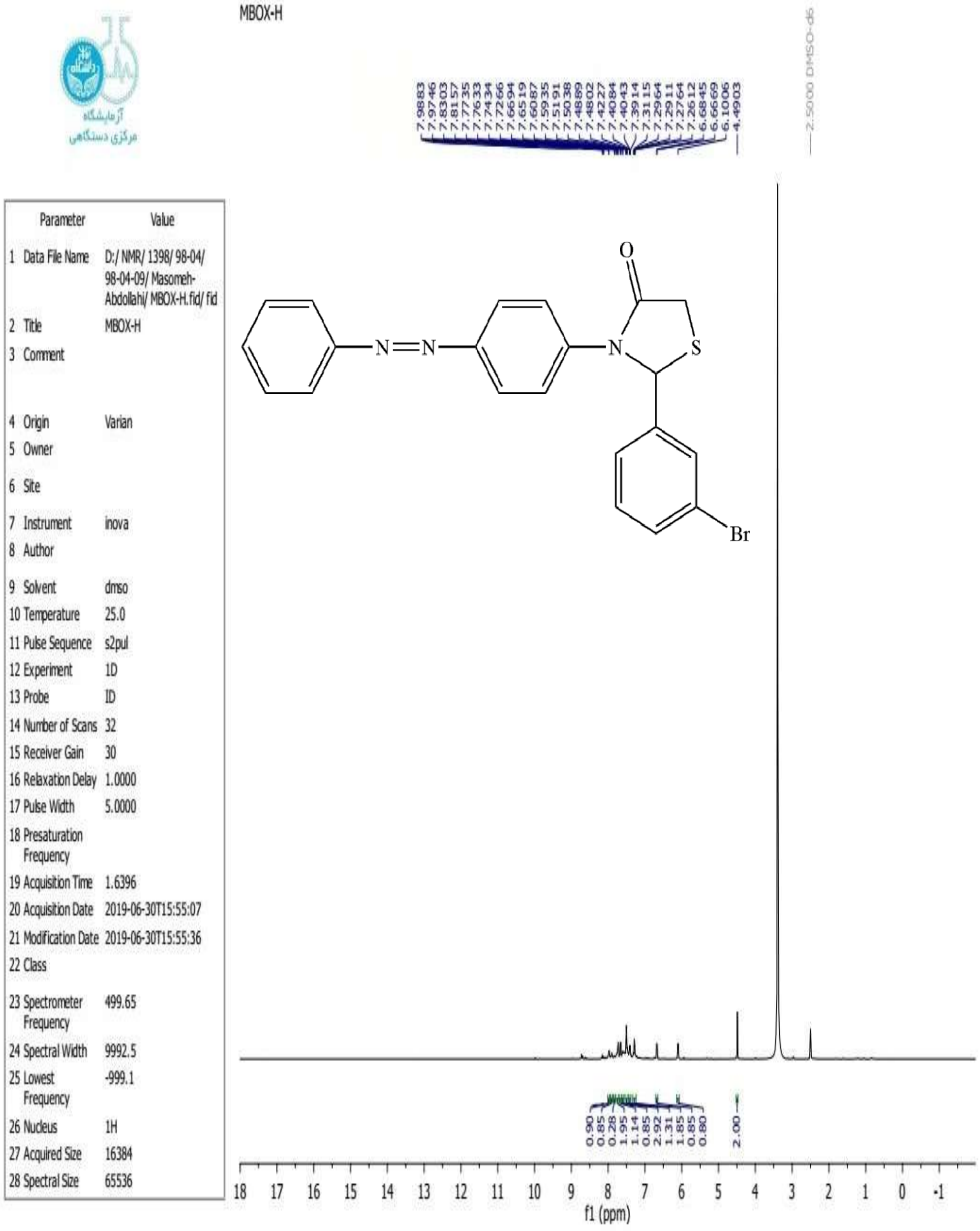

٤-٣٧- تهیه ی ترکیب ۲-(۳-برمو فنیل)-۳-(٤-فنیل دیازنیل)فنیل)تیازولیدین-٤-اون (٤j)

Parameter	Value
1 Data File Name	D:/ NMR/ 1398/ 98-04/ 98-04-09/ Masomeh-Abdollahi/ MBOX-H.fid/ fid
2 Title	MBOX-H
3 Comment	
4 Origin	Varian
5 Owner	
6 Site	
7 Instrument	inova
8 Author	
9 Solvent	dmso
10 Temperature	25.0
11 Pulse Sequence	s2pul
12 Experiment	1D
13 Probe	ID
14 Number of Scans	32
15 Receiver Gain	30
16 Relaxation Delay	1.0000
17 Pulse Width	5.0000
18 Presaturation Frequency	
19 Acquisition Time	1.6396
20 Acquisition Date	2019-06-30T15:55:07
21 Modification Date	2019-06-30T15:55:36
22 Class	
23 Spectrometer Frequency	499.65
24 Spectral Width	9992.5
25 Lowest Frequency	-999.1
26 Nucleus	1H
27 Acquired Size	16384
28 Spectral Size	65536

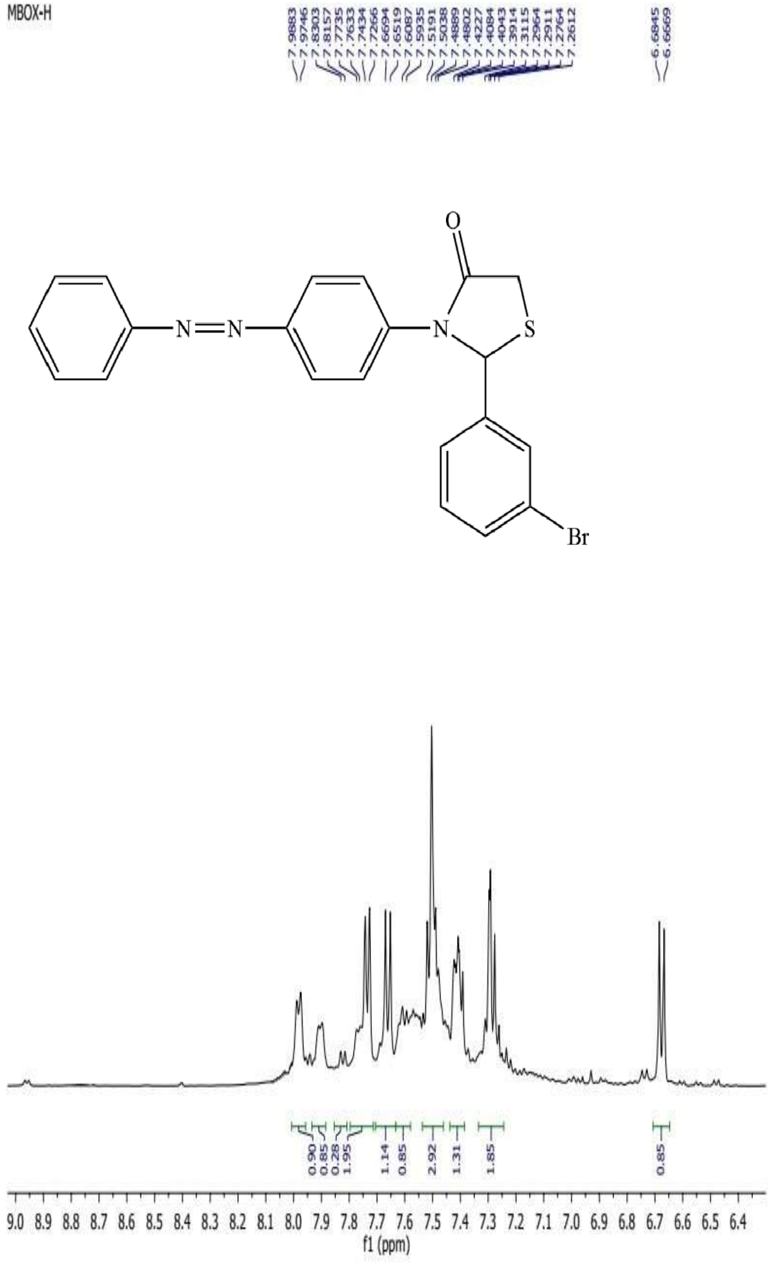

٤-٣٨- تهیه ی ترکیب ٢-(٣-برمو فنیل)-٣-(٤-فنیل دیازنیل)فنیل)تیازولیدین-٤-اون (٤j)

File : C:\MSDCHEM\3\DATA\Snapshot\30001820.D
Operator : taghizadeh
Acquired : 9 Jul 2019 14:52 using AcqMethod PAH
Instrument : Instrumen
Sample Name: MBOX
Misc Info :
Vial Number: 1

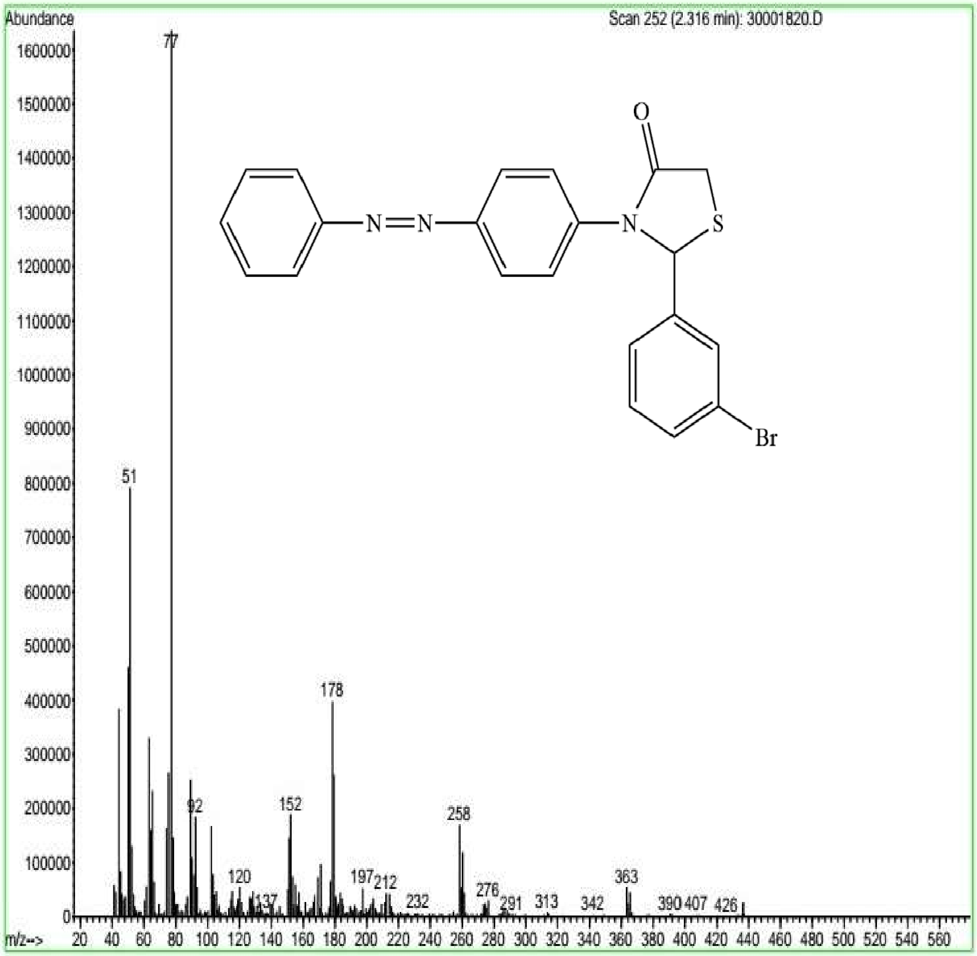

PDF Creator - PDF4Free v3.0 http://www.pdf4free.com

٤-۳۹- تهیه ی ترکیب ۲-(۲-نیترو فنیل)-۳-(٤-فنیل دیازنیل)فنیل)تیازولیدین-٤-اون (٤k)

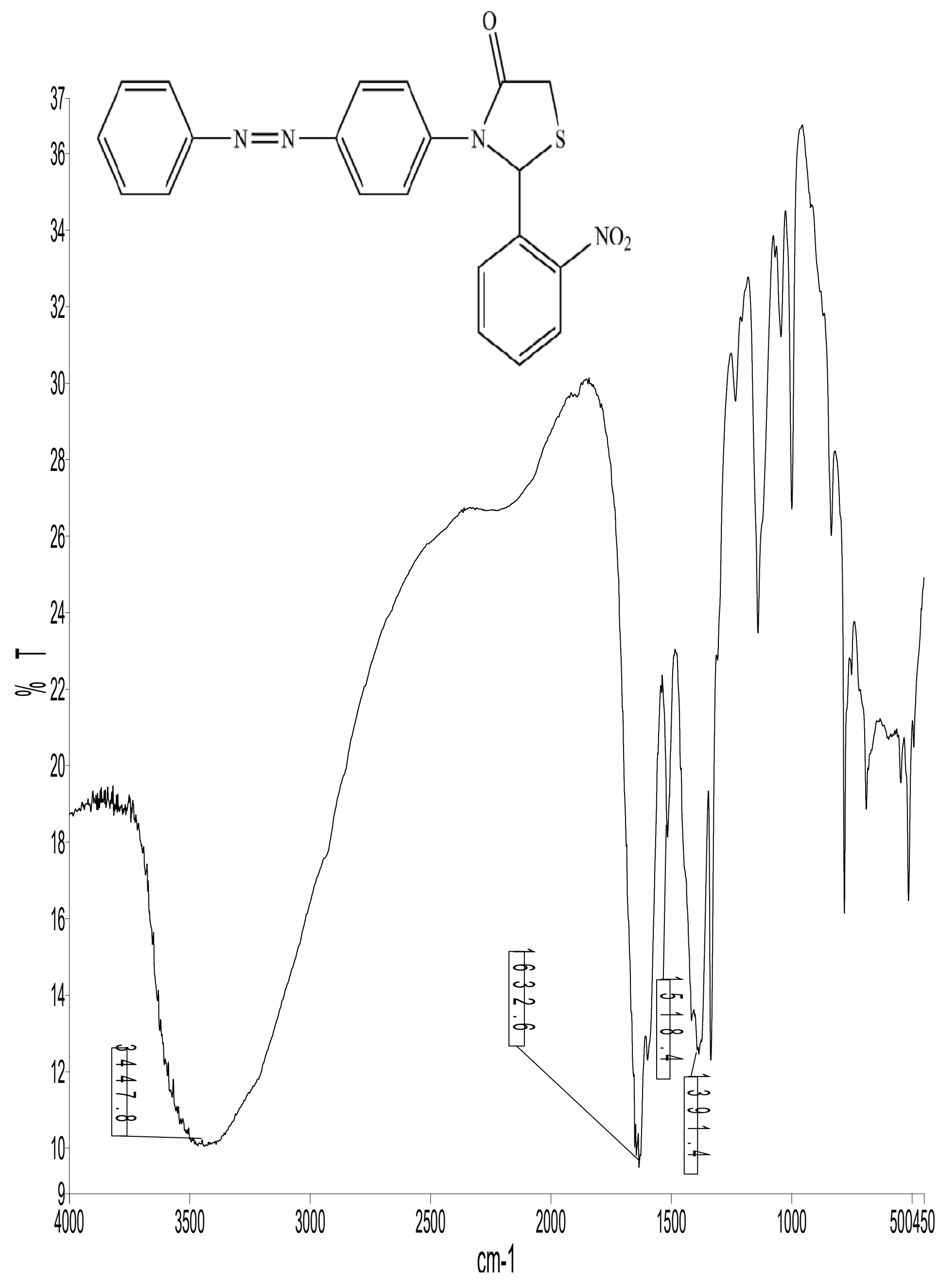

٤-٤٠- تهیه ی ترکیب ۲-(۲-نیترو فنیل)-۳-(٤-فنیل دیازنیل)فنیل)تیازولیدین-٤-اون (٤k)

Parameter		Value
1	Data File Name	D:/ NMR/ 1398/ 98-04/ 98-04-09/ Masomeh-Abdollahi/ RTYP-H.fid/ fid
2	Title	RTYP-H
3	Comment	
4	Origin	Varian
5	Owner	
6	Site	
7	Instrument	inova
8	Author	
9	Solvent	dmso
10	Temperature	25.0
11	Pulse Sequence	s2pul
12	Experiment	1D
13	Probe	ID
14	Number of Scans	16
15	Receiver Gain	30
16	Relaxation Delay	1.0000
17	Pulse Width	5.0000
18	Presaturation Frequency	
19	Acquisition Time	1.6396
20	Acquisition Date	2019-06-30T15:44:05
21	Modification Date	2019-06-30T15:44:46
22	Class	
23	Spectrometer Frequency	499.65
24	Spectral Width	9992.5
25	Lowest Frequency	-999.1
26	Nucleus	1H
27	Acquired Size	16384
28	Spectral Size	65536

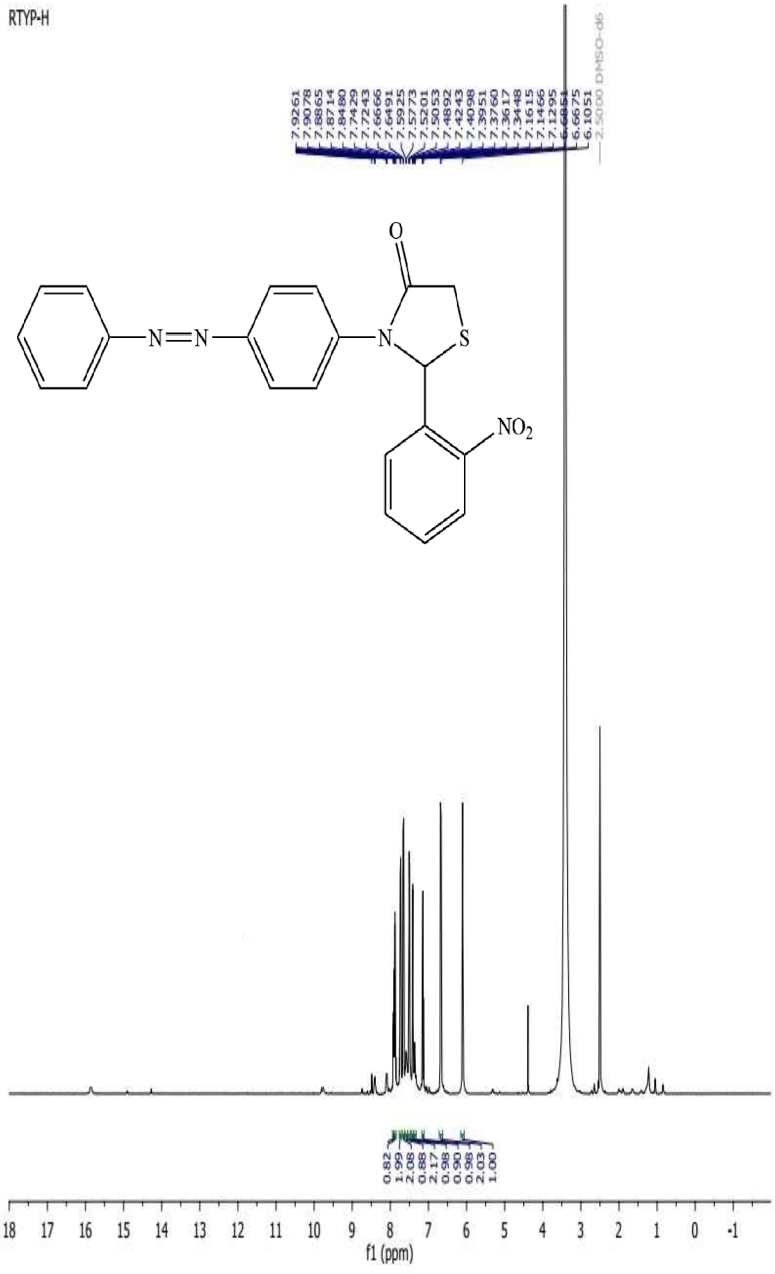

٤-٤١- تهیه ی ترکیب ۲-(۲-نیترو فنیل)-۳-(٤-فنیل دیازنیل)فنیل)تیازولیدین-٤-اون (٤k)

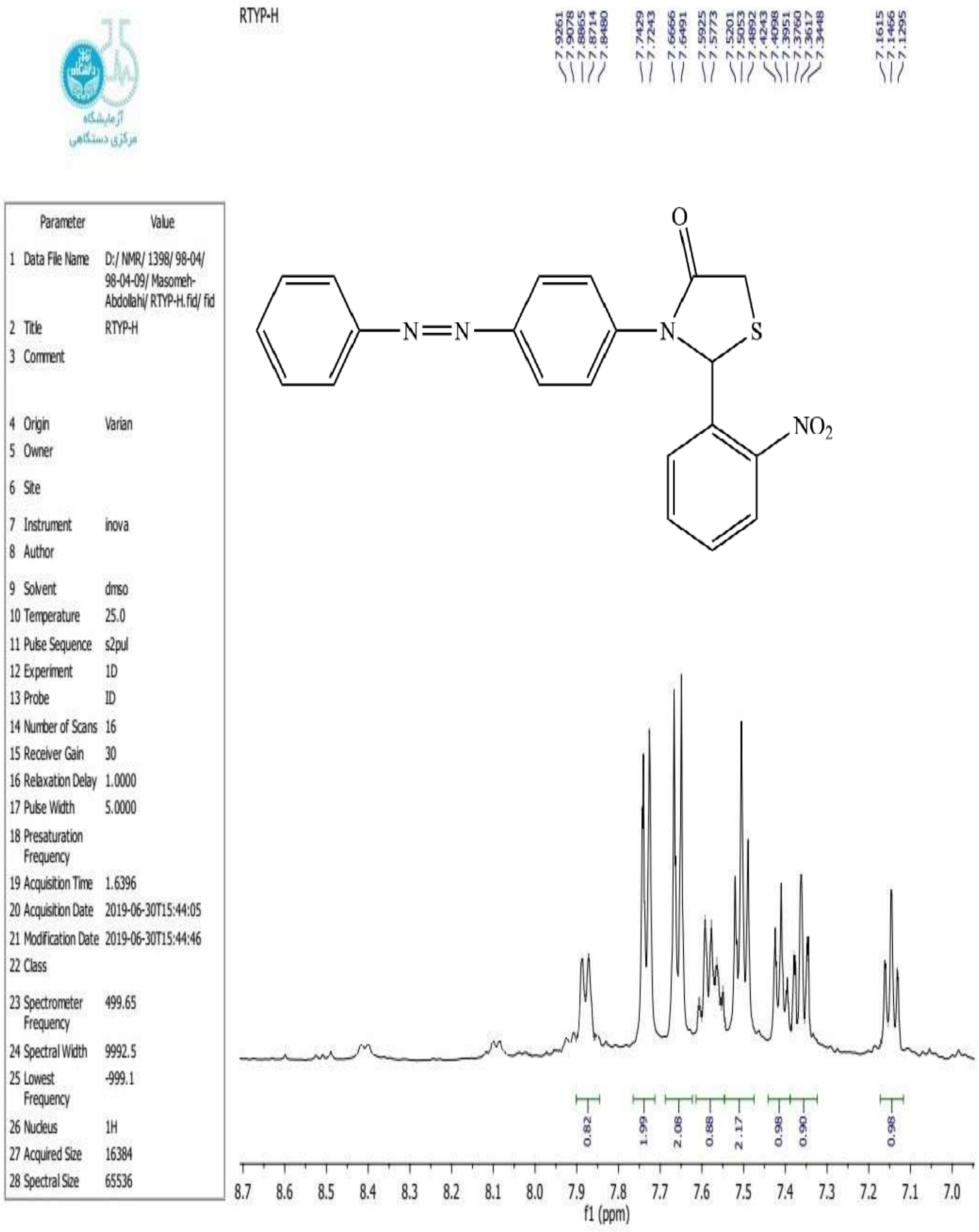

٤-٤۲- تهیه ی ترکیب ۲-(۲-نیترو فنیل)-۳-(٤-فنیل دیازنیل)فنیل)تیازولیدین-٤-اون (٤k)

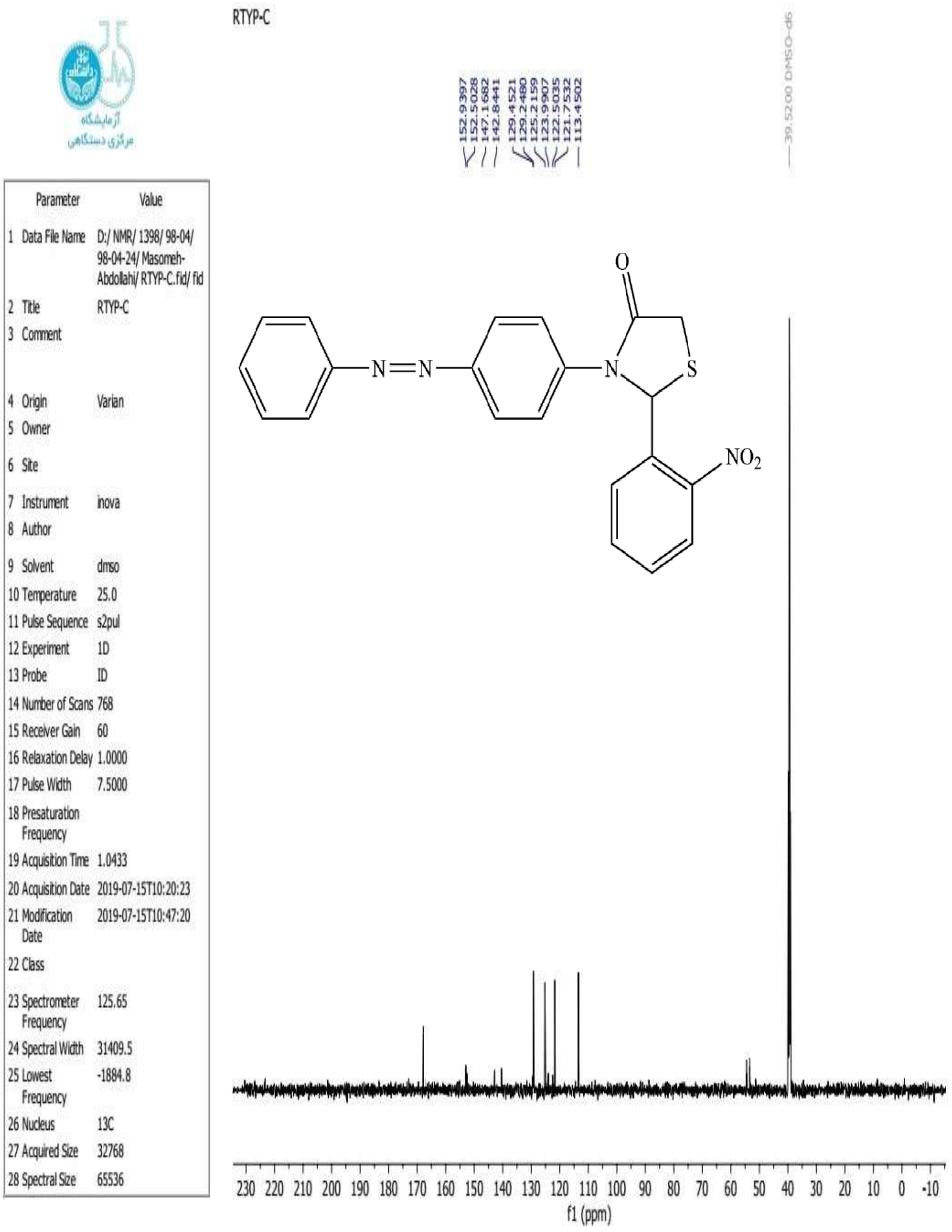

٤-٤٣- تهیه ی ترکیب ۲-(۲،٤-دی کلرو فنیل)-۳-(٤-فنیل دیازنیل)فنیل)تیازولیدین-٤-اون (٤l)

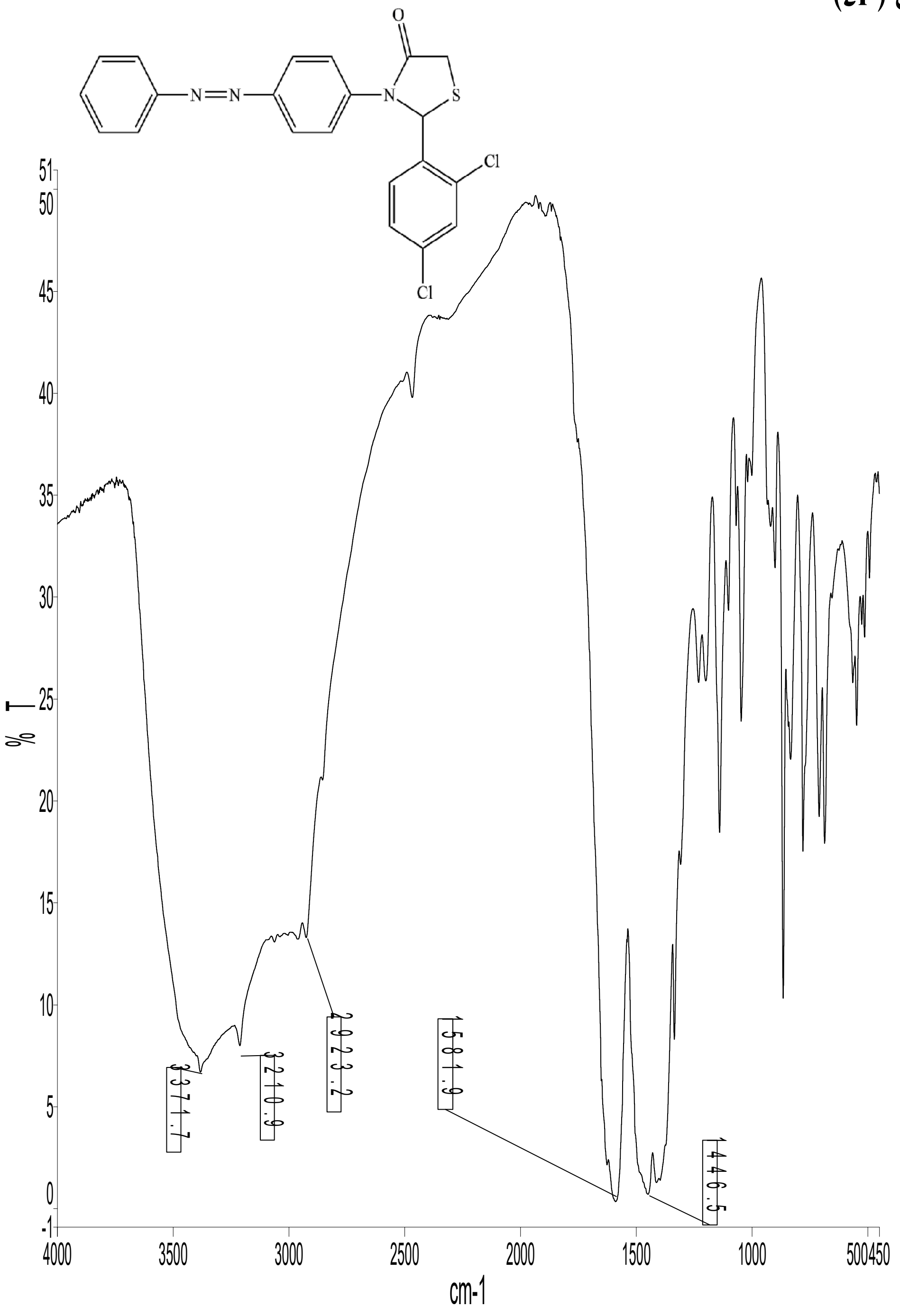

٤-٤٤- تهیه ی ترکیب ۲-(۲،٤-دی کلرو فنیل)-۳-(٤-فنیل دیازنیل)فنیل)تیازولیدین-٤-اون (٤l)

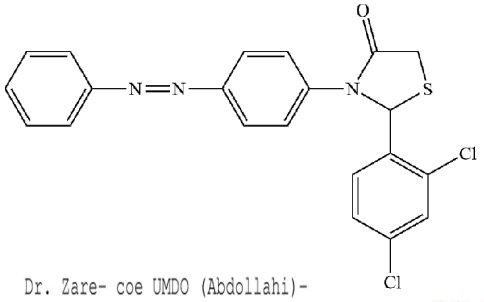

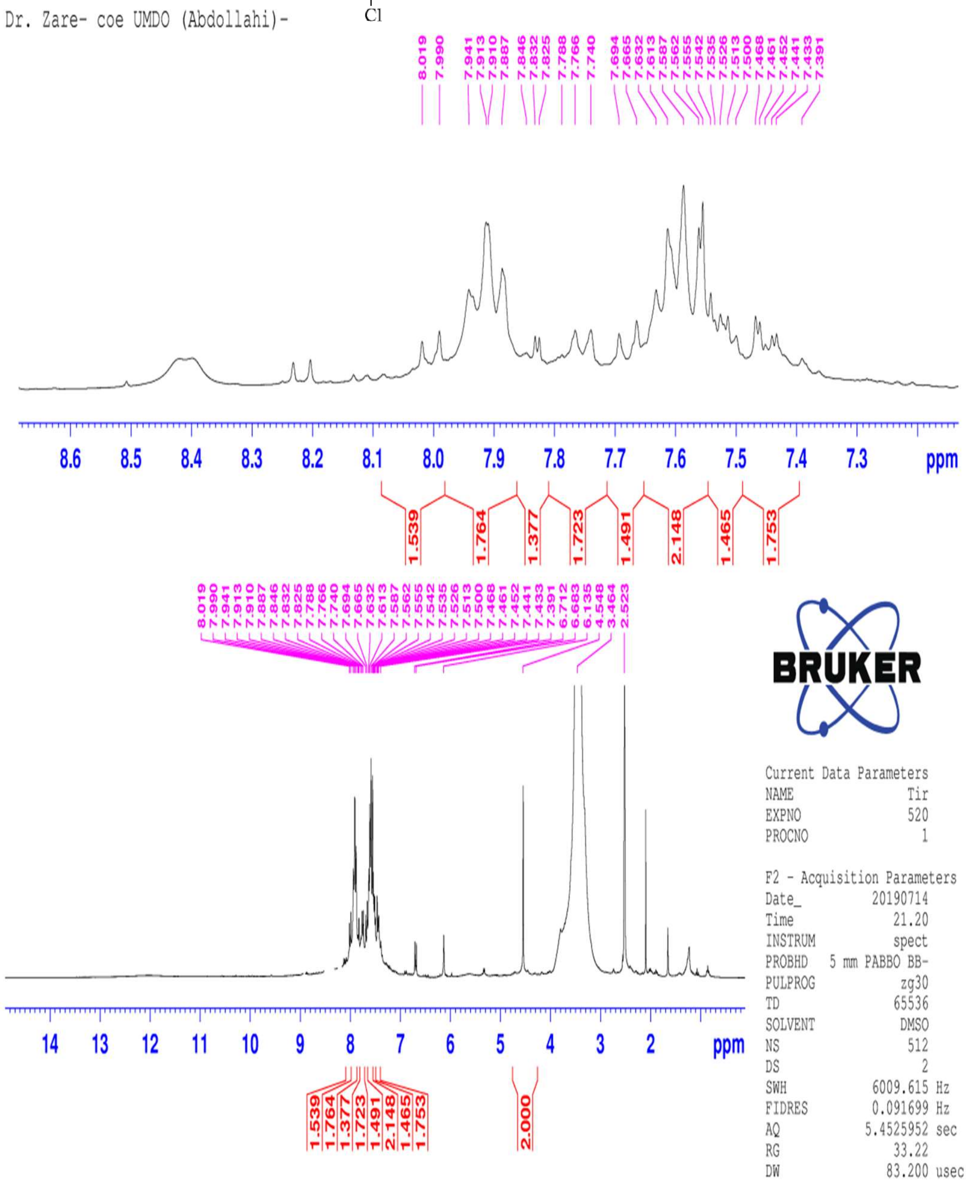

٤-٤٥- تهیه ی ترکیب ٢-(٢،٤-دی کلرو فنیل)-٣-(٤-فنیل دیازنیل)فنیل)تیازولیدین-٤-اون (٤l)

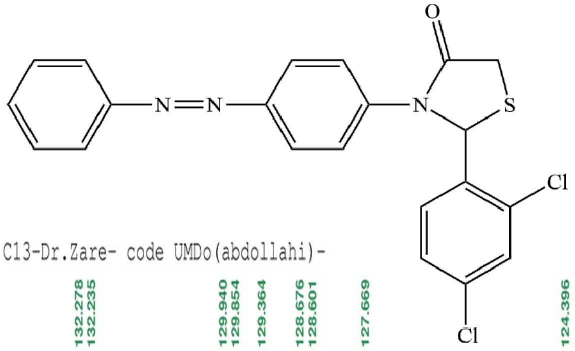

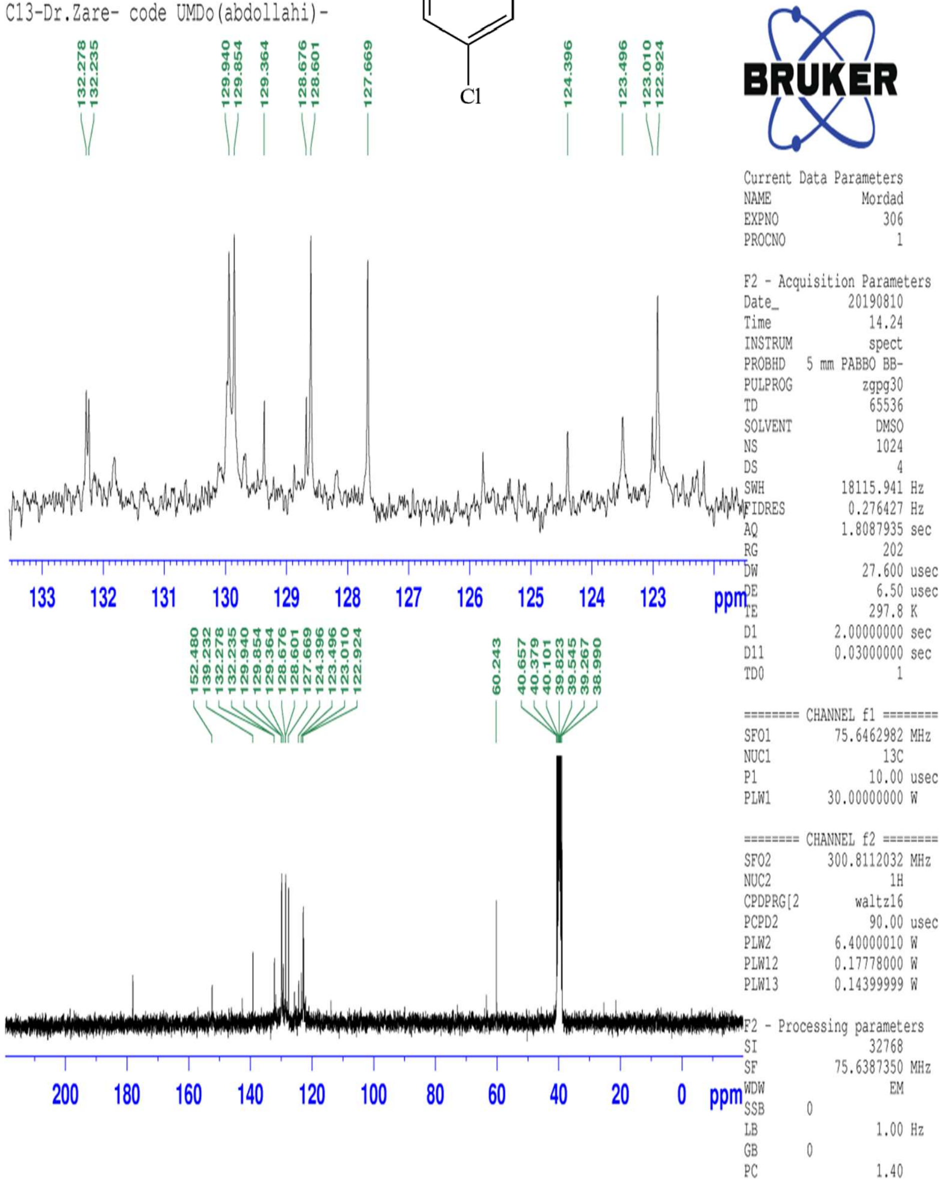

٤-٤٦- تهیه ی ترکیب ۲-(٤-هیدروکسی-۳-متوکسی فنیل)-۳-(٤-فنیل دیازنیل)فنیل) تیازولیدین-٤-اون (٤m)

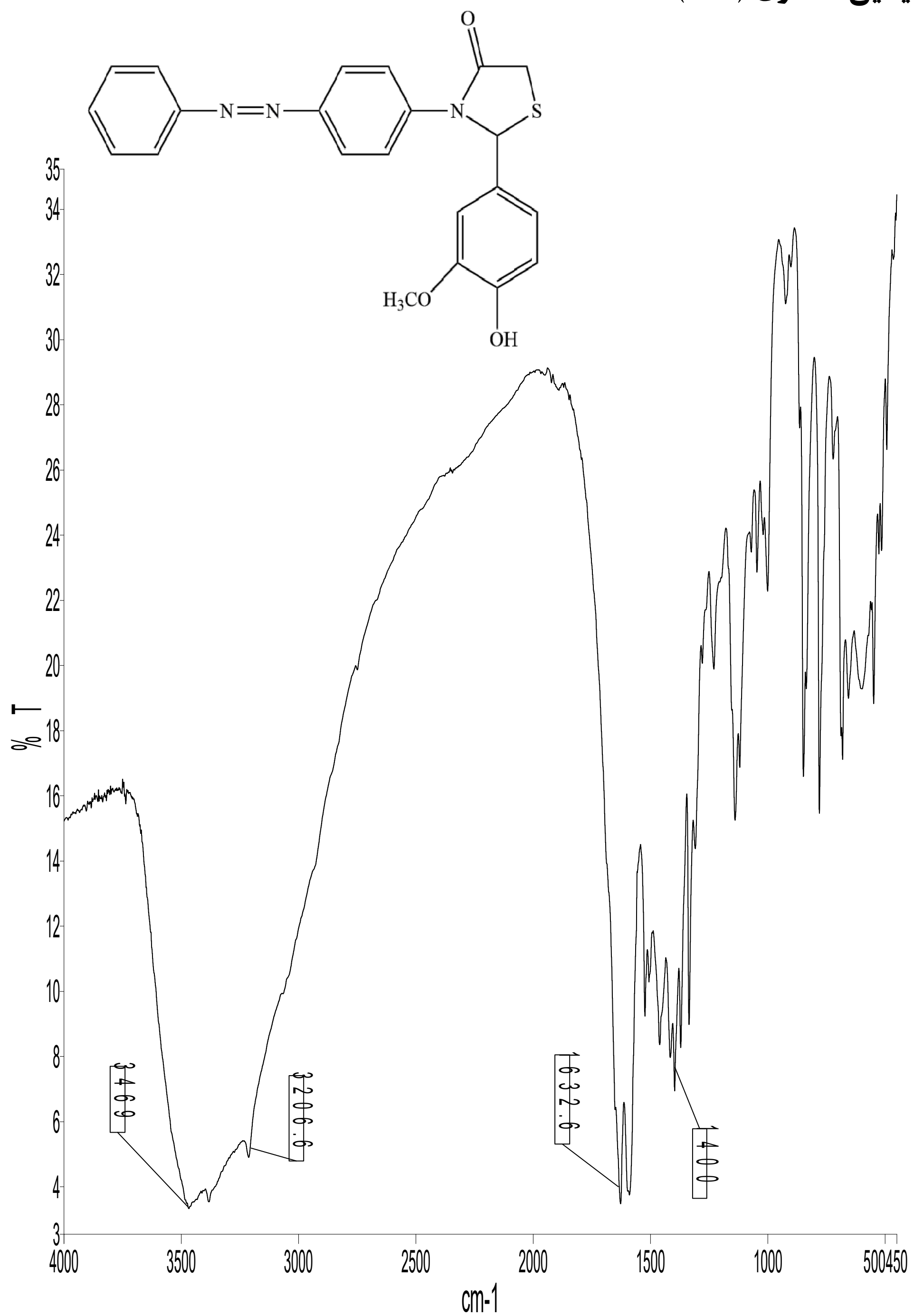

٤-٤٧- تهیه ی ترکیب ۲-(٤-هیدروکسی-۳-متوکسی فنیل)-۳-(٤-فنیل دیازنیل)فنیل) تیازولیدین-٤-اون (٤m)

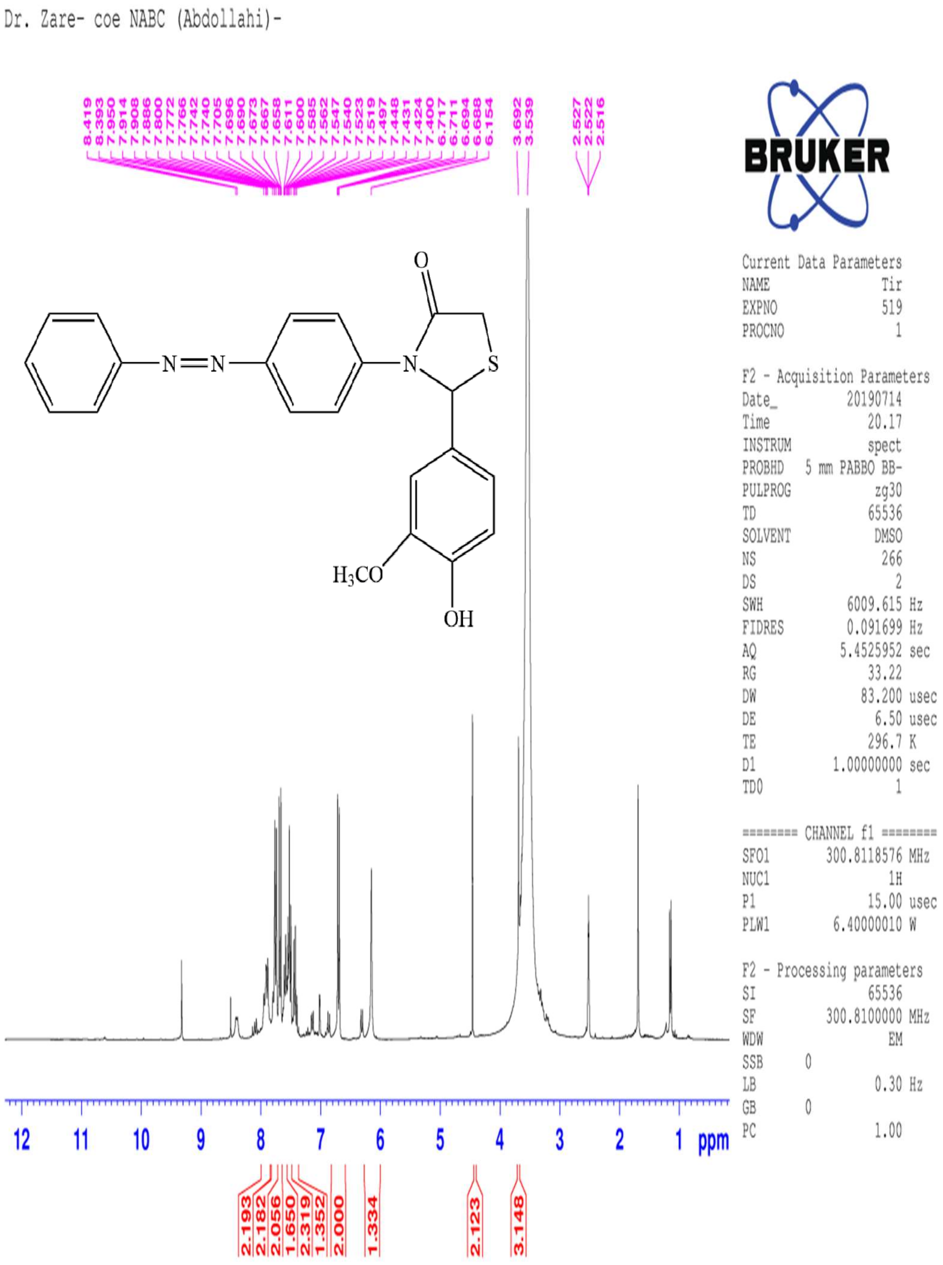

٤-٤٨- تهیه ی ترکیب ۲-(٤-هیدروکسی-۳-متوکسی فنیل)-۳-(٤-فنیل دیازنیل)فنیل) تیازولیدین-٤-اون (٤m)

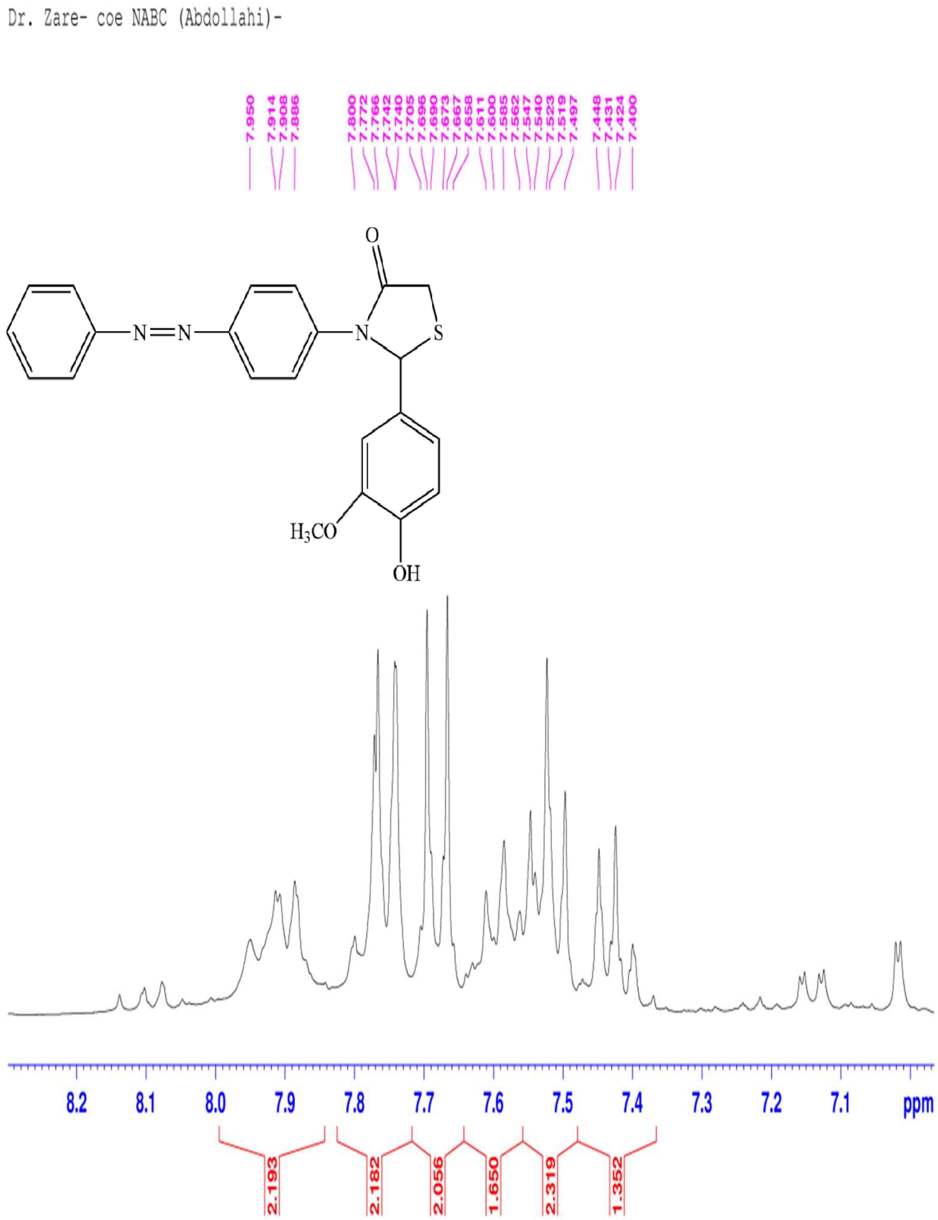

٤-٤٩- تهیه ی ترکیب ٢-(٤-هیدروکسی-٣-متوکسی فنیل)-٣-(٤-(٤-فنیل دیازنیل)فنیل) تیازولیدین-٤-اون (٤m)

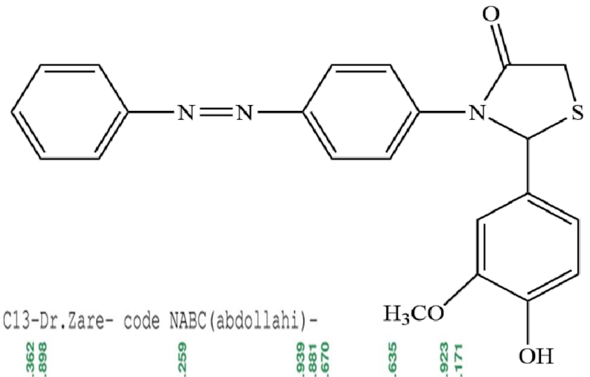

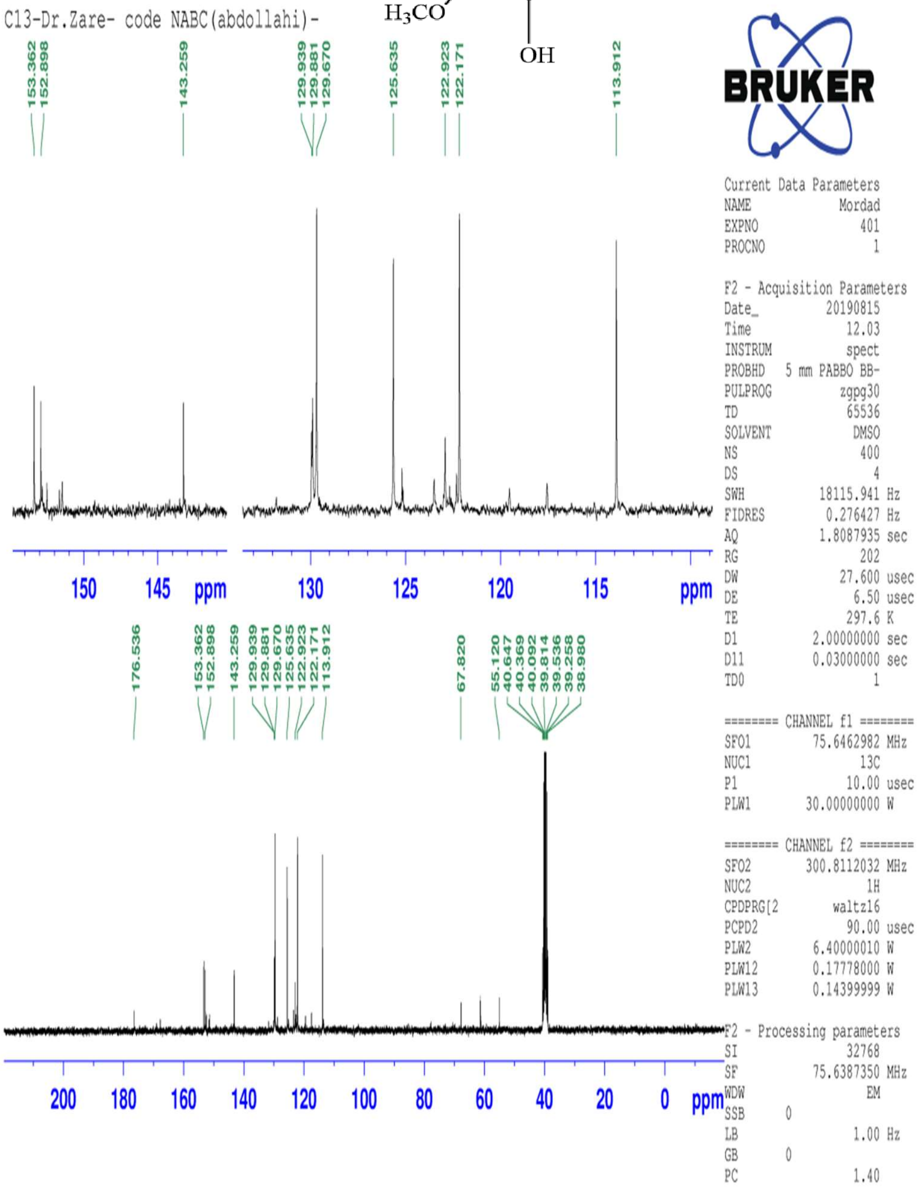

طیف FT-IR ۲-[۳-(۴-کلرو-فنیل)-۱-فنیل-*H*۱-پیرازول-۴-ایل]-۳-(۴-متوکسی-فنیل)-تیازولیدین-۴-اون(4a)

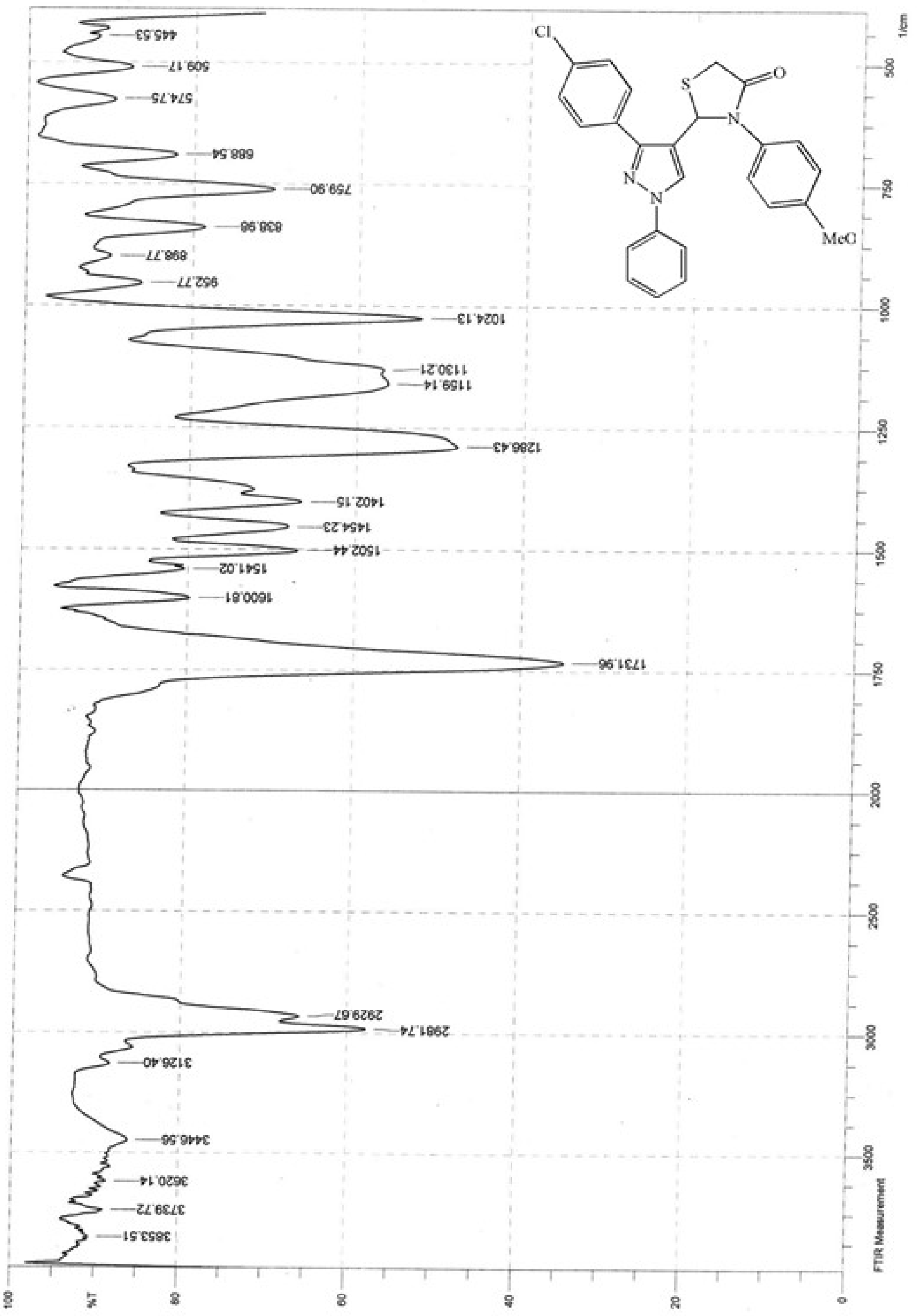

طیف ^{1}H NMR ۲-[۳-(۴-کلرو-فنیل)-۱-فنیل-۱*H*-پیرازول-۴-ایل]-۳-(۴-متوکسی-فنیل)-تیازولیدین-۴-اون(۴a)

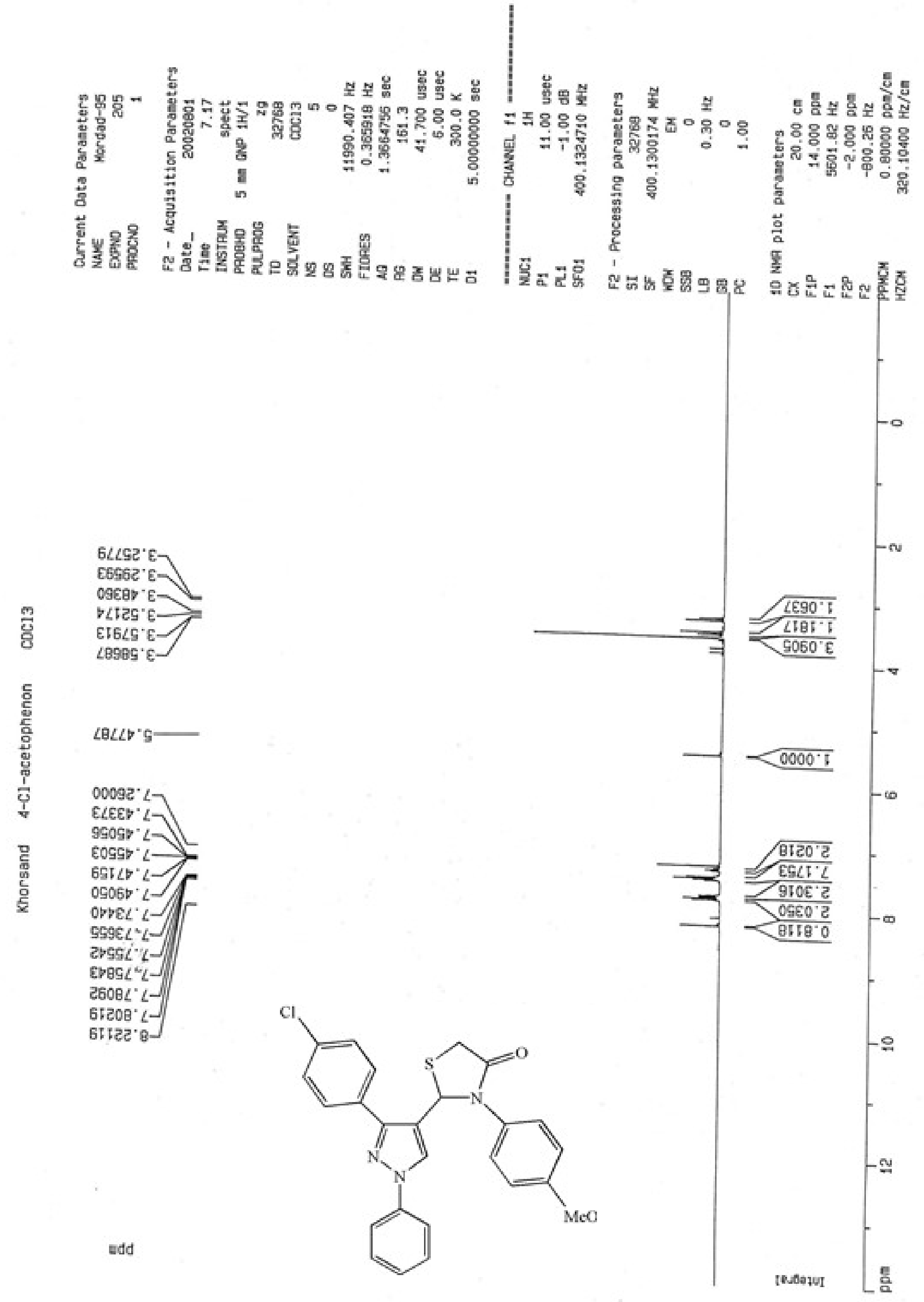

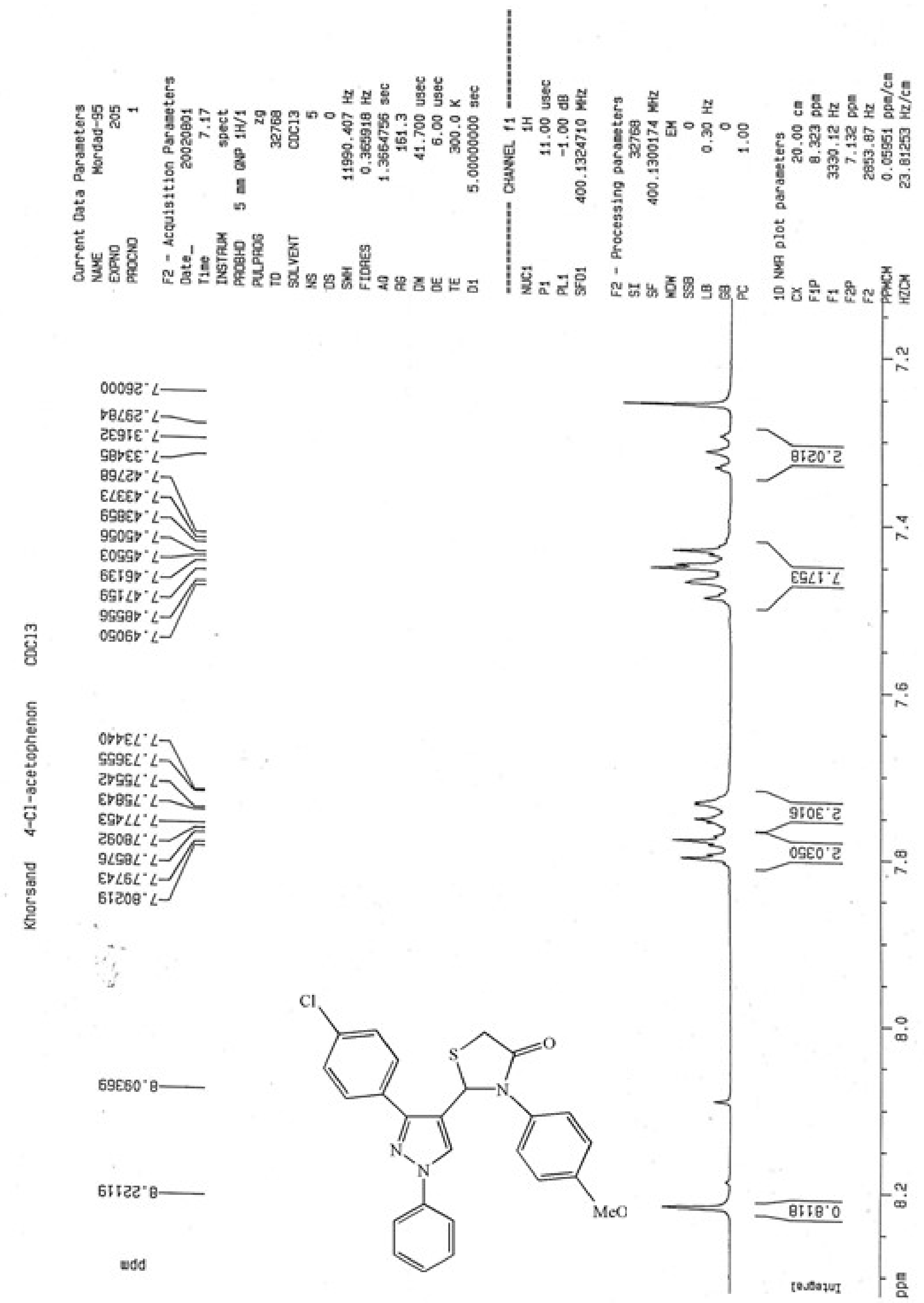
Khorsand 4-Cl-acetophenon CDCl3
8.22119
8.09369
7.80219
7.79743
7.78576
7.78092
7.77453
7.75843
7.75542
7.73655
7.73440
7.49050
7.48556
7.47159
7.46139
7.45503
7.45056
7.43859
7.43373
7.42768
7.33485
7.31632
7.29784
7.26000
0.8118
2.0350
2.3016
7.1753
2.0218
Integral
ppm

طیف ^{13}C NMR ۲-[۳-(۴-کلرو-فنیل)-۱-فنیل-۱*H*-پیرازول-۴-ایل]-۳-(۴-متوکسی-فنیل)-تیازولیدین-۴-اون(۴a)

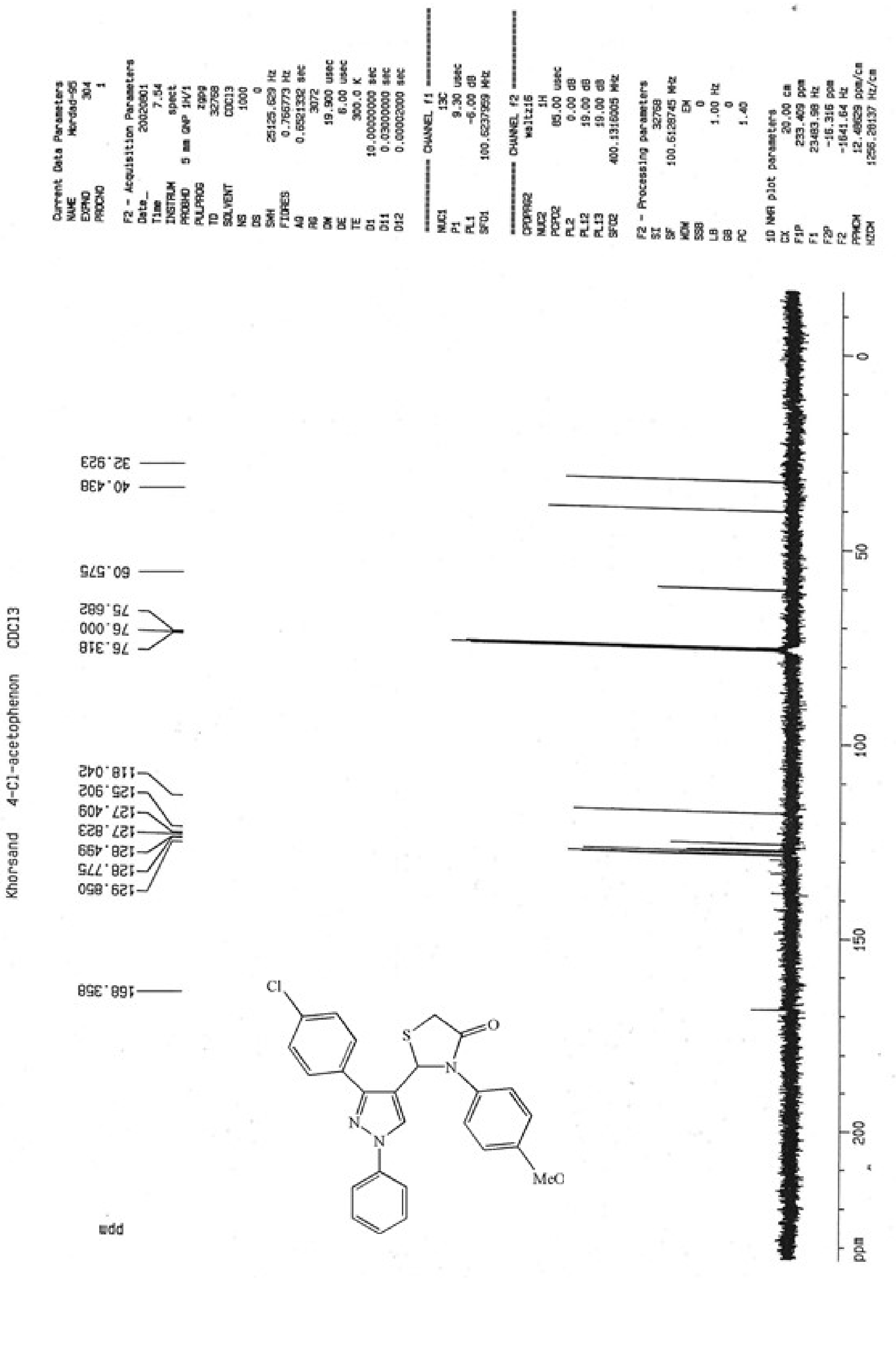

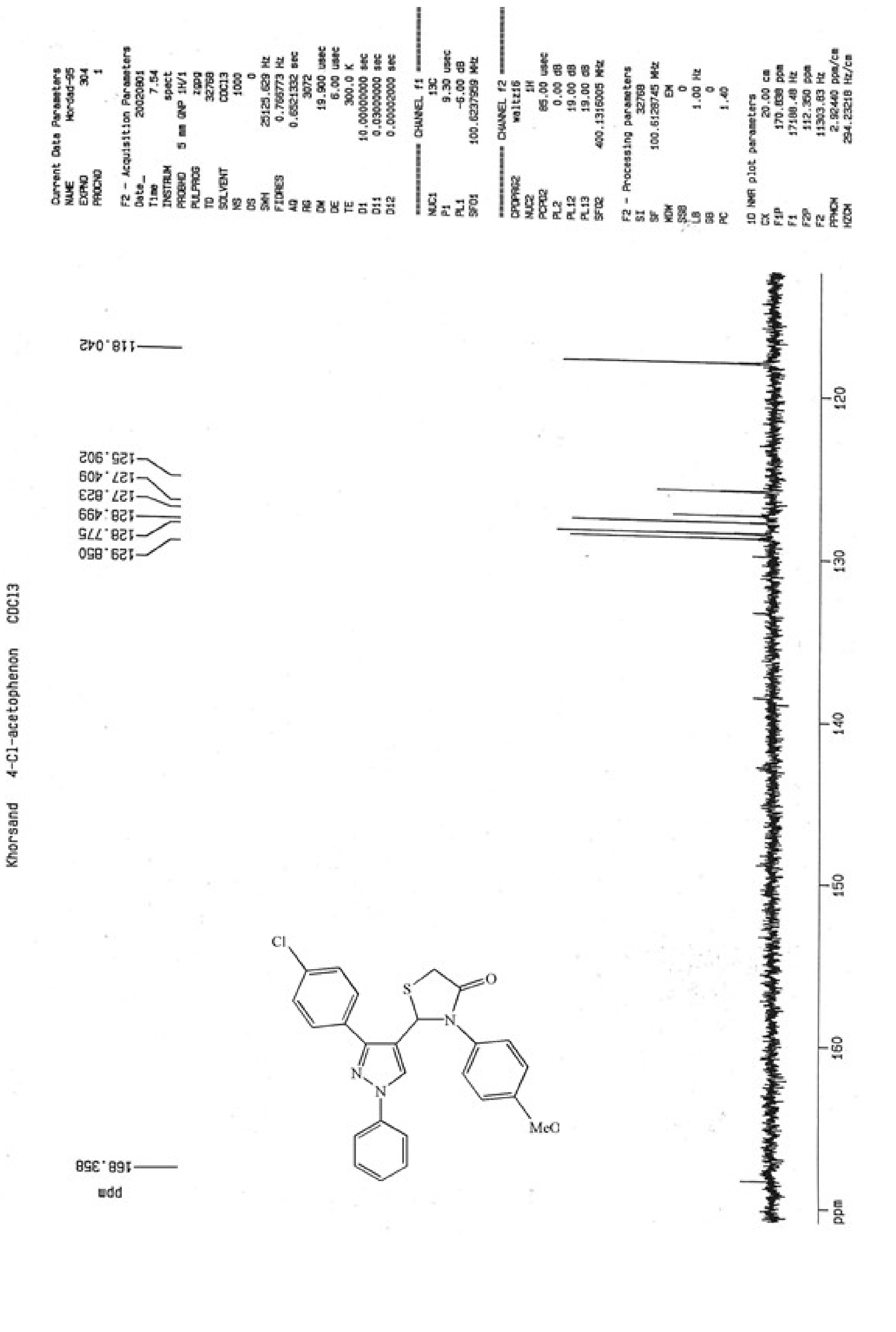
Khorsand 4-Cl-acetophenon CDCl3
118.042
125.902
127.409
127.823
128.499
128.775
129.850
168.358
ppm
Cl
S
O
N
N
N
MeO
120
130
140
150
160
ppm

طیف FT-IR ۲-(۱، ۳-دی فنیل-۱*H*-پیرازول-۴-ایل)-۳-(۴-متوکسی فنیل)-تیازولیدین-۴-اون(۴b)

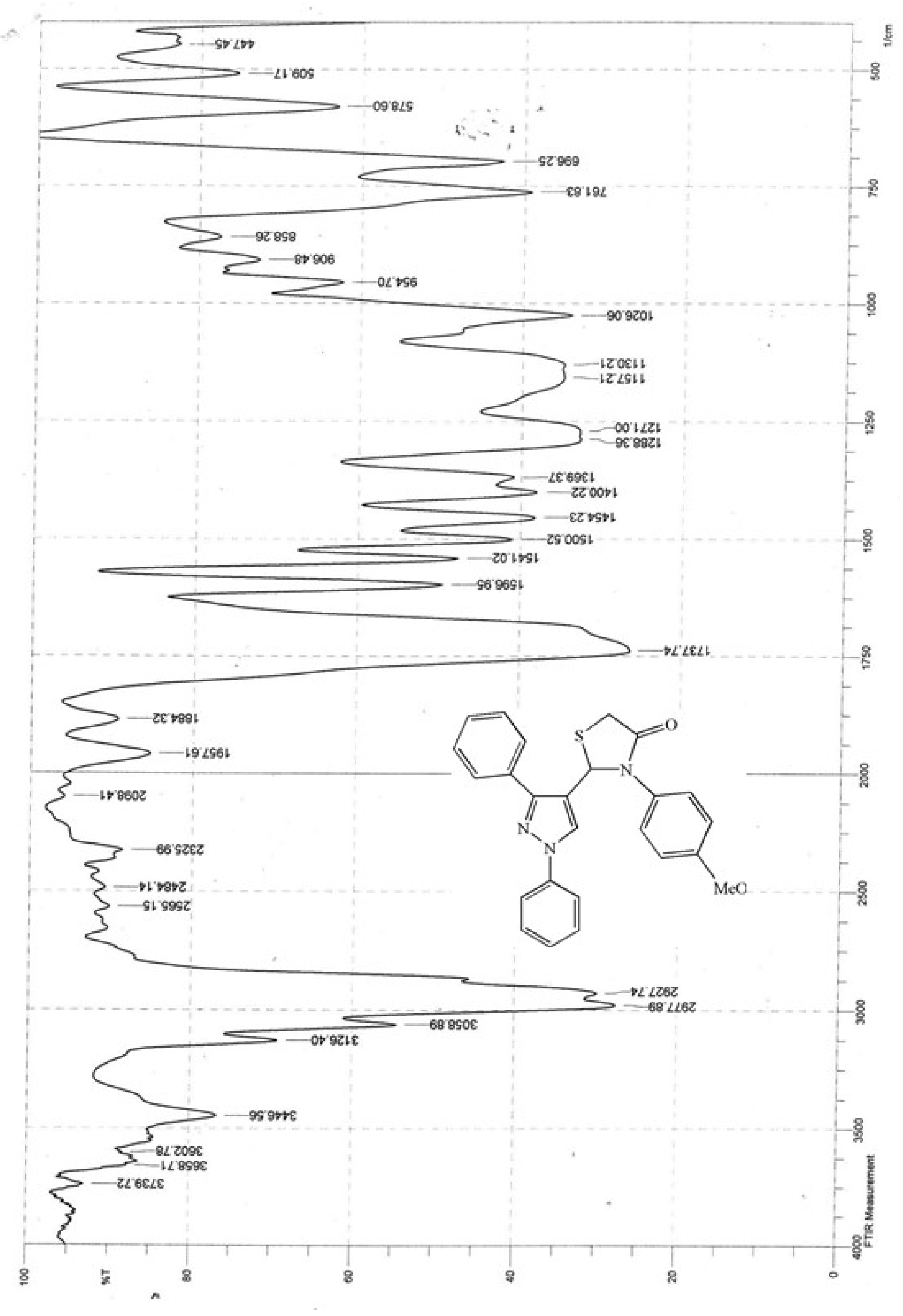

طیف ^{1}H NMR ۲-(۱، ۳-دی فنیل-۱*H*-پیرازول-۴-ایل)-۳-(۴-متوکسی فنیــل)-تیازولیــدین-۴-اون(۴b)

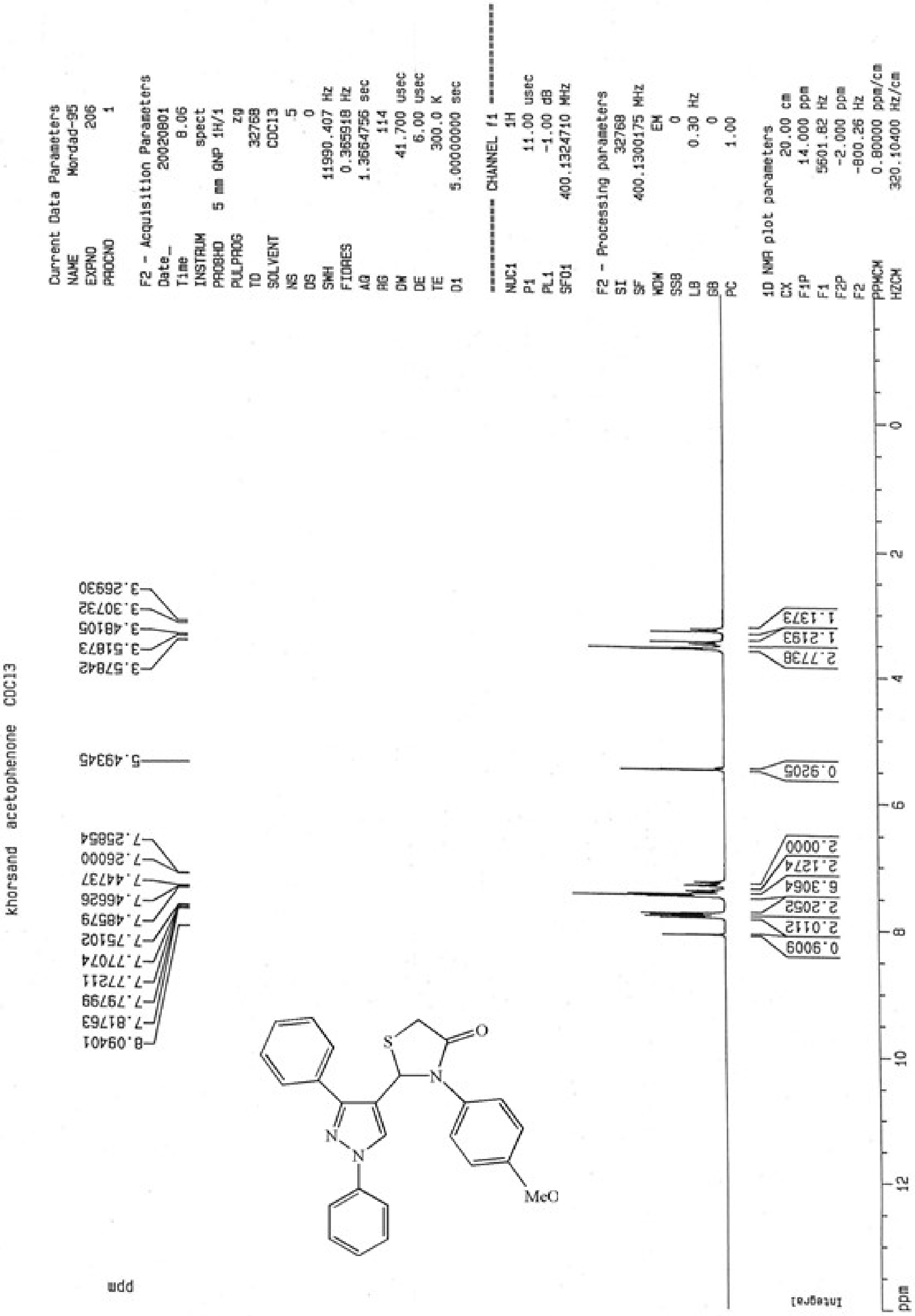

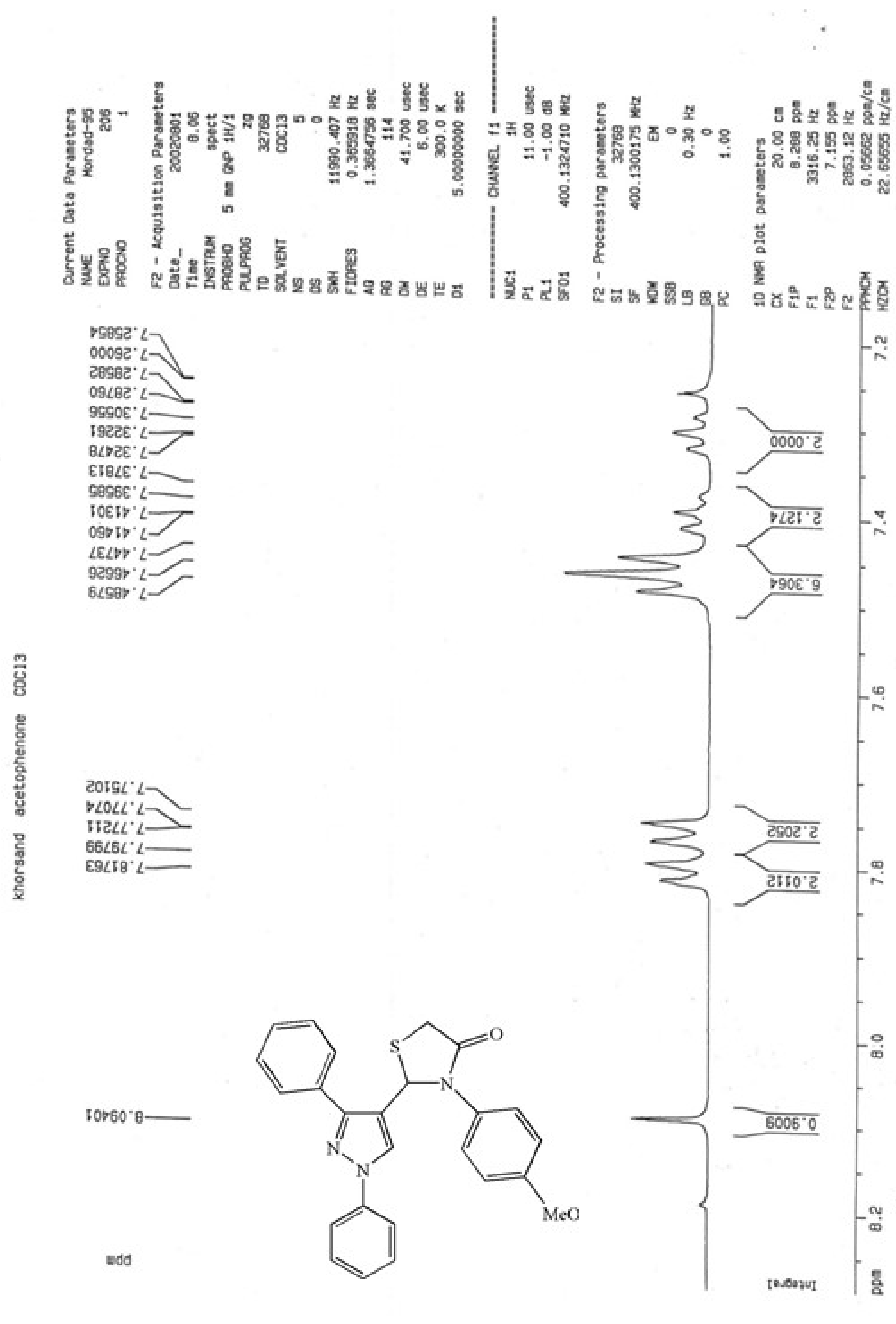

طیف ^{13}C NMR ۲-(۱، ۳-دی فنیل-$1H$-پیرازول-۴-ایل)-۳-(۴-متوکسی فنیل)-تیازولیــدین-۴-اون(۴b)

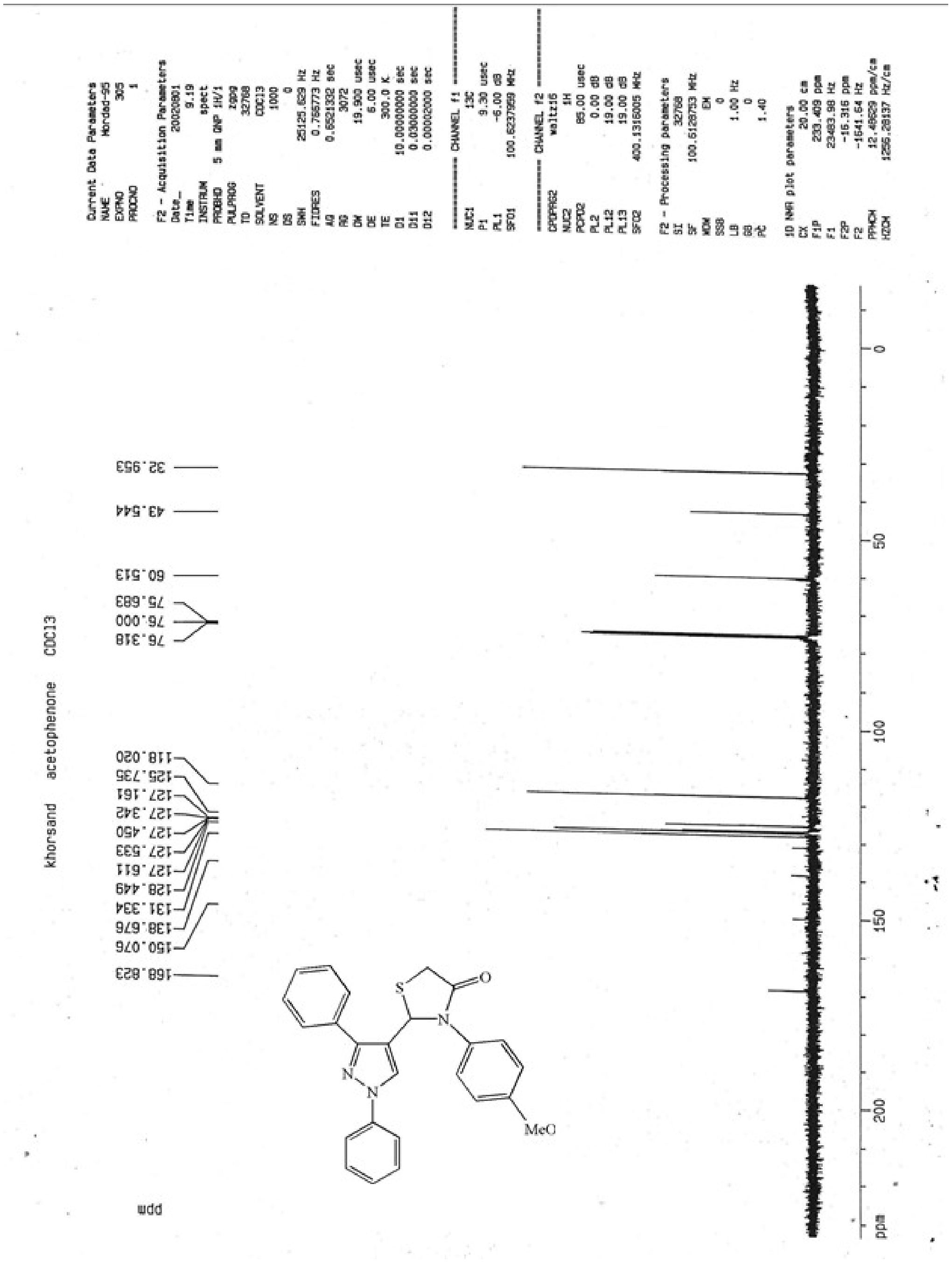

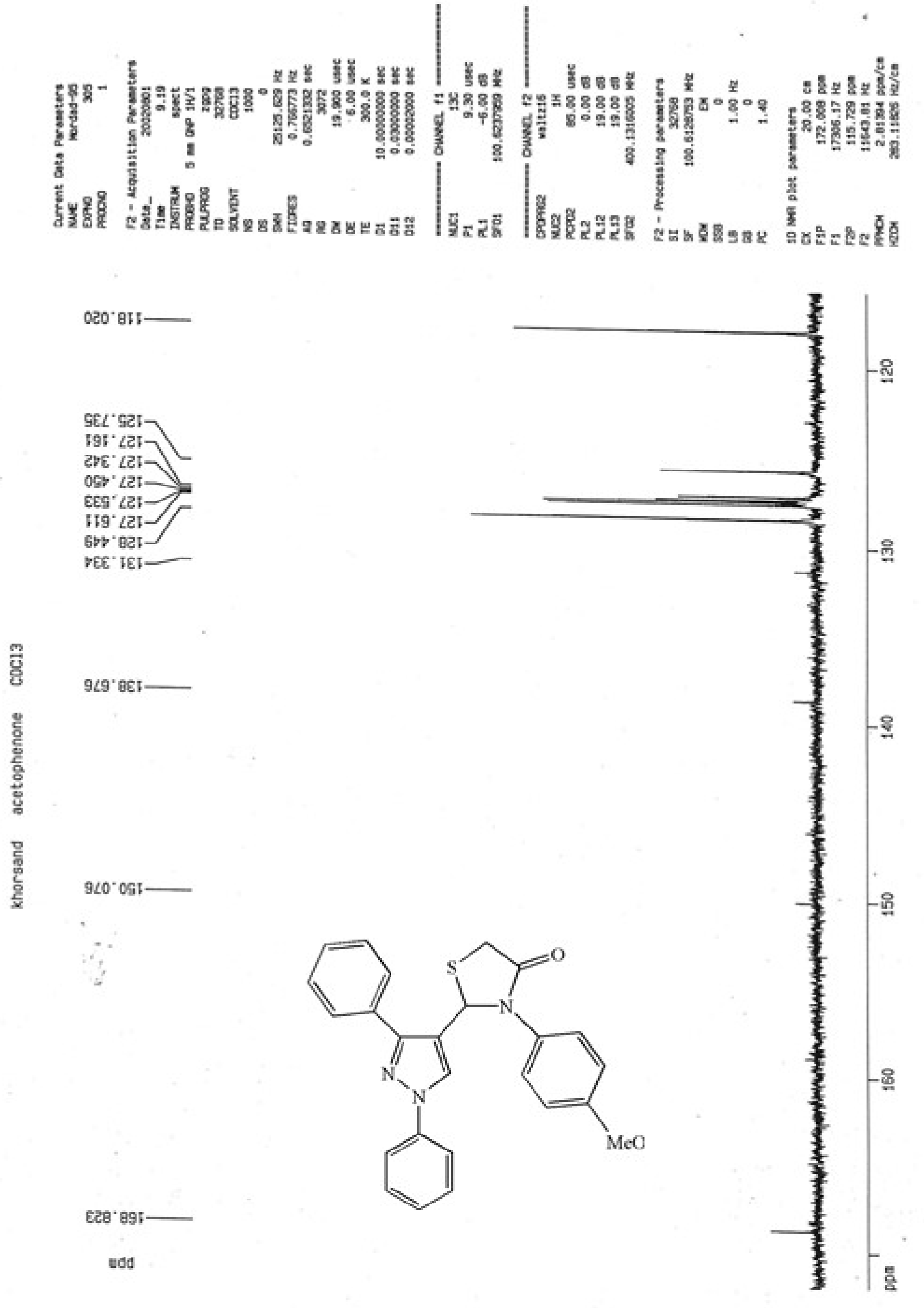
khorsand acetophenone CDCl3
118.050
125.735
127.161
127.342
127.450
127.533
127.611
128.449
131.334
138.676
150.076
168.823
ppm
S
O
N
MeO
120
130
140
150
160
ppm

طیف FT-IR ۲-(۱، ۳-دی‌فنیل-۱*H*-پیرازول-۴-ایل)-۳-(۲-متیل-۴-نیترو-فنیل)-تیازولیـدین-۴-اون(۴c)

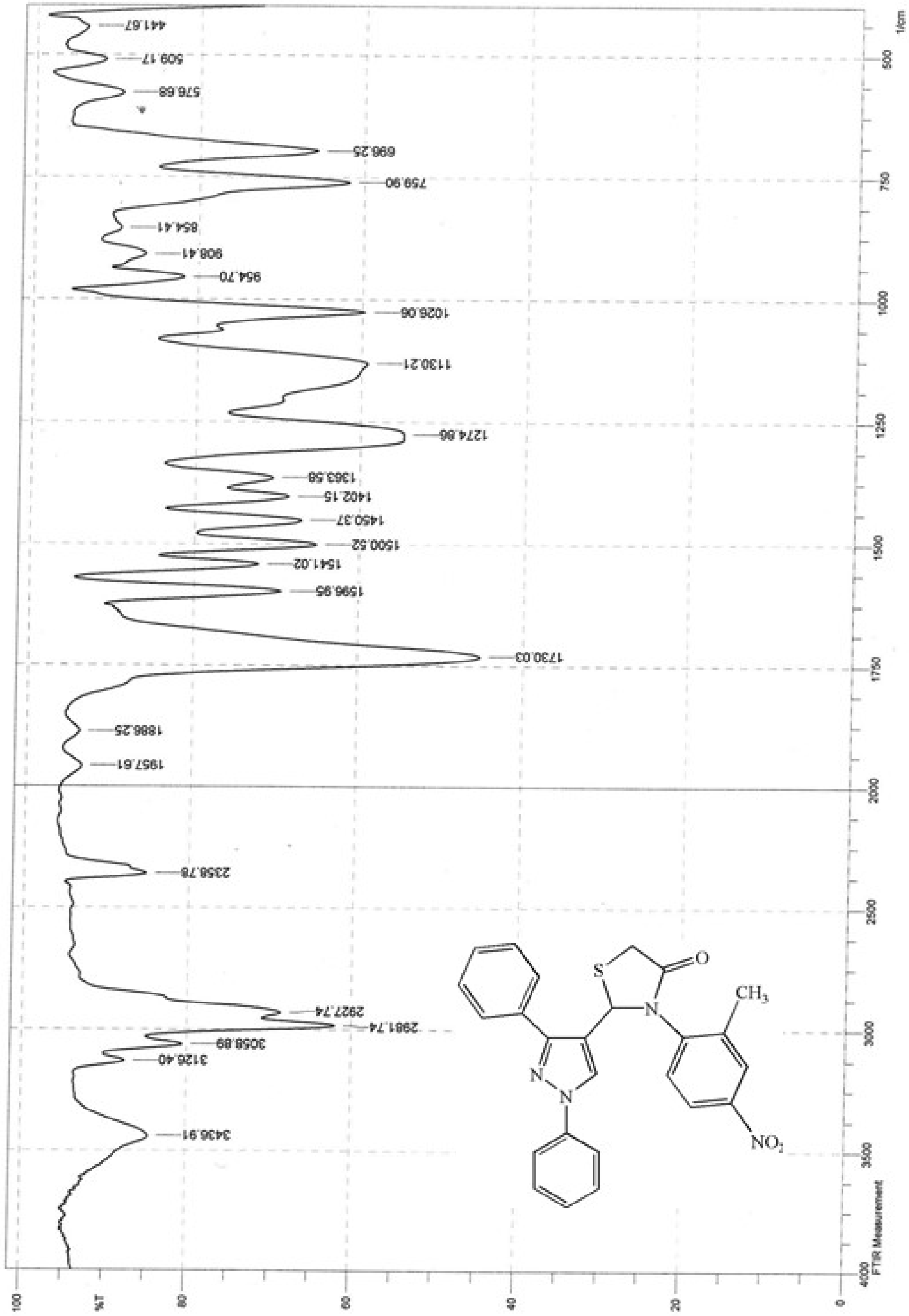

طیف ^{1}H NMR ۲-(۱، ۳-دی فنیل-*1H*-پیرازول-۴-ایل)-۳-(۲-متیل-۴-نیترو-فنیل)-تیازولیدین-۴-اون(۴c)

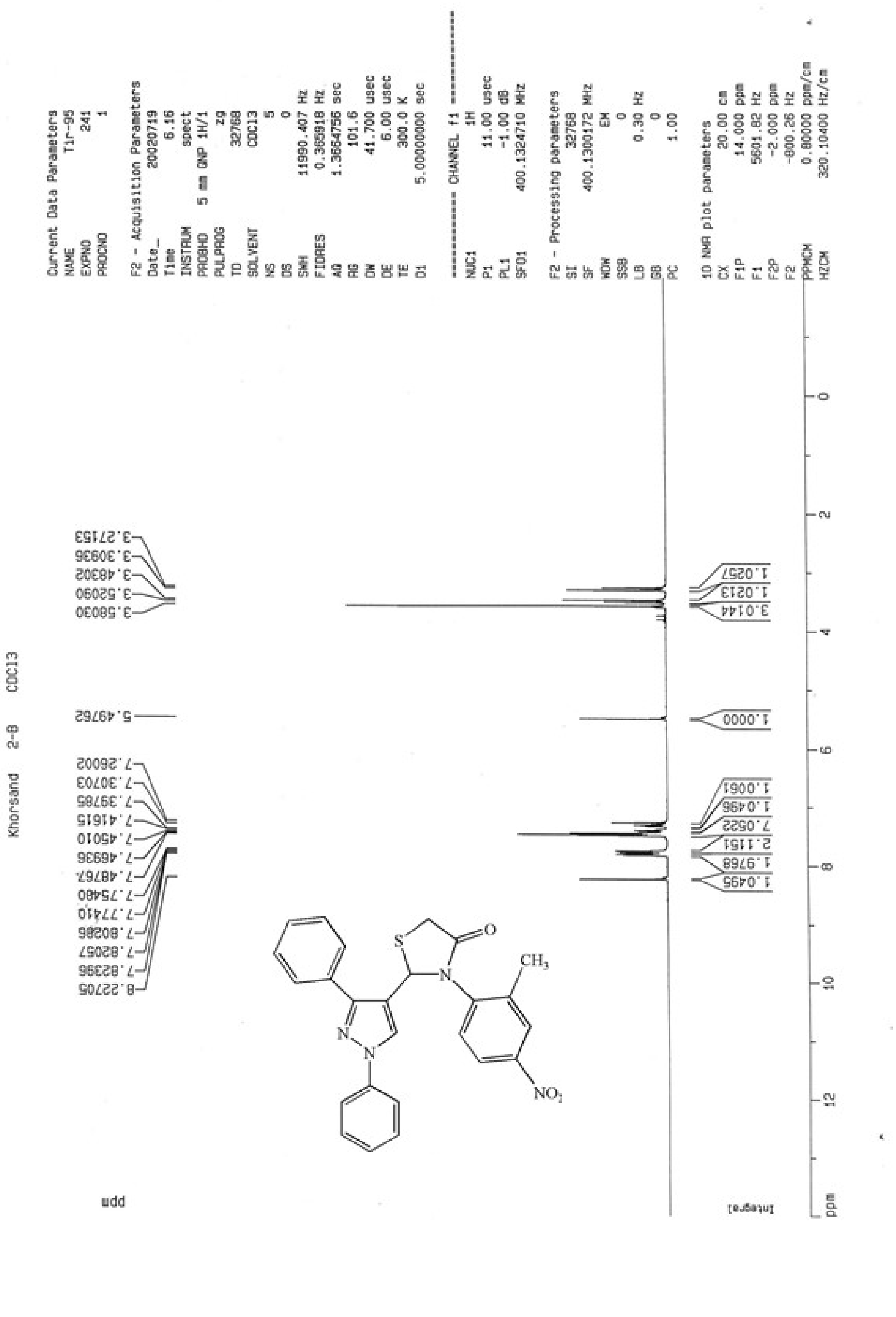

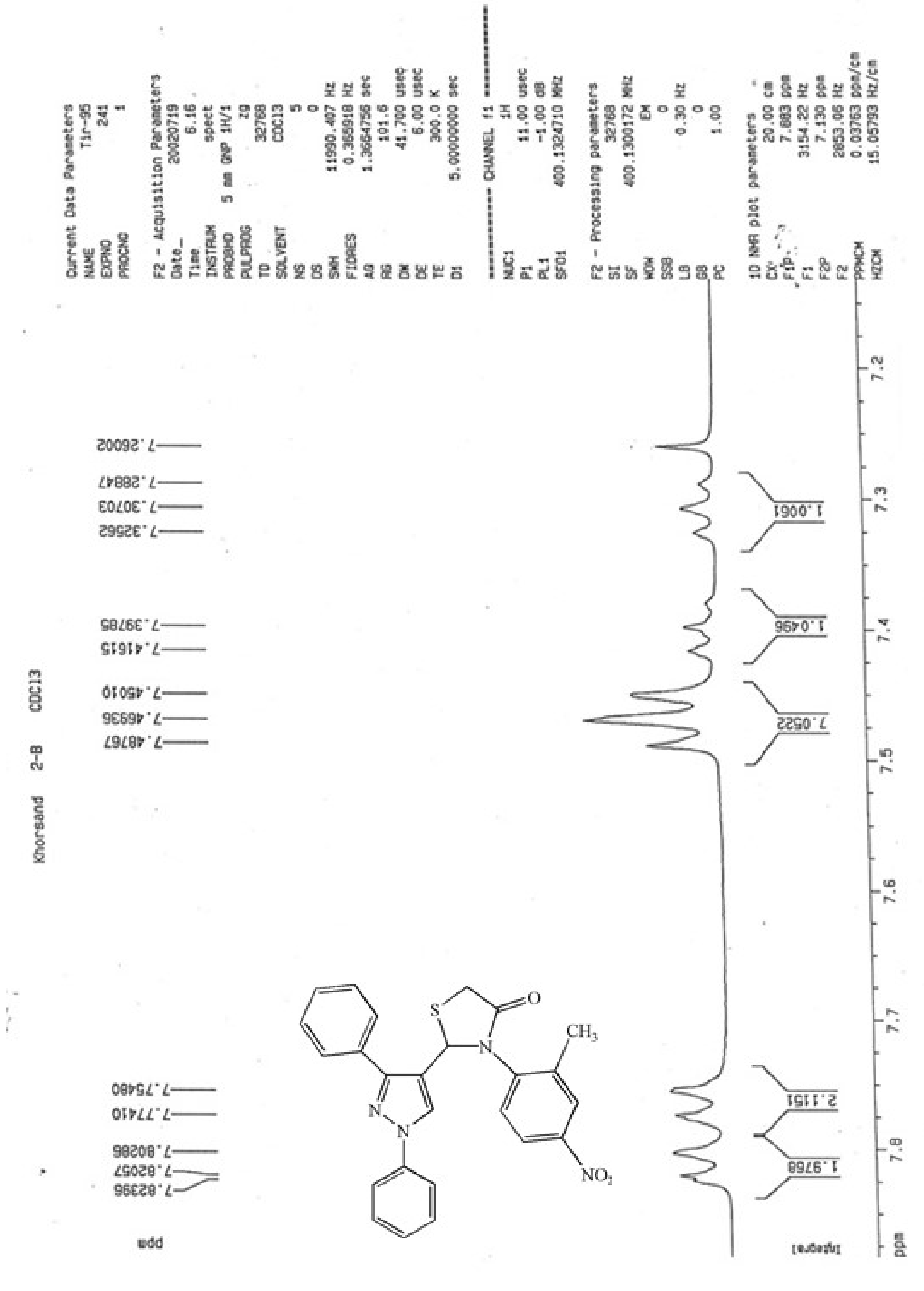
Khorsand 2-8 CDCl3
7.26002
7.28847
7.30703
7.32562
7.39785
7.41615
7.45010
7.46936
7.48767
7.75480
7.77410
7.80286
7.82057
7.82396
1.0061
1.0496
7.0522
2.1151
1.9768
CH3
NO2
ppm

طیف ^{13}C NMR ۲-(۱، ۳-دی فنیل-*1H*-پیـرازول-۴-ایـل)-۳-(۲-متیـل-۴-نیتـرو-فنیـل)-تیازولیدین-۴-اون(۴c)

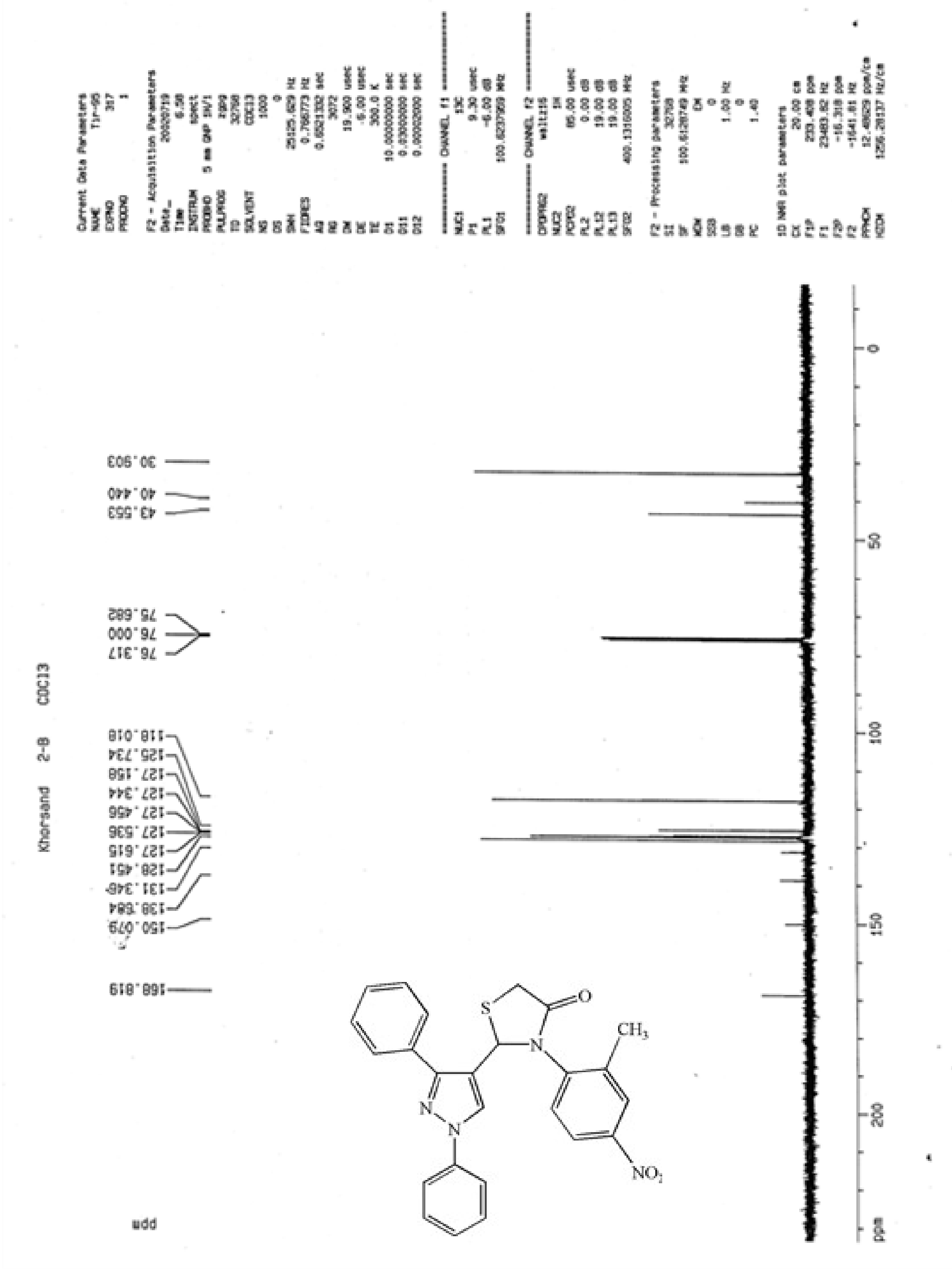

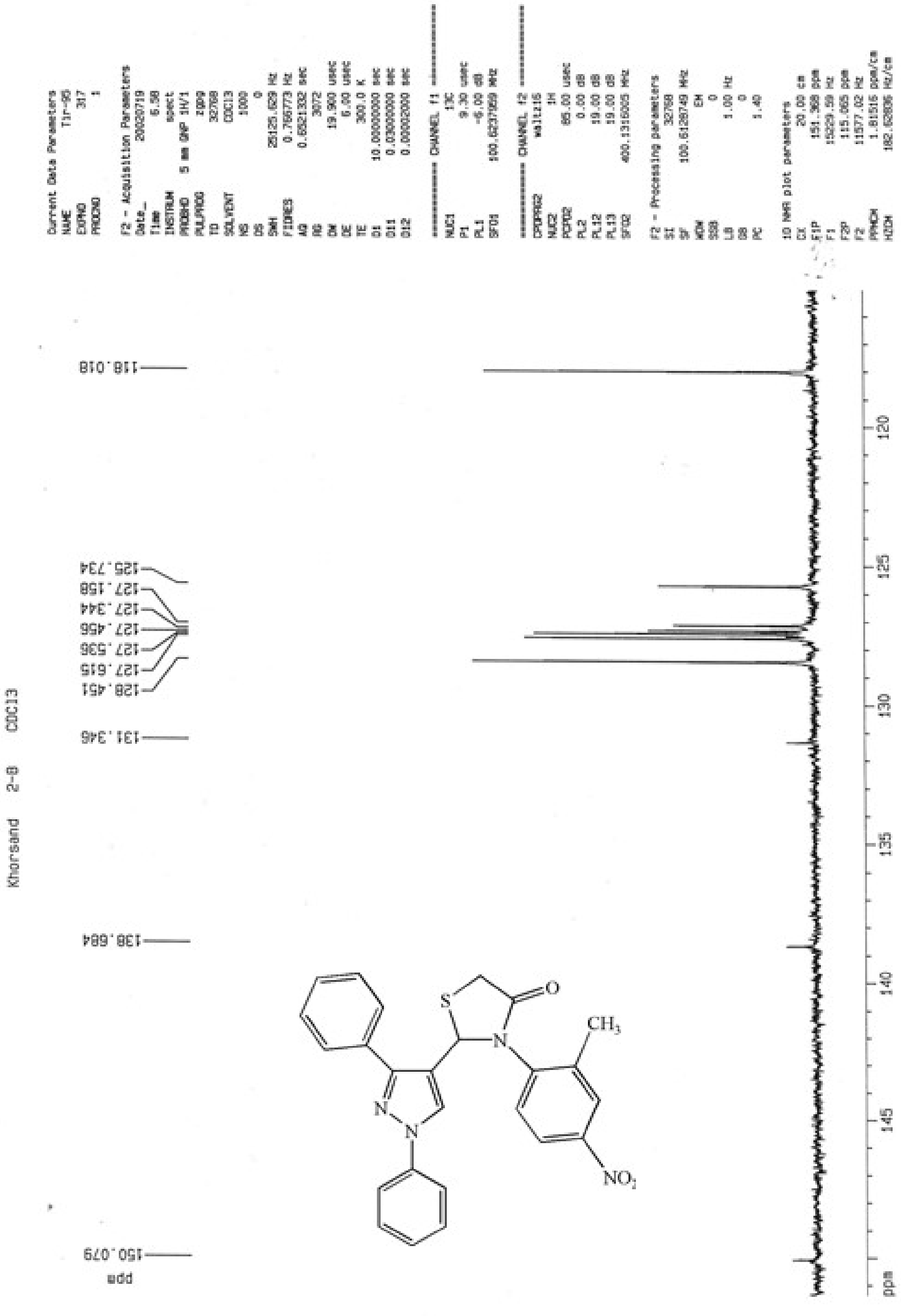
Khorsand 2-8 CDCl3
118.018
125.734
127.158
127.344
127.456
127.536
127.615
128.451
131.346
138.684
150.079
ppm
S
O
N
CH3
N
N
NO2

طیف FT-IR ۲-[۳-(۴-کلرو-فنیل)-۱-فنیل-۱*H*-پیرازول-۴-ایل]-۳-(۲-متیل-۴-نیترو-فنیل)-تیازولیدین-۴-اون(۴d)

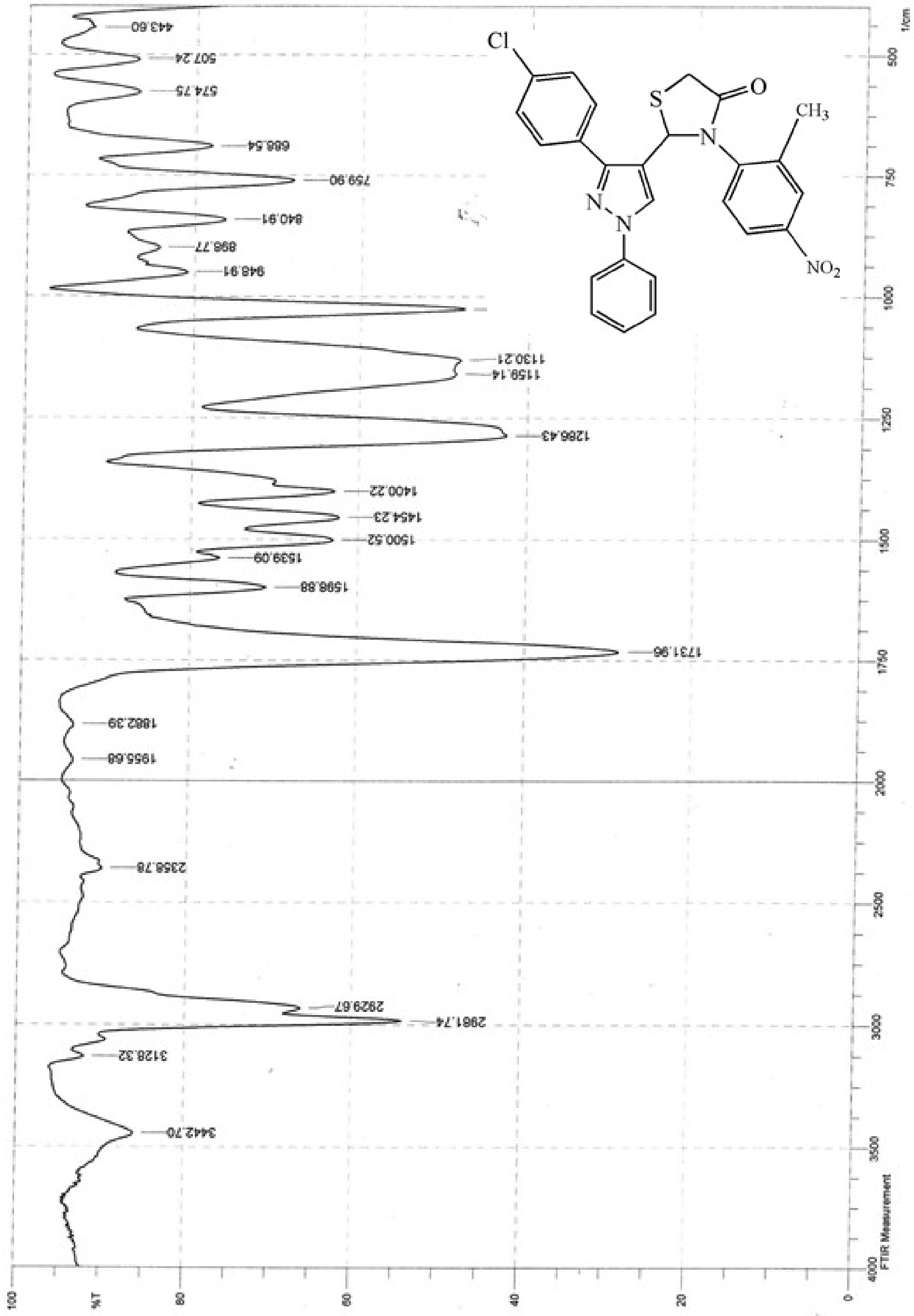

طیف ^{1}H NMR ۲-[۳-(۴-کلرو-فنیل)-۱-فنیل-*1H*-پیرازول-۴-ایل]-۳-(۲-متیل-۴-نیتـرو-فنیل)-تیازولیدین-۴-اون(۴d)

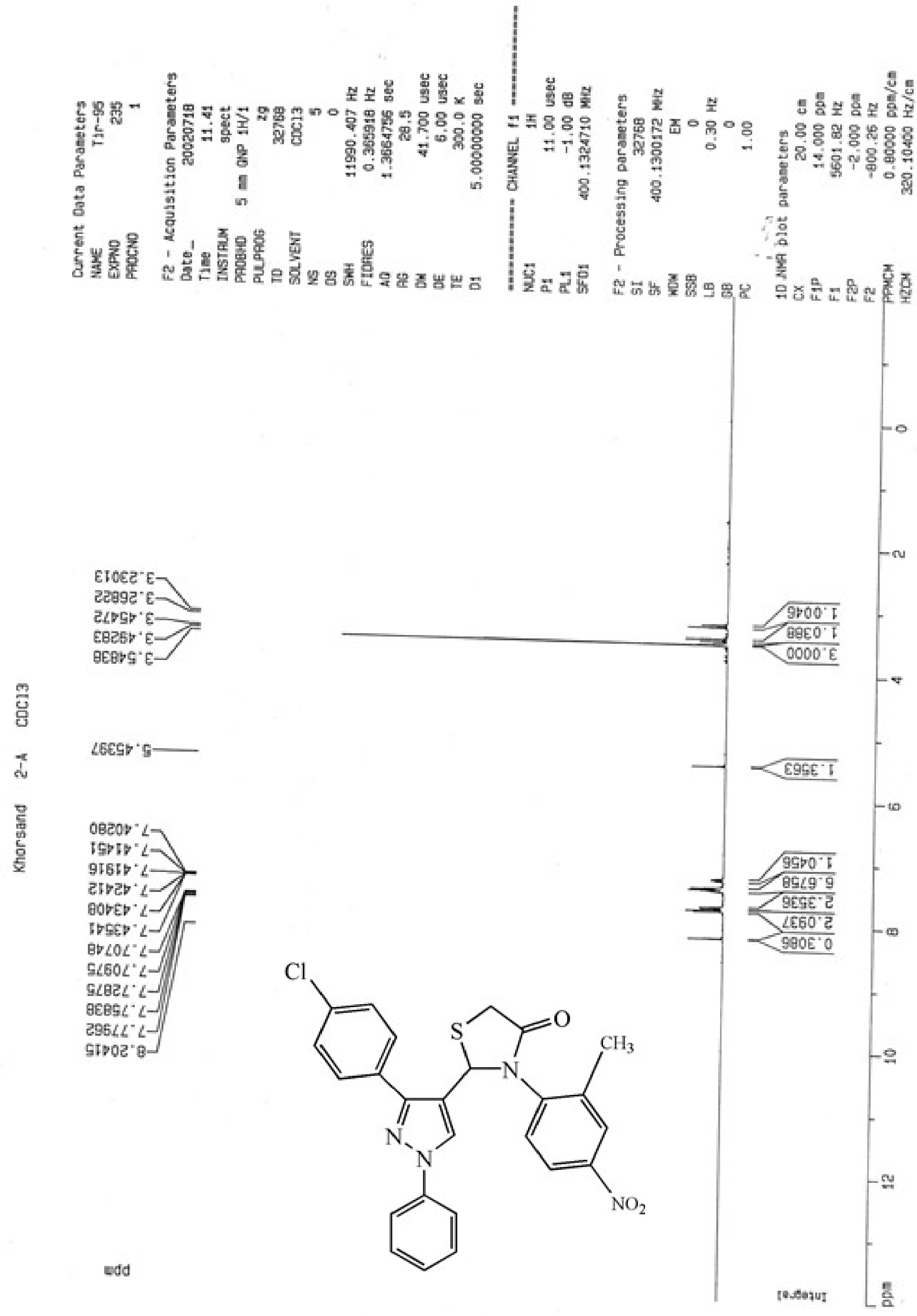

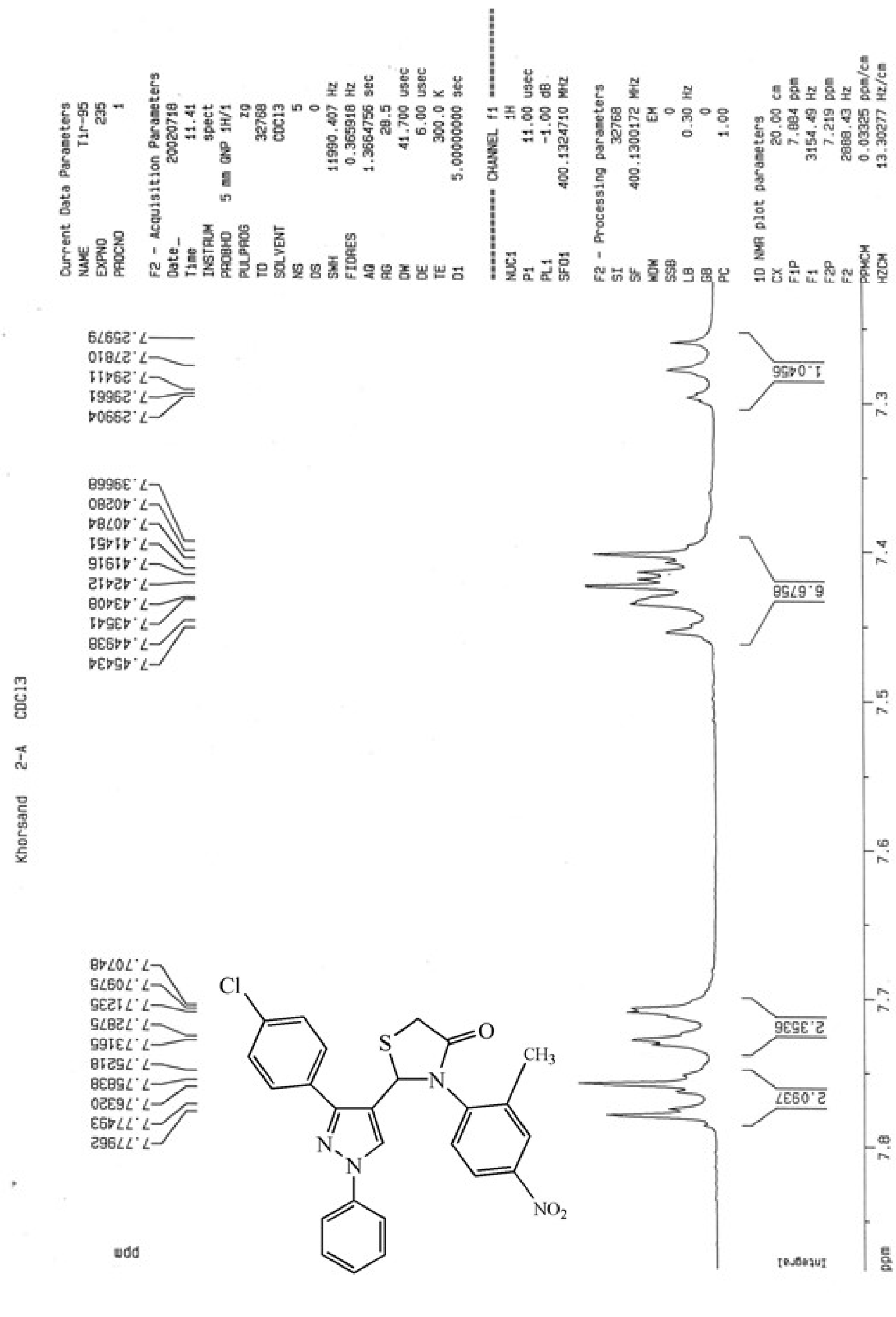
Khorsand 2-A CDCl3
Current Data Parameters
NAME Tir-95
EXPNO 235
PROCNO 1
F2 - Acquisition Parameters
Date_ 20020718
Time 11.41
INSTRUM spect
PROBHD 5 mm QNP 1H/1
PULPROG zg
TD 32768
SOLVENT CDCl3
NS 5
DS 0
SWH 11990.407 Hz
FIDRES 0.365918 Hz
AQ 1.3664756 sec
RG 28.5
DW 41.700 usec
DE 6.00 usec
TE 300.0 K
D1 5.00000000 sec
======== CHANNEL f1 ========
NUC1 1H
P1 11.00 usec
PL1 -1.00 dB
SFO1 400.1324710 MHz
F2 - Processing parameters
SI 32768
SF 400.1300172 MHz
WDW EM
SSB 0
LB 0.30 Hz
GB 0
PC 1.00
1D NMR plot parameters
CX 20.00 cm
F1P 7.884 ppm
F1 3154.49 Hz
F2P 7.219 ppm
F2 2888.43 Hz
PPMCM 0.03325 ppm/cm
HZCM 13.30277 Hz/cm
7.25979
7.27810
7.29411
7.29661
7.29904
7.39668
7.40280
7.40784
7.41451
7.41916
7.42412
7.43408
7.43541
7.44938
7.45434
7.70748
7.70975
7.71235
7.72875
7.73165
7.75218
7.75838
7.76320
7.77493
7.77962
1.0456
6.6758
2.3536
2.0937
Integral
7.3
7.4
7.5
7.6
7.7
7.8
ppm
Cl
S
O
N
CH3
N
N
NO2

طیف ^{13}C NMR ۲-[۳-(۴-کلرو-فنیل)-۱-فنیل-۱*H*-پیرازول-۴-ایل]-۳-(۲-متیل-۴-نیتـرو-فنیل)-تیازولیدین-۴-اون(۴d)

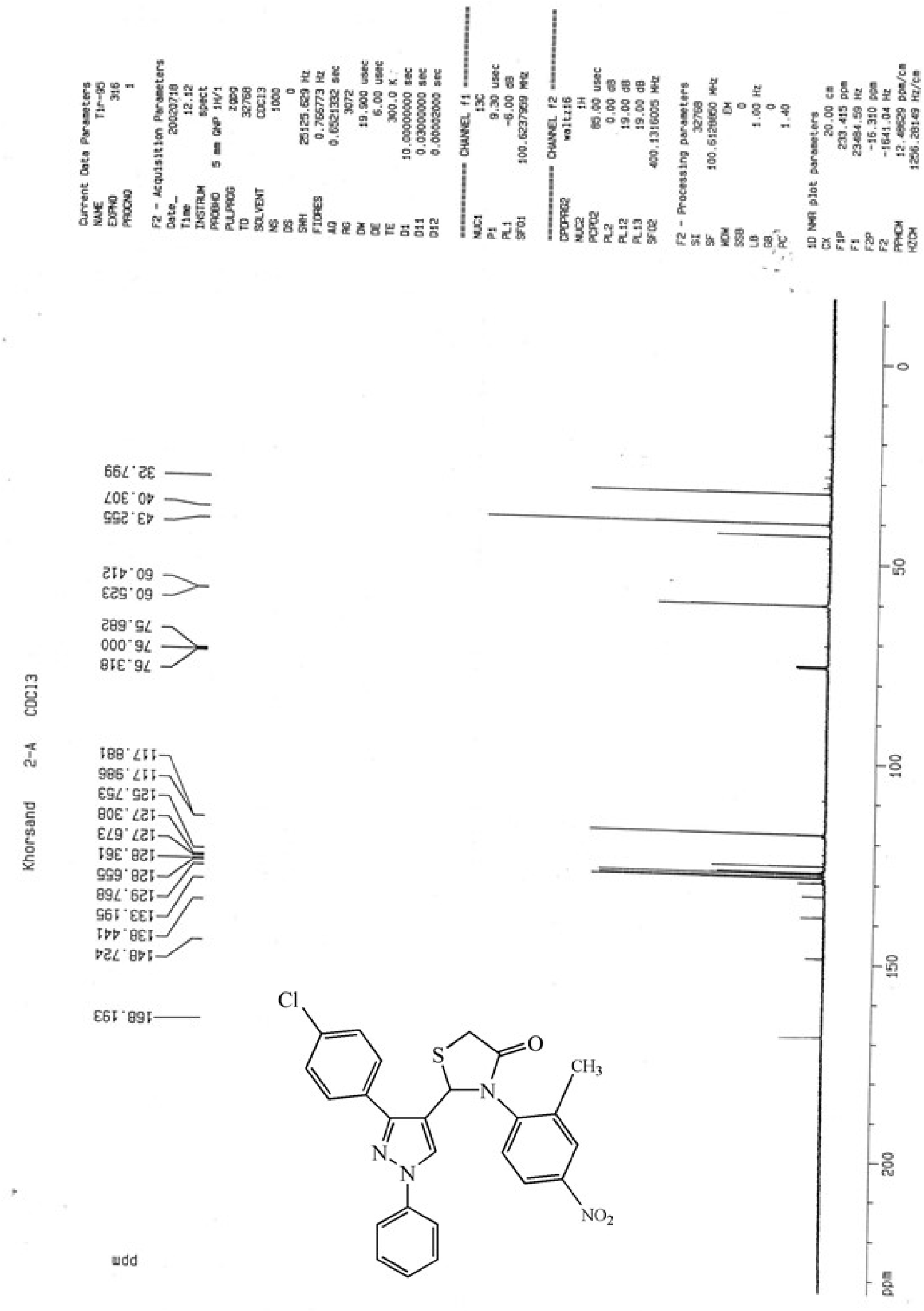

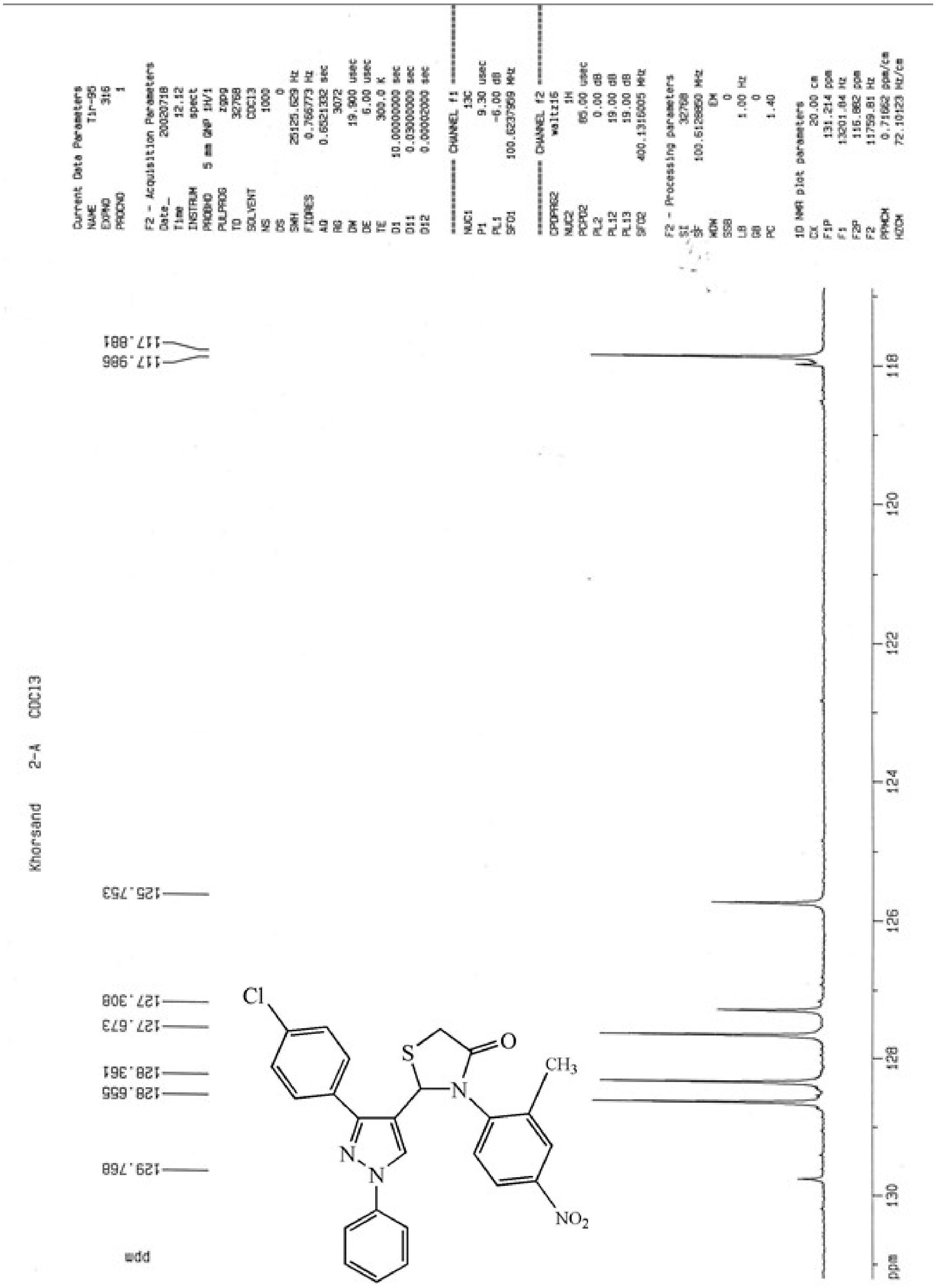
Khorsand 2-A CDCl3
117.881
117.986
125.753
127.308
127.673
128.361
128.655
129.768
ppm
118
120
122
124
126
128
130
ppm
Cl
S
O
N
CH3
N
N
NO2
